# Parallel computation of genome-scale RNA secondary structure to detect structural constraints on human genome

**DOI:** 10.1186/s12859-016-1067-9

**Published:** 2016-05-06

**Authors:** Risa Kawaguchi, Hisanori Kiryu

**Affiliations:** Department of Computational Biology and Medical Sciences, Graduate School of Frontier Sciences, University of Tokyo, 5-1-5 Kashiwanoha, Kashiwa, Chiba, 277-8561 Japan

**Keywords:** RNA secondary structure prediction, Parallel computation, PARS, Intron, Splicing

## Abstract

**Background:**

RNA secondary structure around splice sites is known to assist normal splicing by promoting spliceosome recognition. However, analyzing the structural properties of entire intronic regions or pre-mRNA sequences has been difficult hitherto, owing to serious experimental and computational limitations, such as low read coverage and numerical problems.

**Results:**

Our novel software, **“ParasoR”**, is designed to run on a computer cluster and enables the exact computation of various structural features of long RNA sequences under the constraint of maximal base-pairing distance. ParasoR divides dynamic programming (DP) matrices into smaller pieces, such that each piece can be computed by a separate computer node without losing the connectivity information between the pieces. ParasoR directly computes the ratios of DP variables to avoid the reduction of numerical precision caused by the cancellation of a large number of Boltzmann factors. The structural preferences of mRNAs computed by ParasoR shows a high concordance with those determined by high-throughput sequencing analyses.

Using ParasoR, we investigated the global structural preferences of transcribed regions in the human genome. A genome-wide folding simulation indicated that transcribed regions are significantly more structural than intergenic regions after removing repeat sequences and *k*-mer frequency bias. In particular, we observed a highly significant preference for base pairing over entire intronic regions as compared to their antisense sequences, as well as to intergenic regions. A comparison between pre-mRNAs and mRNAs showed that coding regions become more accessible after splicing, indicating constraints for translational efficiency. Such changes are correlated with gene expression levels, as well as GC content, and are enriched among genes associated with cytoskeleton and kinase functions.

**Conclusions:**

We have shown that ParasoR is very useful for analyzing the structural properties of long RNA sequences such as mRNAs, pre-mRNAs, and long non-coding RNAs whose lengths can be more than a million bases in the human genome. In our analyses, transcribed regions including introns are indicated to be subject to various types of structural constraints that cannot be explained from simple sequence composition biases. ParasoR is freely available at https://github.com/carushi/ParasoR.

**Electronic supplementary material:**

The online version of this article (doi:10.1186/s12859-016-1067-9) contains supplementary material, which is available to authorized users.

## Background

The existence of intronic regions is essential for producing the proteomic diversity of eukaryotes through alternative splicing (AS) [1]. To achieve such complex splicing events, most eukaryotes (except an intron-less nucleomorph genome [2]) are equipped with several types of spliceosomes. These complex molecular machines are composed of 5 snRNAs and more than 100 proteins [3]. Spliceosomes recognize splicing motif sites [e.g., two types of splice sites (SSs), donor and acceptor sites, and branch points], so that AS is carried out for introns with a wide range of lengths (from dozens to several tens of thousand nucleotides) in the context of nearly constant exon sizes [4].

Analytical determination of the features that spliceosomes recognize for proper splicing has been an important problem in the field of bioinformatics [5, 6], because AS abnormalities are involved in neuronal disorders and other diseases [3, 7, 8]. Computational approaches have revealed that functional SSs contain characteristic RNA secondary structures around them, in addition to well-known sequence motifs such as flanked GT-AG dinucleotides within introns [6, 9]. In previous research, such characteristics of secondary structure were required to attain a notably high accuracy of AS prediction [10]. An association between splicing and RNA secondary structure has also been validated by several experiments [11–13]. For instance, homologous *14-3-3 ζ* genes of insects were reported to need two types of complementary intronic sequence segments for mutually exclusive splicing, and the alternative exons that were present in the mature mRNA appeared to depend on the stability of their base pairings [14]. Accordingly, explaining the roles of RNA secondary structure in splicing completion and AS regulation is an important endeavor.

### Computational RNA secondary structure analyses around splice sites

Since mutation experiments with structure probing methods such as nuclear magnetic resonance spectroscopy or gel electrophoresis are time-consuming and laborious, very few studies have experimentally validated the complete secondary structures around SSs [12]. High-throughput structure analyses, such as PARS [15], have also been rarely applied to pre-mRNAs because of their paucity of sequencing reads mapped to the intronic regions [16]. Hence, computational prediction has significantly contributed to the comprehensive analyses of RNA secondary structures surrounding SSs.

These studies have revealed that the density of stable base pairs is regulated around SSs in complex ways. Around alternatively spliced exons, stable structures were shown to be over-represented and conserved relative to constitutive or skipped exons [17, 18]. At the same time, a significant enrichment of single-stranded transcript regions was also observed around splicing enhancer/silencer motifs [19]. This is presumably because splicing enhancer and silencer regions tend to contain binding sites of SR proteins and hnRNPs, which can regulate the splicing efficiency, and the exposure of such regions increases the binding efficiency of these splicing factors [20, 21].

### Difficulty in RNA secondary structure prediction of full-length introns

The ends of introns are known to be subject to complex structural constraints; however, little is known about the presence of structural constraints deep inside introns. Although the density of structural motifs of splicing factors will be low compared to the motif around the SSs, it is highly plausible that an intronic region far from the SSs also needs to satisfy various structural requirements for the normal progression of transcription, degradation, and splicing. A detailed structural analysis of intronic sequences would be useful to test the existence of such structural constraints, and would serve as a valuable aid to understanding what makes the introns different from intergenic regions. Nevertheless, very few studies have examined the structure propensity of full-length introns and pre-mRNAs, owing to the prohibitive time complexity of global structure prediction; the original mfold and McCaskill’s algorithms require $\mathcal {O}(N^{3})$ time complexity for input sequence length *N* [22, 23]. Because it is computationally infeasible to apply the algorithms to long RNAs, some folding programs restrict the allowed sequence distance between base pairing partners to within a given value *W* [24–26], which reduces the time complexity to $\mathcal {O}(NW^{2})$. Even with the maximal-span constraint, the computation time for long transcripts is prohibitive. A more serious problem is that the magnitude of the partition functions grows exponentially with the input length *N*, which can cause overflow or underflow errors when computing structural properties such as base-pairing probabilities and accessibilities (see Additional file 1: Chapter 3 for detail).

To circumvent these problems, sliding-window-based approaches have been developed, in which the folding algorithm is run for each artificial sequence window of length *L* in the input sequence [25, 27–29]. Because such algorithms are easily parallelizable and do not cause numerical errors as long as *L* is not excessively large, they can be a practical tool for genome-wide structure analyses under the current constraints of computational resources. For example, in Ref. [28], the authors used the minimum free energy (MFE) of each sequence window to investigate the structural preferences of transcribed regions. However, since it computes only the energy values of sliding windows and does not predict consistent secondary structures or stochastic structural indicators, detailed structural analyses such as the comparison with experimental data and investigation of the positional specificity of structural constraints were difficult. Other tools for genome-wide MFE-structure prediction using sliding-window approaches have similar problems [25, 27], because they were designed to search for unidentified short structural RNAs whose exact boundaries are unknown but not to analyze the structure propensity of a section of a continuous long RNA. As such, it has remained difficult to examine the positional structure propensity of introns using previous techniques that cannot handle an ensemble of possible structures for long transcripts.

### Our novel software ParasoR for genome-scale structure analyses

In this paper, we developed a novel software, **“ParasoR”**, which enables the distributed computation of various structural features of long RNAs based on the Boltzmann ensemble over globally consistent secondary structures. ParasoR divides dynamic programming (DP) matrices into smaller pieces, such that each piece can be computed by a separate computer node without losing the connectivity information between the pieces. ParasoR avoids the numerical problems of previous algorithms by directly computing the ratios of DP variables whose magnitudes are bounded independently of *N*. ParasoR can exhaustively compute structural features such as structural profiles [30] and globally consistent *γ*-centroid structures [31], as well as conventional base pairing probabilities, stem probabilities, and accessibilities. Using ParasoR, we investigated the structural preferences of entire transcribed regions in the human genome. To our knowledge, there is no exhaustive study examining the landscape of the structure stability of human introns using these probabilistic structural indicators. Our analyses demonstrate the potential of ParasoR to accelerate large-scale structural analyses performed *in silico*.

## Results

### ParasoR: A parallel solution for local RNA secondary structure analysis

ParasoR is our novel software application to exactly compute various expected values such as *stem probability* [23, 26] and *accessibility* [32–34] from the Boltzmann ensemble of global secondary structures, with the constraint of maximal base-pair span. We consider only the structures containing short-range base pairs, since it is well known that the energy model of the secondary structure is inaccurate for predicting distant base pairings [35]. The maximal span constraint limits the structure ensemble to the set of global secondary structures that contain only base pairs with spanning lengths ≤*W*. In Ref. [29], it is shown that the constraint of maximal span for the distance of base pairing can improve the accuracy of structure prediction. This constraint also reduces the computational complexity of structure prediction from $\mathcal {O}(N^{3})$ to $\mathcal {O}(NW^{2})$, as described in the Background section.

ParasoR is the only tool developed to date that can make global structure predictions for long RNAs (even for ∼3G base sequences). This high scalability of ParasoR is attained by the following two techniques: (1) solving numerical error problems by considering only the ratios of dynamic DP variables, and (2) allowing distributed computation for a computer cluster. Owing to its memory- and disk-saving design, ParasoR is also useful for small-scale studies that use a single computer.

**Fig. 1:**
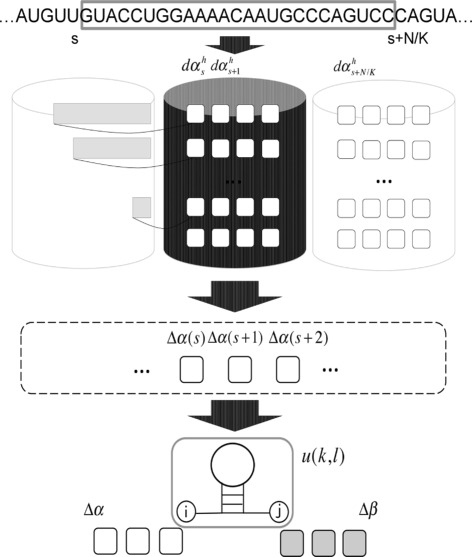
ParasoR overview illustration. A target sequence fragment is assigned to *K* computational nodes, and $d{\alpha ^{h}_{k}}$ is stored in external memory in the Divide procedure to solve the dependency problem that exists around the ends of a given fragment. In the Connect procedure, exact local fold changes *Δ* *α* are computed by the summation of $d{\alpha ^{h}_{k}}$ for each pairing pattern at the left end of the assigned fragment. In the computation of expected values, a variety of measures are available using the DP variables whose magnitudes are bounded independently of *N*, such as *u*(*k,l*), *Δ* *α*, and *Δ* *β*

Figure 1 shows a ParasoR’s workflow. In ParasoR, the structure prediction is carried out based on the Rfold grammar [26] and Inside–Outside algorithm. For a given set of sequences, ParasoR constructs a database of local fold changes of inside and outside DP variables *Δα* and *Δβ* through the Divide and Connect procedures (see the Methods section). From this database, ParasoR computes the following features for any queried region: (i) *base-pairing probability*; (ii) *stem probability*, represented as *p*_stem_(*i*) at *i*-th position; (iii) *accessibility*; (iv) *structural profilesp*_*δ*_(*i*), which represents the probability that the position *i* is a part of specific loop type *δ*=*bulge*, *exterior*, *hairpin*, *multi*, or *interior* [30]; and (v) a globally consistent secondary structure of credible base pairs (e.g., *γ*-centroid structure [31] with *γ*≤1).

This database can be used repeatedly for the fast structure simulation of similar but different sequences, such as those with point mutations or incomplete RNAs that appear during transcription elongation. ParasoR can also be applicable for the fast simulation of co-transcriptional splicing by using partial DP variables in the database that correspond to partially transcribed RNAs.

### Concordance of ParasoR prediction with validated Rfam structures and a high-throughput structure analysis

Since ParasoR was developed for the structure prediction of long RNA sequences, we tested its accuracy with the genome and mRNA sequences, using validated structures from the Rfam database [36] and a high-throughput structure analysis [37].

**Fig. 2:**
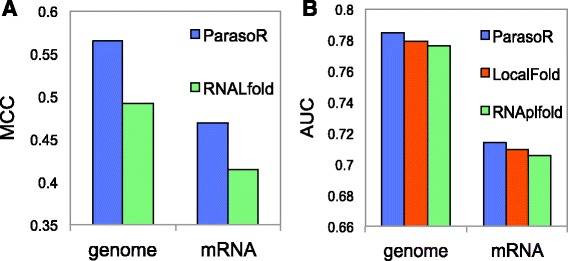
Accuracy comparison of single structure prediction. **a** MCC scores describing the structure predictions of *cis*-regulatory elements in the CisReg genome and mRNA dataset for the performance evaluation of ParasoR and RNALfold. **b** AUC scores describing the predictions of structured positions of *cis*-regulatory elements in the CisReg genome and mRNA dataset for stem probabilities of ParasoR and other tools

First, to evaluate the performance of structure prediction, we used CisReg data, which was compiled in Ref. [29] and contains high-quality sub-structures within long sequences. To construct the dataset, they searched the Rfam database for structures annotated as *cis*-regulatory elements, and obtained 2,500 structures, as well as the flanking mRNA or genomic sequences of lengths up to 3,000 nt on both sides. Then, we predicted secondary structures for these whole RNAs and compared them with known structures only within the region of target *cis*-regulatory elements. As for ParasoR, we used the *γ*-centroid structure with *γ*=1 [31]. Since RNALfold [24] does not predict a single consistent structure, we chose the longest non-overlapping structures for evaluation, and calculated the Matthews correlation coefficient (MCC) between predicted and correct base pairs (detailed in the “Methods” section). Figure 2a shows MCC scores of ParasoR for mRNA and genome datasets, which are substantially higher than those of RNALfold. It indicates the efficiency of *γ*-centroid structure prediction for long RNA sequences, as well as short RNA sequences [31], as they predict fewer false positives than the MFE-based method.

We also tested the accuracy of binary classification, which predicts whether each base is structural (base-paired) or accessible (unpaired), based on the stem probability for each position. This kind of problem is more meaningful when the input RNA does not take a single stable structure. For the stem probability *p*_stem_ computed by ParasoR LocalFold [29] and RNAplfold [25], we progressively changed a critical *p*_stem_ threshold and classified each position as structured or accessible, depending on whether *p*_stem_ was higher than the threshold or not. Then, we evaluated these classifications with the Rfam reference structure based on the area under the receiver characteristic operating curves (AUCs) (detailed in the Methods section and Additional file 1: Figure S19). Figure 2b shows that the AUC of ParasoR is higher than the other two tools for both the mRNA and genome datasets.

In summary, ParasoR is comparable to or better than the state-of-the-art algorithms for the prediction of stable motif structures such as *cis*-regulatory elements in long RNAs.

**Fig. 3:**
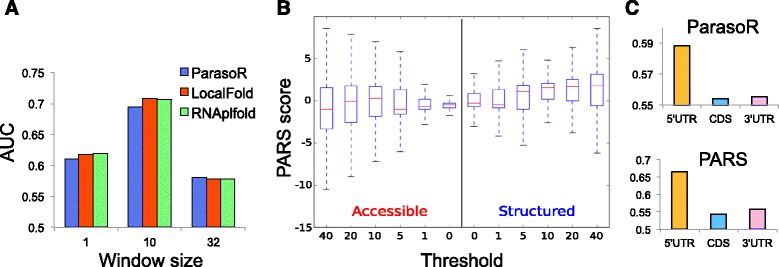
Comparison of stem probabilities and PARS scores. **a** AUC scores describing the prediction of positions with high PARS scores (i.e., structured regions) by stem probabilities for ParasoR and other tools. **b** Distribution of PARS scores for accessible and structured regions with varying read-depth thresholds. Each position was classified into Accessible or Structured depending on the stem probability of ParasoR (*p*_stem_<0.5 or *p*_stem_>0.5) after filtering of the minimum read-depth. Outliers are excluded from each Tukey boxplot. **c** Comparison of the average stem probabilities of ParasoR and the median of filtered PARS scores among 5^′^-UTR, CDS, and 3^′^-UTR categories

Next, we investigated the congruence between computational predictions and high-throughput structure analysis, using PARS data [37] in the same way as CisReg. To compare *p*_stem_ and PARS data from human mRNAs, we divided all nucleotide positions into two groups, accessible and structured, as determined by PARS scores. AUCs of three tools were then computed with a progressively changing *p*_stem_ threshold for their classification. Consequently, although all of the prediction methods showed a high consensus with the PARS-based classification, ParasoR had an almost comparable AUC score to LocalFold and RNAplfold (0.610 versus 0.618 and 0.619, respectively Fig. 3a (Left)). In addition, when we compared the 32-nt average of *p*_stem_ with the 32-nt average of PARS scores, ParasoR showed a slightly higher AUC than the other tools (0.581 versus 0.578 and 0.578, respectively Fig. 3a (Right)). These results are important, as we extensively study the distribution of such averaged *p*_stem_ in the later sections.

Because PARS scores with low-read depths are supposed to be less reliable, we set a threshold for the minimum read depth to filter out less reliable sites. For such a sample dataset, PARS score distributions were obtained for two groups, accessible (*p*_stem_(*i*)<0.5) and structured (*p*_stem_(*i*)>0.5) regions according to ParasoR-based *p*_stem_. Figure 3b shows that the PARS scores are more consistent in structured regions as their median values increase with the strictness of the threshold, while PARS scores fluctuate around zero in accessible regions, although they are consistent for a very strict threshold that requires ≥40 read counts to designate a site. Such filtering of low-depth regions actually increased overall AUCs for prediction methods up to approximately 0.75 (Additional file 1: Figure S17).

Since ParasoR has been developed to analyze genome-wide structure propensity, a consensus between ParasoR and PARS data in terms of structure propensity analyses was also tested for three categories of transcript region, 5^′^-UTR, 3^′^-UTR, and CDS. In Ref. [37], an average PARS score was used to estimate the likelihood of being structured for each region, and it was concluded that 5^′^-UTRs are less structured than 3^′^-UTRs and CDS. However, we found that the average PARS scores are affected by a small number of outliers with extremely large PARS scores. We therefore computed *median* PARS scores after the read depth filtering, and compared them with the mean *p*_stem_(*i*) for three categories (Fig. 3c). Both scores consistently indicate that 5^′^-UTRs have the highest stem density, whereas the stem density of CDS regions is the lowest. This high stem density of 5^′^-UTRs is mostly explained by their high GC content, as we show in the genome-wide propensity analysis later.

We have shown that the stem probabilities computed by the probabilistic folding methods such as ParasoR, RNAplfold, and LocalFold are highly consistent with the known structures of *cis*-regulatory elements and the PARS data. They also attain AUCs around 0.7-0.8 when ambiguous sites are removed from the PARS data. The differences of AUC scores among these programs are of the order of 0.01 and thus very small for these datasets.

Although ParasoR has similar accuracy to the other two tools, it is distinctly different from them, because it is a global folding method and the expected values such as stem probability and accessibility are computed from the Boltzmann ensemble of globally consistent secondary structures, as in McCaskill’s algorithm and RNAfold [38]. The difference between ParasoR and the latter two global folding algorithms is its scalability to handle long RNAs; ParasoR can compute the structural properties of the longest pre-mRNA in the human genome without any problem, whereas such computation is impossible for the other global algorithms, owing to the numerical errors and high time and space complexities.

In contrast to these global folding algorithms, RNAplfold and LocalFold average the probabilities that are computed from mutually inconsistent local RNA structures on different sliding windows. Although these sliding-window algorithms may capture the effects of structural obstacles such as bound proteins, introducing artificial boundaries at every sequence position may also cause artificial effects on the results. For example, it is known that accessibilities are artificially high close to the window boundaries [29], and both the window ends tend to be paired with each other (see Figures S5, S6, and S12 in the Additional file 1).

Another difference between the sliding-window methods and the global folding methods is that the probability distributions they produce are markedly different. As shown in Additional file 1: Figure S20, the distribution of ParasoR has bimodal peaks around probability 0 and 1, while the distributions of the other tools are more even. Furthermore, ParasoR has additional useful options that are not available in the other tools. For example, it can compute globally consistent *γ*-centroid structures, which are more accurate than MFE structures (Fig. 2a). The structural profile for each sequence position was also shown to be a very powerful means of understanding the complex structural specificities of RNA-binding proteins [30]. We therefore conclude that ParasoR is currently the most suitable program to analyze the structural properties of a transcriptomic-scale dataset with high confidence.

### Prediction of structure profiles for human transcript

**Fig. 4:**
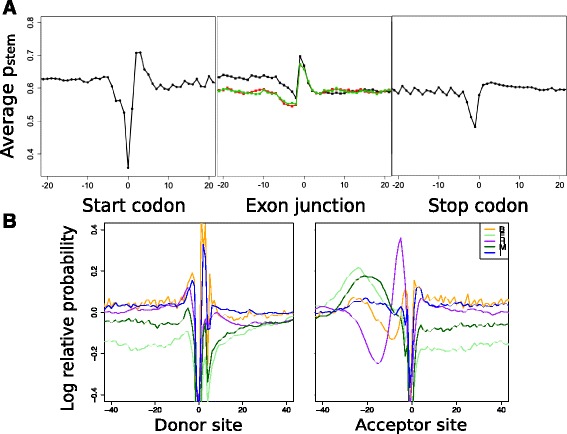
Structure profile of mRNA and pre-mRNA. **a** $\mu _{p_{\text {stem}}}(i)$ around start codons (*Left*), exon junctions (*Center*), and stop codons (*Right*). Profiles of the first, second, and third exon junctions are drawn in *black*, *green*, and *red*, respectively. **b** Log relative probability around donor sites (*Left*) and acceptor sites (*Right*) for Bulge (B), Exterior (E), Hairpin (H), Multi (M), and Internal (I) loops, which are represented by *orange*, *light green*, *purple*, *dark green*, and *blue lines*, respectively. Each position shows $\log (\mu _{p_{\delta }}(i) / \overline {\mu }{}_{p_{\delta }}) $ for the loop type *δ*. A 0 position indicates the starts of introns for donor sites, and the starts of exons for acceptor sites

We performed positional structure propensity analyses for human mRNAs and pre-mRNAs using ParasoR. To extract the common properties of human transcripts, we computed *μ*_*p*_(*i*), the positional profile of probabilities averaged across all human mRNAs or pre-mRNAs. Figure 4a shows $\mu _{p_{\text {stem}}}(i)$, the positional profile of stem probabilities around start codons, the 1st-3rd exon junctions, and the termination codon, which are computed from mRNA sequences. We consistently observed many characteristics reported in previous experimental analyses, such as the sudden fall of stem density before start and termination codons, an increase within start codons, and 3-mer periodicity in coding regions [37]. The higher stem probabilities upstream of the first SS can be explained by the GC-rich regions, such as CpG islands, around the first exons (Additional file 1: Figure S35).

Next, we analyzed the positional specificity for structural profiles $\mu _{p_{\delta }}(i)$ (*δ*= bulge, exterior, hairpin, or interior) computed from pre-mRNA sequences. Because the magnitude of $\mu _{p_{\delta }}(i)$ strongly depends on the loop type, we averaged $\mu _{p_{\delta }}$ across the 300 nt surrounding each SS on both sides to compute $\overline {\mu }{}_{p_{\delta }}$ and normalize the differences among loop types. In Fig. 4b, $\log (\mu _{p_{\delta }}(i) / \overline {\mu }{}_{p_{\delta }})$ is plotted to show the specific increase of loop probabilities around the donor and acceptor sites. Around donor sites, *p*_bulge_ and *p*_internal_ increase at position 1–3 nt, consistently with the two $\mu _{p_{\text {stem}}}$ peaks located at both sides of the donor sites (Additional file 1: Figure S34). Previously, several studies have reported the presence of conserved stable stem structures around donor sites [17, 39], and a stem-bulge structure upstream of the donor site is associated with the induction of Rex protein binding in HTLV-2 [40, 41] or the reduction of U1 snRNP binding in exon 10 of *tau* [42]. In contrast, the structural profiles around acceptor sites contain three separate peaks: *p*_hairpin_ at 3–9 nt upstream of the acceptor site, *p*_multi_ at 10–30 nt upstream of the acceptor site, and *p*_exterior_ at 13–40 nt upstream of the acceptor site. These peak locations are roughly within the polypyrimidine tract, which is the known binding site for U2AF and PTB [43]. In a previous study, the existence of loop structures was predicted to change the activity of the neighboring alternative acceptor sites in yeast [44]. Accordingly, such preferences for loop types generated by sequential motifs may help optimize the binding efficiency of constitutive splicing factors. As these preferences for specific loop types around motif sites have not been investigated in previous studies, identifying them and the splicing activity of each site can reveal unknown loop preferences optimized for binding a particular splicing factor.

### Genome-wide simulation to detect structural constraints on transcribed regions

Since the energy scale of secondary structure folding is high enough to influence the efficient progression of various biological processes, such as transcription elongation and translation, we expect many transcribed regions in the genome to be subject to various structural constraints. To study the structural preferences of transcribed regions relative to untranscribed regions, we computed the distributions of average stem probabilities $\bar {p}_{\text {stem}}(i)$ for 32-nt windows over both the strands of entire human chromosomes, and compared those of the different functional regions. This window size was chosen because it produced distributions that were close to the normal distribution for which statistical analyses are easier. Also, $\bar {p}_{\text {stem}}$ are expected to represent local structural features better than single-base stem probabilities (see the Methods section and Additional file 1: Chapter 3 and Additional file 1: Figure S8 for more discussion on choosing this window size).

**Fig. 5:**
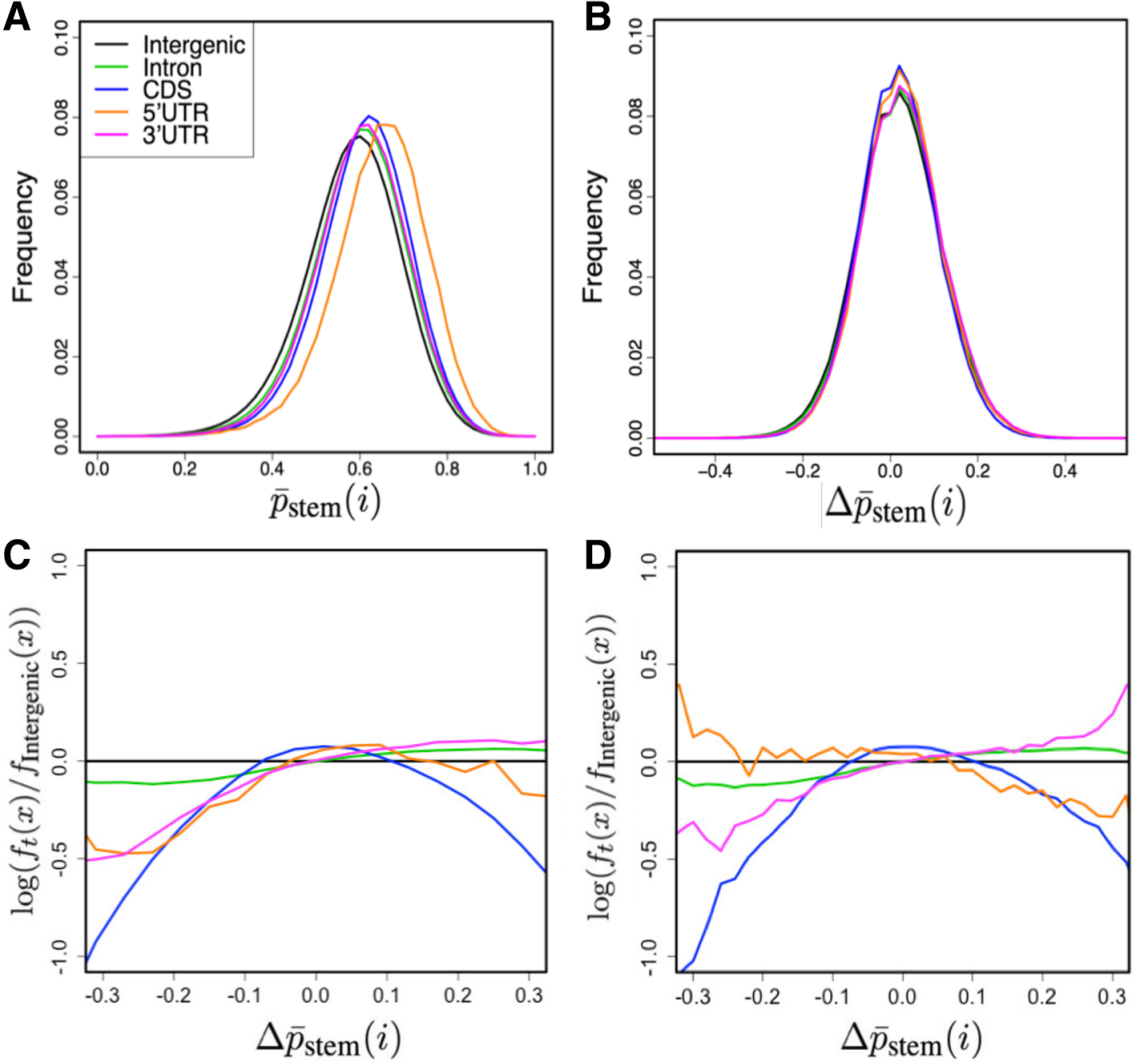
Structure propensity of genomic and transcriptomic regions. Intergenic regions, Intron, CDS, 5^′^-UTR, and 3^′^-UTR are represented by *black*, *green*, *blue*, *orange*, and *pink*, respectively. **a** Distributions of raw $\bar {p}_{\text {stem}}(i)$ for each annotation category. **b** Distributions of normalized average stem probabilities $\Delta \bar {p}_{\text {stem}}(i)$. **c** Log ratios of densities log(*f*_*t*_(*x*)/*f*_Intergenic_(*x*)), where *f*_*t*_(*x*) is the probability density of $\Delta \bar {p}_{\text {stem}}(i)$ at *x* for *t*=Intron,5^′^-UTR,CDS,3^′^-UTR. **d** is similar to (**c**), except that $\Delta \bar {p}_{\text {stem}}(i)$ was computed for the true boundaries of pre-mRNAs

Figure 5a shows the distributions of $\bar {p}_{\text {stem}}$ among five types of genomic regions: 5^′^-UTR, 3^′^-UTR, CDS, Intron, and Intergenic regions. Here, we removed repeat sequences elements from these regions. Further, the Intergenic regions are defined as the genomic regions that contain no repeat regions, no sense or antisense sequences of transcribed regions, and no sequences close to their boundaries (see the Methods section). All annotation categories exhibit a similar unimodal distribution. The 5^′^-UTR category apparently has the highest median, which is consistent with the elevated GC content around 5^′^-UTR regions [37]. The descending order of the stem probability medians (5^′^-UTR, 3^′^-UTR, and CDS categories) is the same as that of their structural strengths computed from mRNAs (Fig. 3c) in the previous subsection. It also shows all transcribed regions have higher median stem probabilities than those of the Intergenic regions, which may suggest the hypothesis that transcribed regions are constrained by their secondary structure. However, it should be noted that genomic sequences are also subject to various constraints that are unrelated to RNA secondary structure, and various characteristics of stem probabilities in transcribed regions may be side effects of sequence biases caused by such constraints. Therefore, we modeled the influences of sequence biases by training a linear regression model with $\bar {p}_{\text {stem}}(i)$ as targets and 4-mer frequencies as features. We then computed the normalized stem probability $\Delta \bar {p}_{\text {stem}}(i)$, which is the difference between $\bar {p}_{\text {stem}}(i)$ and its regressed value. This normalization mostly eliminated the differences in the medians among annotation groups so that the distinct difference in $\bar {p}_{\text {stem}}$ median values was explained by a sequence bias (Fig. 5b). Then, we focused on the residual part $\Delta \bar {p}_{\text {stem}}$, because large $\Delta \bar {p}_{\text {stem}}$ values represent structural preferences that are not merely explained by 4-mer frequencies.

To extract a faint structural propensity of each transcribed region in $\Delta \bar {p}_{\text {stem}}(i)$ compared to Intergenic regions, we plotted the log ratios log(*f*_*t*_(*x*)/*f*_Intergenic_(*x*)), where *f*_*t*_(*x*) is the probability density of $\Delta \bar {p}_{\text {stem}}(i)$ at *x* for *t*=Intron,5^′^-UTR,CDS,3^′^-UTR (Fig. 5c). As for the CDS regions, the density of this ratio is more concentrated around the center (Conover test, *p*<10^−1586^,*n*∼10^5^; the sample size is detailed in the Methods section), which indicates that the structural strengths in the CDS regions are more strongly determined by their base compositions than Intergenic regions. Additionally, introns and 3^′^-UTRs contain a higher rate of structured regions than that of Intergenic sequences, while 5^′^-UTRs, 3^′^-UTRs, and introns all exhibit lower rates of accessible regions. Figure 5d is similar to Fig. 5c, except that $\Delta \bar {p}_{\text {stem}}$ was calculated for pre-mRNAs, rather than for chromosomes. In this analysis, the distributions of introns, CDS, and 3^′^-UTR regions are qualitatively similar to those in Fig. 5c. In contrast, 5^′^-UTRs exhibit increased accessible regions in a wide range ($-0.3< \Delta \bar {p}_{\text {stem}} <0.0$) as compared to that shown in Fig. 5c, which is presumably because 5^′^-UTRs are shorter and more proximal to the transcript boundaries than are other regions.

**Fig. 6:**
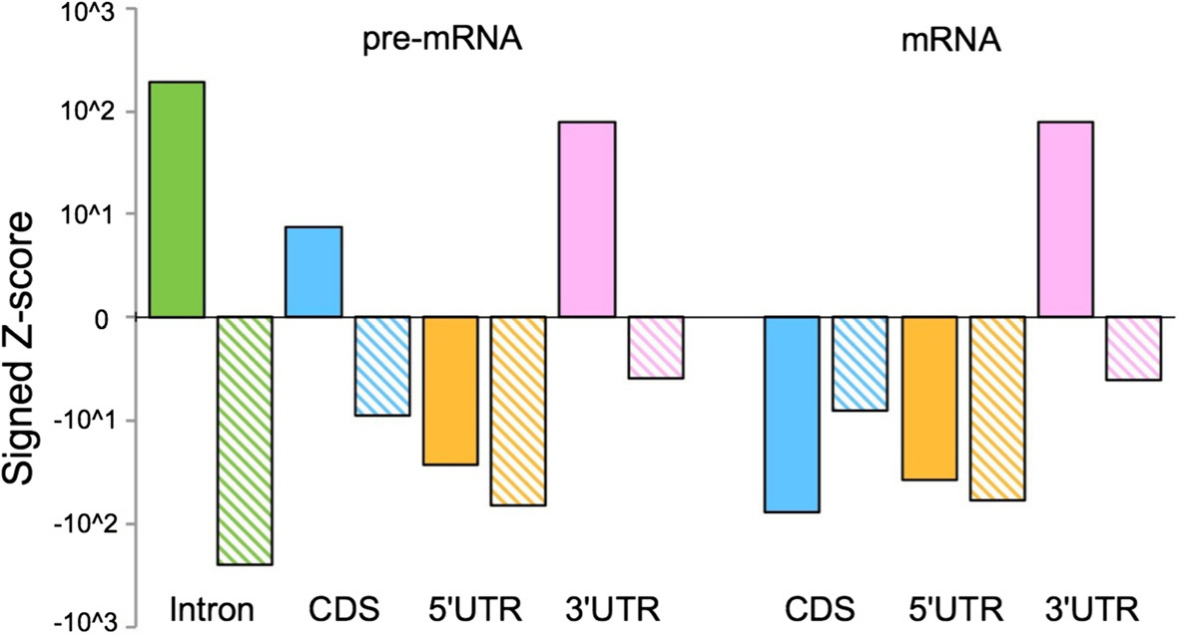
*Z*-score of structure propensity for each genomic annotation category relative to Intergenic regions. Wilcoxon’s rank sum tests were used to assess the structural preference of each genomic annotation category. A positive (negative) *y*-axis value indicates the annotation category has a higher (*lower*) stem density than that of Intergenic regions. Filled and shaded bars represent *Z*-scores of sense and antisense sequences of each annotation, respectively

To estimate the statistical significance of structural preferences of each annotation category relative to Intergenic regions, Wilcoxon’s rank sum test was applied to the distribution of $\Delta \bar {p}_{\text {stem}}$ for each annotation group, as well as their antisense sequences. Figure 6 shows the Z-scores of Wilcoxon’s rank sum tests; a positive (negative) value indicates the region contains a higher (lower) ratio of structured regions than that of Intergenic regions. We observed that the number of structured regions of introns is significantly higher than that of Intergenic regions at a significance level of *p*<10^−7940^ (*n*∼10^7^, calculated from *Z*-score with a one-sided test, hereafter). In contrast, the antisense sequences of introns significantly contain more accessible positions than Intergenic regions (*p*<10^−13655^,*n*∼10^7^). Antisense and sense sequences have the same GC content, but they can show a different strength of 4-mer normalization as well as $\bar {p}_{\text {stem}}$. Hence, such a different tendency of sense and antisense sequences cannot be explained by systematic differences in GC content or other strand-symmetric sequence features between the intron and intergenic regions. Additionally, 3^′^-UTRs exhibit the same trend, but at a lower significance level (sense: *p*<10^−151^, antisense: *p*<10^−940^, *n*∼10^6^). The 5^′^-UTR sequences possess more accessible positions than do Intergenic regions, which is consistent with Fig. 5d. When pre-mRNA and mRNA are compared, we observe that the *Z*-scores of CDS change from positive to negative (pre-mRNA: *p*<10^−13^,*n*∼10^6^, mRNA: *p*<10^−1301^,*n*∼10^6^), while 5^′^-UTRs and 3^′^-UTRs do not exhibit notable changes. The increased accessibility after splicing in the part of CDS regions suggests the existence of structural constraints in the particular mRNAs for translational efficiency or resistance to degradation [45].

**Fig. 7:**
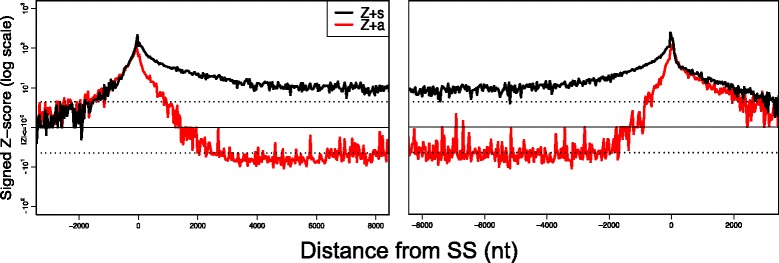
Positional profiles of structural preferences around splicing donor (*Left*) and acceptor (*Right*) sites. *Z*-scores of Wilcoxon’s rank sum statistics for the normalized average stem probability are drawn in *black* for sense and in *red* for antisense sequences. Dotted lines represent *Z*-scores that correspond to the Bonferroni-corrected *p*-values (<0.05) in a one-sided test

Positional dependency for these structural preferences was examined by computing the *Z*-scores for each 32-nt positional bin around the donor and acceptor sites in pre-mRNAs using Wilcoxon’s rank sum statistics (Fig. 7). Both sense and antisense strands exhibited a positive peak around the donor (*p*<10^−10054^ for 342,755 SSs) and acceptor (*p*<10^−13512^ for 326,618 SSs) sites. This pattern indicates structural constraints for splicing regulation [46], which is not easily explained by primary sequence biases. Inside exons, both sense and antisense *Z*-scores approach zero as the distance from SSs increases. In contrast, *Z*-scores for the sense strand remain positive within introns (8,000 nt downstream of the donor site, *p*∼10^−28^ for 26,970 SSs; 8,000 nt upstream of the acceptor site, *p*∼10^−9^ for 27,126 SSs), and those for the antisense strand become negative. As the *Z*-score for each bin was independently computed, the entire range of introns appears to be subject to structural constraints.

We note that a significant structural preference can be caused by only a small portion of transcribed regions, owing to the large degrees of freedom of the hypothesis tests. For example, our results suggest that the entire intronic regions are dispersed with small intronic elements that tend to be more structured as compared to controls, but this does not necessarily mean that the majority of introns forms highly stable structures. Despite these technical intricacies, we confirmed the same stem preference of intronic regions by different normalization methods, such as regression using GC content instead of 4-mer frequencies and block-wise-shuffled genome sequences instead of Intergenic regions as background (Additional file 1: Figures S28 and S32). Furthermore, we also found comparable results for the mouse genome, which implies that these trends are conserved among mammals (Additional file 1: Figure S33).

### Conformational changes caused by splicing events

**Fig. 8:**
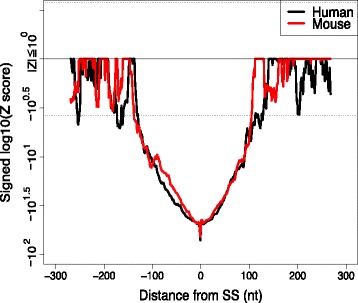
*Z*-score for structural differences caused by splicing events around SSs in human and mouse genomes. The difference of stem probability was averaged for a 32-mer sliding window separately for the upstream and downstream regions of SSs. The dotted line represents a *Z*-score of Wilcoxon’s signed rank test that corresponds to a significant Bonferroni-corrected *p*-value (<0.05) in a one-sided test

As described in the previous subsection, we investigated the structural preferences of transcribed regions by elaborate normalization procedures, such as masking repeat sequences, subtracting contributions from *k*-mer frequency bias by linear regression, and comparisons of functional regions with Intergenic and antisense regions. In this subsection, we investigate the structural changes after splicing performed by directly computing the difference of stem probabilities between mRNA and pre-mRNA as *Δq*_stem_(*i*)=*p*_stem,mRNA_(*i*)−*p*_stem,pre-mRNA_(*i*) for each exonic site upstream and downstream of SSs individually. Although this method cannot analyze intronic sequences, it has the advantage of constancy in the primary sequences for which stem probabilities are compared (detailed in Additional file 1: Chapter 7 and Additional file 1: Figure S36-39). Figure 8 shows the positional *Z*-scores of Wilcoxon’s signed rank test for *Δq*_stem_(*i*) of *p*_stem_ averaged by a 32-nt sliding window, where a negative (positive) *Z*-score indicates that a nucleotide position changes to be more accessible (structural) after splicing. Both human and mouse analyses show that splicing causes a significant reduction in stem density within approximately 100 bases around the SSs (*p*<10^−1111^ at the start position of the left side exon for 343,403 SSs). We also showed a consistent tendency in the case of *p*_stem_ using a single nucleotide window (Additional file 1: Figure S37).

To determine the gene features that are correlated with structural changes, we first computed the median and median absolute deviation of *Δq*_stem_ computed for each single exonic site within the 200-nt window around each SS. Then, we computed their correlation coefficients with the gene expression level, GC content of mRNA and pre-mRNA, and intron length (Table 1). A highly significant correlation with structural changes was found for gene expression and mRNA GC content. The magnitude of conformational changes also has a significant negative correlation with mRNA GC content.

**Table 1:** Correlation coefficients between conformational changes and gene features

Feature	Median	Median absolute deviation
Gene expression	-0.013**	0.010
mRNA GC%	0.008**	-0.007*
pre-mRNA GC%	0.029*	-0.004
Intron length	-0.004	-0.001

We tested statistical significance by Pearson’s correlation test. (*: *p*<0.05, **: *p*<1.0×10^−3^ after Bonferroni multiple correction.) The total number of tested SSs was 108,668 for the features of gene expression correlation coefficient and 261,161 for the other correlation coefficients

Finally, we studied enriched functional terms in gene sets that contain the SSs whose structure is dramatically changed through splicing events. We refer to the sites with the median of *Δq*_stem_(*i*) <0 and *Δq*_stem_(*i*)>0 as *post-accessible* and *post-structural* sites, respectively; then, we selected the top 10 % of post-accessible and post-structural SSs. We used the DAVID web tool [47] for an enrichment analysis of these groups, and identified several gene clusters with common functional categories. Table 2 shows the top three clusters for each test, where the expression analysis systematic explorer (EASE) score represents a significance measure for enrichment and corresponds to the negative log of the average *p*-value over the functional terms in a cluster. In the human genome, most post-accessible and post-structural genes are related to cytoskeleton and kinase, respectively, and the pattern is the same in the mouse genome. This may suggest that the genes associated with cytoskeleton, kinase, and other ATP-binding proteins are often post-transcriptionally regulated by the changes to their secondary structures.

**Table 2:** Enriched GO terms in post-accessible and post-structural genes

Gene set	EASE score	Keyword and GO term
Human	16.2	Cytoskeleton
post-	11.9	Kinase, ATP-binding
accessible	9.2	Centrosome
Human	8.2	Serine/threonine protein kinase
post-	5.5	C2 domain
structural	4.3	VWFA domain
Mouse	14.6	Cytoskeleton
post-	13.1	ATP-binding
accessible	7.3	Centrosome
Mouse	16.6	Kinase, ATP-binding
post-	6.1	Serine/threonine protein kinase
structural	4.2	C2 domain

### Conformational changes of the mRNA that encodes the F-actin binding protein

**Fig. 9:**
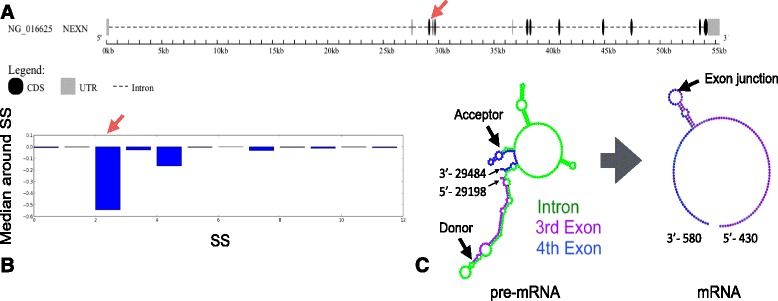
Gene structure and conformational change of the *NEXN* gene. **a** Gene structure of the *NEXN* gene. The GSDS tool has been used for visualization [57]. **b** Median difference of stem probability around each SS. The third SS shown with a red arrow is the most post-accessible SS among the genes in the cytoskeleton cluster. **c** Partial *γ*-centroid structure of pre-mRNA and mRNA (base pairs whose probability ≥0.5) around the third SS in the *NEXN* gene. For visualization, we only extracted the region of the substructure enclosed by the outermost pair or exterior loops around the 3rd intron in pre-mRNA and the 3rd exon junction in mRNA

Figure 9 shows the gene that has the most post-accessible SS in the cytoskeleton cluster according to the DAVID analysis. This *NEXN* gene (NM_144573) encodes nexilin, which is a filamentous actin-binding protein that functions in cell adhesion and migration. It has 12 SSs and several alternative splicing patterns, such as exon skipping at the 3rd, 6th, and 11th exons (Fig. 9b) [48]. Our analysis of stem probabilities suggests a large increase of accessibility through splicing at the 3rd SS (Fig. 9b). Figure 9c shows the secondary structures around the third and fourth exons of the *NEXN* gene, where we have depicted only the credible base pairs with probability ≥0.5. Before splicing, both the donor and acceptor sites form stems with intronic bases, while they are unpaired in the spliced mRNA structure. It is possible that the strong stems between exonic and intronic regions around the 3rd SS have important roles in regulating the observed AS patterns.

## Discussion

### Comparison between computational structure prediction and experimental structural analyses

The stem probabilities computed for human mRNAs agreed well with a large-scale experimental structural analysis in terms of both global characteristics (Figs. 3c and 4a) and statistical correlations between the scores (Fig. 3b). Although there are many reasons why computational folding fails to predict true secondary structures, most disagreement with experimental analyses currently seems to result from insufficient read depths. Thus, we expect more agreement with experiments as sequencing coverage continues to increase. We have also shown that the concordance of the prediction tool can be significantly increased by selecting appropriate averaging-window sizes or energy parameters that are suited to the experimental design and other conditions. It may even be possible to study the effects of pseudoknots or 3D structures by looking at the differences between computational predictions and experimental data that cannot be eliminated by such optimization.

### The influence of sequence biases on the analysis of structural constraints

A genome-wide comparison of thermodynamic structure stability would clarify the kinds of structural selection that act on the target regions. Simultaneously, such an analysis needs to employ an appropriate normalization scheme to eliminate primary composition biases. For example, the high GC content of CDS regions may lead to erroneous significance of selection pressure toward stable structures over the entire CDS regions, because the stability of RNA secondary structure is generally correlated with the GC content of a target sequence. Although there are several proposed methods to normalize such sequence biases (e.g., shuffling at a 4-fold degenerate site or preserving di-codon counts) [45, 49], they are mostly CDS-specific and could not be used in the present analysis. Intronic sequences are also known to possess several sequence biases, including the asymmetry of A/T and G/C around SSs [50], which does not cancel out by simply normalizing GC contents. As there is no perfect method to accomplish the normalization, we have taken a very conservative approach; we masked repeat regions, removed the contribution of *k*-mer frequency bias using a regression model, and compared the regions of interest with the Intergenic and antisense sequences. We have shown that the antisense sequences of introns are more different from the sense sequences than Intergenic regions in terms of structural propensity, which implies that the analyses that use only antisense sequences as background would overestimate the selection pressure. To complement this elaborate normalization approach, we have also carried out direct comparison between the same regions of mRNA and pre-mRNA to evaluate structure propensity inside exons, as they trivially do not possess any difference in the sequence composition bias.

### Structure propensity of genome sequences beyond *k*-mer composition effects

We determined that the stem density within CDS regions is better predicted by their sequence compositions (Figs. 5b and c) than are the stem densities of other regions, while introns and 3^′^-UTRs contain a significantly larger number of regions with higher stem densities than expected (Fig. 6). The strand-asymmetric preference for higher stem density persisted over entire intronic regions (Fig. 7), which cannot merely be explained by *k*-mer compositions or strand-symmetric sequence features. Such asymmetric preference is possible due to the strand-asymmetric pairing rules of the Turner energy model and other asymmetric sequence characteristics; G-U base pairing is not conserved in the complementary sequence and the appearance of different pairing patterns in loop regions such as the change from AAA to UUU. In Additional file 1: Section 7.4, we investigated the strand asymmetry of partition function and stem probabilities. They show a significant correlation between the strand asymmetry and the “GU” content of sequence (Additional file 1: Figures S40 and S41). We have also shown two examples in Additional file 1: Figure S42 and S43, in which strong stems in the sense strand are destabilized and decomposed into multi-loops in the antisense strand. The differences of folding energies between sense and antisense strands are also studied in Ref. [28].

As described previously, a significant structural preference can be caused by only a small portion of the transcribed regions, owing to the large degrees of freedom of the hypothesis tests. Therefore, our results suggest that the entire intronic regions are dispersed with small intronic elements that tend to be more structured than Intergenic and antisense sequences, but this does not necessarily mean that the entire regions of introns are highly structured. It should also be noted that we did not investigate the raw stem probabilities but the residual structural preferences remained after removing the sequence bias using linear regression. Therefore, the obvious correlation between stem probability and local GC content is normalized before the main analysis. Thus, the significant *p*-values supposedly reflect the intrinsic structural preferences beyond the obvious correlation between stem probability and local GC content.

One important future goal will be determining whether the known asymmetric mutation patterns in intronic regions [50] can explain this asymmetric structural preference. It will also be interesting to study various biological causes of the higher stem density within introns. It may prevent stalling of PolII (as in translation [51]), help splicing by shortening the physical distance between the donor and acceptor sites, or prohibit the splicing machine from accessing wrong acceptor sites.

A direct comparison of stem probabilities between mRNA and pre-mRNA showed a clear reduction in stem density around the SSs (Fig. 8). Our analyses indicate that this reduction is significantly correlated with the strength of gene expression. Together with the observation that SSs exhibit a strong structural preference (Fig. 7), these findings suggest that gene expression is mediated by the efficient use of secondary structures that disappear after pre-mRNA splicing.

## Conclusions

Using our novel software “ParasoR” and *k*-mer regression method, we extracted structure profiles of human transcripts and inferred the genome-wide structure propensity beyond sequence composition biases. The structure profiles predicted by ParasoR showed a high concordance with Rfam structures and high-throughput sequencing analyses. A genome-wide simulation using ParasoR indicated that a structure propensity of transcribed regions is strongly regressed by *k*-mer composition. By focusing on the residual part of such regression, intronic regions were shown to contain a significantly higher rate of structured regions compared to antisense and intergenic regions, not only around the ends of introns but also throughout entire regions. Furthermore, a comparison between pre-mRNAs and mRNAs suggested that coding regions become more accessible after splicing, presumably because of biological constraints such as translational efficiency.

## Methods

### ParasoR overview

For a given RNA sequence, ParasoR exactly computes various expected values from the Boltzmann ensemble of secondary structures under a maximal pair-distance constraint. It avoids numerical errors by dealing with only the ratios of DP variables, which do not change in magnitude as the sequence length *N* changes. To allow distributed computing, ParasoR divides the DP matrices into smaller pieces without losing their mutual dependencies. The computational complexities are given by either

1. $\mathcal {O}(NW^{2}/K+NW)$ time, $\mathcal {O}(N/K+W^{2})$ memory for each node, and $\mathcal {O}(NW)$ disk space or
2. $\mathcal {O}(NW^{2}/K+KW^{2})$ time, $\mathcal {O}(N/K+W^{2})$ memory for each node, and $\mathcal {O}(N+KW^{2})$ disk space, which requires less disk space than (i) but twice the computational time $\mathcal {O}(NW^{2}/K)$ for DP matrices construction.

Here, *N* denotes the input sequence length; *W* denotes the maximal span of base pairs; and *K* denotes the number of available computer nodes. We first analyze the dependency structures of DP variables on *N* and then rewrite the expected values using the ratios of DP variables, which do not change scales with *N*. Next, we describe how the computation of these variables is distributed across different computer nodes. For brevity, the explicit algorithms are described in Additional file 1: Chapter 1.

Any secondary structure *ζ* of sequence *x* is specified by a list of base pairs. In the conventional Turner energy model, a base pair of *x*_*k*_ and *x*_*l*_ must be one of the canonical base pairs {AU, UA, CG, GC, GU, UG}, and the distance between them should satisfy 5≤(*l*−*k*+1). We designate a position pair (*i,j*) an outermost pair if (*x*_*i*+1_,*x*_*j*_) forms a base pair and there is no base pair that encloses (*i,j*) in *ζ*. Since we impose the maximal span constraint, the outermost pair (*i,j*) also satisfies (*j*−*i*)≤*W*. Then, the structure *ζ* is uniquely decomposed into the set of non-overlapping substructures that are enclosed by an outermost pair for each and fragments of exterior loops between or flanking them. We define the set of potential outermost pairs of *x* as *P*={(*i,j*) | (*x*_*i*+1_,*x*_*j*_) is one of the canonical base pairs and 5≤*j*−*i*≤*W*}.

### Stem probability in Rfold algorithm

**Fig. 10:**
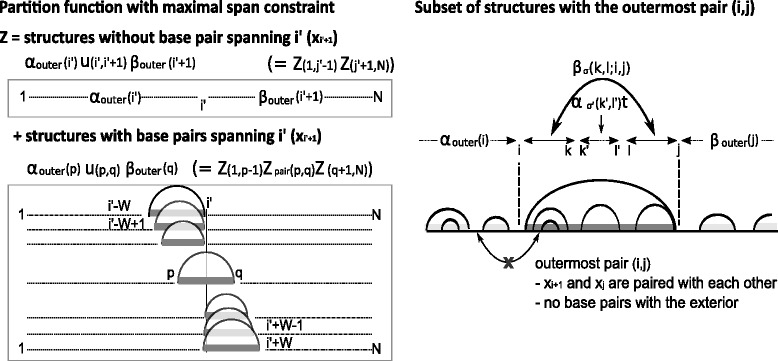
Schematic illustration of the decomposition of partition function and ParasoR algorithm. One example structure is expressed by the arcs representing the base pairs. By the summation of expected values for structures with each outermost pair, ParasoR can calculate an expected value from the ensemble of global structures

Throughout this article, we use a grammatical formulation of secondary structure developed in the Rfold model [26] (reproduced in Additional file 1: Section 1.1). In the Rfold model, there are 6 non-terminal symbols, in which the transition between Outer and Stem state corresponds to the transition from an exterior loop to the outermost base pair (see Fig. 10).

A partition function *Z* is calculated by an inside variable of Outer state *α*_Outer_ and Stem state *α*_Stem_ as follows.

$${\kern30pt} \begin{aligned} \alpha_{\text{Outer}}(j) & = \sum_{\zeta\in\Omega(1,j)}e^{dG(\zeta,x_{1,j})/RT} = \sum \left\{\! \begin{array}{ll} 1 ~\text{if}~j = 0 \\ \alpha_{\text{Outer}}(j-1) \cdot t(\text{Outer} \rightarrow \text{Outer}) \\ \alpha_{\text{Outer}}(k) \cdot \alpha_{\text{Stem}}(k, j) \cdot t(\text{Outer} \rightarrow \text{Outer} \cdot \text{Stem}) \\ \text{for}\ (j-W)\leq k<j \end{array} \right.\\ \beta_{\text{Outer}}(j) &= \sum_{\zeta\in\Omega(j+1,N)}e^{dG(\zeta,x_{j+1, N})/RT}  \\ Z &= \sum_{\zeta\in\Omega_{0}}e^{dG(\zeta,x_{1,N})/RT} = \alpha_{\text{Outer}}(N) = \beta_{\text{Outer}}(0)  \end{aligned} $$

Here, *dG*(*ζ*,*x*) represents the free energy for sequence *x* with structure *ζ*; *R* represents the gas constant; *T* represents the absolute temperature; *t* represents the Boltzmann factor for the transition; *Ω*_0_ and *Ω*(*k,l*) represent the set of all secondary structures of sequence *x* and subsequence *x*_*k,l*_ between *k* and *l*, respectively.

The expected values we consider are assumed to be given by the sum of state transition probabilities *p*(*σ*,*k,l*→*σ*^′^,*k*^′^,*l*^′^):

(1) $$\begin{array}{*{20}l} &p\left(\sigma,k,l\rightarrow\sigma',k',l'\right)=\sum_{\zeta\in\Omega(\sigma,k,l\rightarrow\sigma',k',l')}e^{dG(\zeta, x)/RT}/Z\\ &=\beta_{\sigma}(k,l)t\left(\sigma,k,l\rightarrow\sigma',k',l'\right)\alpha_{\sigma'}\left(k',l'\right)/Z \end{array} $$

where *σ* and *σ*^′^ represent non-terminal symbols of the Rfold grammar; *k*, *l*, *k*^′^, and *l*^′^ represent sequence positions; *Ω*(*σ*,*k,l*→*σ*^′^,*k*^′^,*l*^′^) represents the set of secondary structures containing the transition (*σ*,*k,l*→*σ*^′^,*k*^′^,*l*^′^); $\phantom {\dot {i}\!}\alpha _{\sigma '}(k',l')$ represents the inside variable; and *β*_*σ*_(*k,l*) represents the outside variable.

A type of expected values called the stem probability was extensively examined in this paper. The stem probability *p*_stem_(*i*) (which is equal to 1−accessibility(*i*)) at sequence position *i* is the probability that the base at position *i* is within a stem and is defined as $p_{\text {stem}}(i)=\sum _{j(>i)}p(i,j)+\sum _{j(<i)}p(j,i)$, where *p*(*i,j*) represents the base-pairing probability [23].

### Avoiding numerical problems by using a ratio of DP variable and partition function

In Eq. 1, the magnitudes of *α* and *t* do not change with *N*, since they are computed from the subsequence *x*_*k,l*_ whose length does not exceed *W*. *Z* and *β* are, however, the sums of Boltzmann factors for the subsequences of length $\mathcal {O}(N)$, and grow exponentially with *N*. On the other hand, the probability *p*(*σ*,*k,l*→*σ*^′^,*k*^′^,*l*^′^) should be between 0 and 1, and so a large cancellation between *β*_*σ*_(*k,l*) and *Z* must occur, which reduces the numerical precision. The cancellation is assured because the contributions from the structures far outside of (*k,l*) are almost the same. This can be seen from the following decompositions.

(2) $$\begin{array}{*{20}l}{\kern35pt} \beta_{\sigma}(k,l)=&\sum_{(i, j)\in P,\ i \leq k < l \leq j}\alpha_{\text{Outer}}(i)\beta_{\sigma}(k,l;i,j)\beta_{\text{Outer}}(j) \end{array} $$

(3) $$\begin{array}{*{20}l}  Z=& \alpha_{\text{Outer}}(i')t(\text{Outer}\rightarrow\text{Outer})\beta_{\text{Outer}}(i'+1) +  \\ & \sum_{(i,j) \in P,\ i \leq i' < j}\alpha_{\text{Outer}}(i)t(\text{Outer},i,j\rightarrow\text{Outer}\cdot\text{Stem},i,j)\alpha_{\text{Stem}}(i,j)\beta_{\text{Outer}}(j)\\ =& \sum_{(p,q) \in S(i')}\alpha_{\text{Outer}}(p)u(p,q)\beta_{\text{Outer}}(q)\\ &u(p,q)=\left\{\begin{array}{ll}t(\text{Outer}\rightarrow\text{Outer})&\text{if \(p+1=q\)}\\ t(\text{Outer},p,q\rightarrow\text{Outer}\cdot\text{Stem},p,q)\alpha_{\text{Stem}}(p,q)&\text{otherwise} \end{array}\right.  \end{array} $$

Here, *i*^′^ can be set to any position, the set *S*(*i*^′^) is defined as $\left \{ ({i,j}) \in P \ | \ i \leq i' < j \} \bigcup \{ (i',i'+1) \right \} $ for position *i*^′^, and *β*_*σ*_(*k,l;i,j*) are the outside variables for the subsequence located between the outermost pair (*i, j*), satisfying the initial condition *β*_Stem_(*i,j;i,j*)=*t*(Outer,*i,j*→Outer·Stem,*i,j*). Equation 2 follows, because the outside variables are the sum of the contributions of all possible patterns of outermost pairs. Equation 3 also follows, because a base represented by the position *i*^′^ is either within the outermost pair (*i,j*) or is an exterior base (illustrated in Fig. 10). It should be noted that the dynamic range (*i,j*)∈*P* in Eqs. 2 and 3 can be simplified to the set {(*i,j*) | *i*≤*j*,(*j*−*i*)≤*W*}, when the values of *β*_*σ*_(*k,l;i,j*) and *α*_Stem_(*i,j*) are zero for (*i, j*)∉*P*.

For those who are familiar with the partition function algorithms, it is noted that Eq. 3 for any position *i*^′^ is also represented by the decomposition of the partition function *Z* into the sum of those of smaller subsequences for any nucleotide position *j*^′^, as below.

$${\kern5pt} \begin{aligned} Z&= Z\left(1,j'-1\right)Z\left(j'+1,N\right)\\ &\quad+\sum_{(i,j) \in P',\ i \leq j' \leq j} Z(1,i-1) Z_{\text{pair}}(i,j) Z(j+1,N) \end{aligned} $$

Here, *P*^′^ is the set {(*i,j*) | (*x*_*i*_,*x*_*j*_) is one of the canonical base pairs, 5≤(*j*−*i*+1)≤*W*}, *Z*(*k,l*) is the partition function for subsequence *x*_*k,l*_, and *Z*_pair_(*k, l*) is the partition function of subsequence *x*_*k,l*_ with an outermost pair between *x*_*k*_ and *x*_*l*_ (note that *Z*(1,*i*−1) and *Z*(*j*+1,*N*), etc., actually need to include the contributions of dangling or mismatch scores that depend on the exterior bases outside of the sequence ranges).

Next, we define the ratio of the DP variables and partition function *r*(*i,j*) for any position pair (*i, j*) such that *i*≤*j*,(*j*−*i*)≤*W*:

$$\begin{aligned} r(i,j)&:=\frac{Z}{\alpha_{\text{Outer}}(i)\beta_{\text{Outer}}(j)} \\ &=\frac{\sum_{p, q \in S(i)}\alpha_{\text{Outer}}(p)u(p, q)\beta_{\text{Outer}}(q)}{ \alpha_{\text{Outer}}(i)\beta_{\text{Outer}}(j)}  \\ &=\sum_{p, q} \left\{ \frac{\alpha_{\text{Outer}}(p)}{\alpha_{\text{Outer}}(i)} \ u(p, q) \ \frac{\beta_{\text{Outer}}(q)/\beta_{\text{Outer}}(i)}{\beta_{\text{Outer}}(j)/\beta_{\text{Outer}}(i)} \right\} \\ &=\sum_{p, q} \left\{ \prod_{h=p}^{i-1}\Delta\alpha(h) \right\} \ u(p, q) \ \left\{\prod_{h=i}^{q-1}\Delta\beta(h)\right\} /\left\{ \prod_{h=i}^{j-1}\Delta\beta(h) \right\} \\ &\Delta\alpha(h):=\alpha_{\text{Outer}}(h+1)/\alpha_{\text{Outer}}(h)\\ &\Delta\beta(h):=\beta_{\text{Outer}}(h)/\beta_{\text{Outer}}(h+1) \end{aligned} $$

In our implementation, *Δα* and *Δβ* are stored as logarithmic values; hence, the summations in the above formula are replaced by logsum operations. In Additional file 1: Chapter 1, we show a DP algorithm that directly computes these values without recourse to *α*_Outer_ and *β*_Outer_. On the other hand, inner variables *u*(*p,q*) can be computed without numerical difficulties by using the ordinary inside algorithm. In this manner, we can avoid the computation of variables that exponentially increase with *N* for *r*. Then, the fold change *β*_*σ*_(*k,l*)/*Z* can be represented by the outside variable for a subsequence between *i* and *j* ($|j-i|=\mathcal {O}(W)$) and *r*(*i,j*), as below.

$$\begin{array}{*{20}l} \beta_{\sigma}(k,l)/Z &= \sum_{(i,j) \in P,\ i\leq k < l\leq j}\beta_{\sigma}(k,l;i,j) /r(i, j) \end{array} $$

In this way, we can compute an expected value only by the variables whose absolute values are bounded independently of *N*.

### Dividing computation into small jobs for parallelization

If we have a database of *Δα* and *Δβ* for a given sequence, we can obtain any probability of Eq. 1 for any subsequence with $\mathcal {O}(W^{3})$ time by locally reconstructing *r*(*i,j*). The computation of fold changes *Δα* and *Δβ*, however, requires $\mathcal {O}(NW^{2})$ time, which is equivalent to that for computing *α*_Outer_ and *β*_Outer_. We divide this computation into smaller pieces and process them in parallel (via the *Divide* procedure). Then, we merge the results and build the fold-change variables *Δα* and *Δβ* (via the *Connect* procedure).

In the Divide procedure, we break the input sequence into *K* subsequences and assign them to *K* computer nodes. To see how this can be done, we note the following formula that applies to any given position *s* (0≤*s*≤*k*), which is an extension of Eq. 3.

$$\begin{array}{*{20}l} \alpha_{\text{Outer}}(k)&=\sum_{(i,j) \in S(s),\ i\leq s < j \leq k}\alpha_{\text{Outer}}(i)u(i,j)\beta_{\text{Outer}}(j)^{(k)}\\ &=\sum_{h=0}^{\min(W, s)}\alpha_{\text{Outer}}(s-h){\alpha^{h}_{k}}, \end{array} $$

where *β*_Outer_(*j*)^(*k*)^ is *β*_Outer_(*j*) computed for sequence *x*_*j*+1,*k*_. For *h*>0, ${\alpha ^{h}_{k}}$ is the inside variable that starts from an outermost pair (*s*−*h,j*), such that *j*>*s*. For *h*=0, ${\alpha _{k}^{h}} =\beta _{\text {Outer}}(j)^{(k)}$. For each assigned subsequence *x*_*s,e*_, the values ${\alpha ^{h}_{k}}$ (0≤*h*≤*W, s*≤*k*≤*e*) are computed independently of other nodes, and in the Connect procedure, the complete *α*_Outer_ is recovered using the above formula. ParasoR combines this parallelized algorithm using the ratio of DP variables and partition function outlined in the previous subsection by directly computing the ratios $d{\alpha ^{h}_{k}}={\alpha ^{h}_{k}}/{\alpha ^{0}_{k}}$ using a DP procedure (Additional file 1: Chapter 1). The time complexities are $\mathcal {O}(NW^{2}/K)$ to compute $d{\alpha ^{h}_{k}}$ and $\mathcal {O}(NW)$ to construct *Δα*. To store $d{\alpha ^{h}_{k}}$, the memory complexity is $\mathcal {O}(NW/K+W^{2})$, and the disk complexity is $\mathcal {O}(NW)$.

We can consider a more elaborate computational procedure to reduce the necessary disk space from $\mathcal {O}(NW)$ to $\mathcal {O}(N+KW^{2})$. Briefly, we retain $d{\alpha ^{h}_{k}}$ only for 0≤*h*≤*W* and *e*−*W*≤*k*≤*e* on the disk for each node. They are used to reconstruct *Δα* only in the range *e*−*W*≤*k*≤*e*. They are then used to compute *Δα* for the entire segment in the second round of DP computation, taking $\mathcal {O}(NW^{2}/K)$ time.

ParasoR is written in the C++11 language, and it uses a portion of the source code of the ViennaRNA package [38] for energy parameters and Centroid fold for visualization [31]. The ParasoR source code and a detailed manual are available at https://github.com/carushi/ParasoR/.

### Validation of our implementation

To validate our algorithm and implementation, we used the RNAplfold program (ViennaRNA package v2.0.7) with its sliding-window feature turned off. Although RNAplfold is mainly designed for the averaging sliding-window method, it computes the probabilities of the global folding model with the maximal span constraint when its window size parameter is set to the input sequence length. Additional file 1: Figure S9 shows the consistency of stem probabilities between ParasoR and RNAplfold up to around 3,000 bases. When input sequences are longer than 3,000 bases, the difference increases as RNAplfold returns invalid probabilities >1.0 due to numerical problems or overflow errors (Additional file 1: Figure S10). On the other hand, the probabilities of ParasoR are within the range [ 0,1] even when the length of input sequence is increased to 3G bases (Additional file 1: Figure S4). We also compared the stem probabilities of ParasoR calculated for human chromosome 1 and the probabilities of RNAplfold calculated for a gene within chromosome 1, and found that they are sufficiently close, which indicates that the accuracy of ParasoR does not degrade in the midst of long input sequences (Additional file 1: Figure S11). We also computed the probabilities with varying precisions of real numbers (i.e., we used 32-bit float, 64-bit double, and 128-bit long double types as variable declaration in the C++ program). While the 32-bit version returned different values (by ≈10^−2^) from those of the 128-bit version for a long sequence of length 10K bases, the difference between 64-bit and 128-bit was very small (≈10^−13^) (Additional file 1: Figure S13). Hence, we concluded that using 64-bit double is sufficient to avoid numerical problems. We compared the computational time of ParasoR with those of RNAplfold and LocalFold. Additional file 1: Table S5.2 shows that ParasoR is faster than LocalFold but it is about 80 times slower than RNAplfold. Presumably, this is because we use logsum() function for all the sum operation of Boltzmann factor, while RNAplfold uses the elementary plus operator for speed. We leave the optimization of computational time of the ParasoR software to future work. In Additional file 1: Figure S26 and Section 5.2.3, we compared the computation time of ParasoR with a different number of computer nodes, which shows that ParasoR can use multiple nodes efficiently to drastically reduce the computational time for realistic lengths (10k to 1 million bases) of human transcripts.

### ***k***-mer frequency linear regression

We designed a normalization method for genome-wide comparisons of stem probabilities using GC content and other, more complex features. Using Python 2.7 and the NumPy library, we implemented a linear regression using the average stem probability *p*_stem_(*i*) with a ridge penalty. A least-squares method is used to estimate a parameter vector *w* with regularization term *λ*, and its error function is formulated as below.

(4) $$\begin{array}{*{20}l} \frac{1}{2}\sum_{n=1}^{N}\left(y_{n}-w^{T}x_{n}\right)^{2}+\frac{\lambda}{2}w^{T}w \end{array} $$

In this formula, *λ* is a constant, and *w* is a parameter vector in the same dimension as *x*. This equation is differentiable with respect to *w*, and we obtain *w* to minimize this error function. In this paper, we calculated 4-mer composition (*#AAAA*, *#AAAC*, …) and average stem probability for each 32-mer fragment ($\bar {p}_{\text {stem}}$), then set *x*_*n*_ and *y*_*n*_ to $\left (\frac {1}{32}_, \frac {\text {\#AAAA}}{32}_, \frac {\text {\#AAAC}}{32}_, \ \cdots \ \frac {\text {\#UUUU}}{32}\right)$ and $\bar {p}_{\text {stem}}$, respectively.

The model was trained with the stem probabilities on both strands of the entire human genome. The maximal span W for computing stem probabilities is set to 200 for all the experiments in the main text. In the Additional file 1, we have shown a histogram of stem probabilities for W = 200 and W=1,000 (Additional file 1: Figure S7 Right). The correlation coefficient of stem probabilities between W =200 and W =1,000 is 0.713. The stem probability gradually increases with W as the number of possible base pairs increases. However, the accuracy of structure prediction is not much affected by the value of W. As shown in Additional file 1: Table S4.1 in Additional file 1: Section 4.2, a large maximal span (W=1,000) only slightly decreased the accuracy of prediction against PARS score dataset.

We subtracted the average probability predicted by regression from $\bar {p}_{\text {stem}}(i)$, which is denoted by $\Delta \bar {p}_{\text {stem}}(i)$. In Additional file 1: Figure S24, we show that this subtraction greatly reduces the correlation between neighboring 32-nt windows, ensuring independence between samples. As such, we used $\Delta \bar {p}_{\text {stem}}(i)$ of all non-overlapping 32-nt windows as independent degrees of freedom for hypothesis testing. We describe the details of this regression, such as selection of sequence features, parameter optimization, and the statistical independence of each normalized $\Delta \bar {p}_{\text {stem}}$, in Additional file 1: Section 5.1 and Additional file 1: Figure S21, S22, S23, S24, S25, S26, S27, S28, S29 and S30. Also, we re-implemented a few hypothesis testing algorithms (Additional file 1: Chapter 8) for cases in which popular statistical tools such as R cannot handle the necessarily large number of data points such as Figs. 5 and 6.

### Comparison with other tools

The accuracy of ParasoR in local secondary structure prediction was compared with that of RNALfold. However, since RNALfold predicts multiple overlapping structures, we extracted the longest structures in ascending order of free energies without any overlap, in accordance with the post-processing described in Ref. [26].

We also compared the stem probabilities of ParasoR with two average-sliding-window methods, RNAplfold (ViennaRNA package v2.0.7) and LocalFold (v1.0). According to the previous study [29], in which optimal parameter sets were investigated, we set the parameter as follows: a maximal span of pairing L to 150, average window size W to 200, and skip size b to 10 (only for LocalFold).

Since RNALfold has no appropriate parameters that control the balance between sensitivity and specificity, we used the MCC scores to evaluate the accuracy of ParasoR and RNALfold for prediction with one condition, $ MCC = \frac {TP\times TN -FN\times FN}{\sqrt {(TP+FP)(TP+FN)(TN+FP)(TN+FN)}} $, where TP, TN, FP, and FN correspond to the numbers of true positive, true negative, false positive, and false negative predictions, respectively. For validation of stem probabilities, we used the area under the receiver operating characteristic curve, which plots false positive rates ($\frac {FP}{FP+TN}$) for *x*-axis and true positive rates ($\frac {TP}{TP+FN}$) for *y*-axis with varying threshold scores.

### Datasets and data manipulation

We downloaded assemblies hg19 and GRCm38 of the reference human and mouse genomes, respectively, from the UCSC Genome Browser database [52]. We annotated the genomes using the output of RepeatMasker and the RefSeq genes [53], which represent 45,377 human and 33,988 mouse genes. Where there were overlapping annotations, we prioritized them according to the strength of their biases in base compositions (in the following descending order): *Repeat*, *CDS*, 3^′^-UTR, 5^′^-UTR, *Intron*, *Non-coding RNA exon*, *Non-coding RNA intron*, *Antisense*, and *Intergenic* regions. Here, Repeat annotations represent the sense and antisense strands of all the repeat elements reported by RepeatMasker containing retrotransposons, tandem repeats, and so forth. We carefully removed these repeat elements because our regression method does not take account of the mutual dependencies of 4-mer sequences in the same 32-mer window (Figures S1 and S2). For example, a simple repeat of Adenine (AAAAAAAAAAAA…) cannot bind to the other As in this window, but the linear regression may evaluate the component of AAAA to have a weak binding efficiency because AAAA in other contexts can form a stem. Actually, we have shown in Additional file 1: Figures S27 and S31 that low complexity elements contained in the Repeat category tend to have large $|\Delta \bar {p}_{\text {stem}}|$, that is, the prediction of stem probabilities by linear regression is systematically poor for such sequences. Since the repeat regions occupy a large part of non-coding regions, such bias will make the hypothesis testing extremely difficult. Therefore, our analyses only studied non-repetitive sequences.

**Fig. 11:**
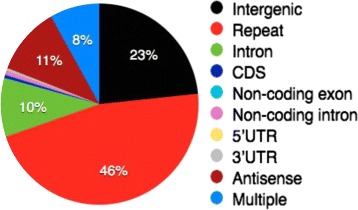
Fractions of 32-mers categorized by the genome annotations

The Antisense category contains all the antisense sequences of transcribed regions except Repeat and any transcribed sequences (Additional file 1: Figure S3). For computation of structural preferences (Figs. 5 and 6), we assigned one of the annotation categories to all the non-overlapping 32-mers. If a 32-mer contains a boundary of different annotations, we labeled the 32-mer with the “*Multiple*” annotation category. The fraction of each sequence annotation for the human genome is shown in Fig. 11. Thus, the Intergenic regions contain no Repeat regions, no sense or antisense strands of transcribed regions, and no sequences close to their boundaries. The total sample sizes used for hypothesis testing were as follows: Intergenic 41,764,604, Intron 17,004,161, CDS 843,820, 5^′^-UTR 86,026, and 3^′^-UTR 631,546 for the genomic sequence; Intron 34,544,536, CDS 1,469,721, 5^′^-UTR 201,267, and 3^′^-UTR 1,278,476 for pre-mRNA; CDS 1,772,283 5^′^-UTR 215,658 and 3^′^-UTR 1,279,508 for mRNA. Our annotation rule and any additional information such as the ratio of repetitive regions are detailed in Additional file 1: Section 2.2.

To compare ParasoR with high-throughput experimental structural analyses, we used a PARS dataset (GSM1226157 and GSM1226158, a renatured sample) [37]. Among human RefSeq genes, 33,603 were extracted as mappable mRNAs in the GM12878 sample. We compared the PARS scores with stem probabilities *p*_stem_(*i*) using the CG energy model [54] (see Additional file 1: Chapter 4 and Additional file 1: Figure S14, S15, S16, S17 and S18). The number of mapped reads was used to filter out inconclusive regions.

To compare the structural preferences of different genomic regions, we first computed stem probabilities for all genomic positions using chromosome sequences as input RNA sequences. As shown in Additional file 1: Figure S11, this roughly corresponds to computing the (unaveraged) stem probabilities for sequence windows of ∼2,000 bases in length. We also computed the stem probabilities for RefSeq pre-mRNAs and RefSeq mRNAs with the true boundaries. These probabilities were used for the calculation of the average stem probabilities $\bar {p}_{\text {stem}}(i)$ for non-overlapping 32-nt sequence windows. This length was chosen because the raw stem probabilities exhibit a bimodal distribution with peaks around 0 and 1, while the average stem probabilities exhibit a distribution close to normal when the averaging length is more than 32 bases (Additional file 1: Figure S7). The unimodality of $\bar {p}_{\text {stem}}(i)$ is important for the normalization of *k*-mer frequency bias below, as the linear regression requires unimodal objective variables for its high efficiency. Also, we expect that the distribution of average stem probabilities better represents local structural preferences than does the distribution of single-base stem probabilities. Even though a larger window size could also give a unimodal distribution, too large a window size leads to a highly peaked distribution around 0.5, in which no region-specific structural features will remain. A large window size also causes a reduction in the degrees of freedom for the hypothesis tests and thus reduces the significance of *p*-values (detailed in Additional file 1: Chapter 3).

To investigate structural changes around SSs after splicing, we computed the difference in stem probabilities between mRNA and pre-mRNA as *Δq*_stem_(*i*)=*p*_stem,mRNA_−*p*_stem,pre-mRNA_ for each site and each 32-mer sliding window in mRNA. For *Δq*_stem_(*i*) of each site, we then computed the median and median absolute deviation of *Δq*_stem_(*i*) values within a 200-nt window around each SS. We computed the correlations of them with gene expression levels, GC contents around SSs, and intron lengths. For gene expression levels, we used the CAGE promoter FANTOM5 expression data [55, 56]. We used average mRNA expression levels across all tissues and removed tissue-specific mRNAs that satisfy log10(median normalized expression) ≤0.5. To summarize GC content, we used the GC content of 200 bases around each SS in the mRNA, as well as the averaged GC contents for the 200-nt sequences around the donor and acceptor sites in the pre-mRNA.

In the gene set enrichment analysis, we ranked all SSs according to the median of *Δq*_stem_(*i*) for each SS, and the functional enrichment among the top 10 % of the most post-accessible or post-structural genes was analyzed using the DAVID web tool [47].

For genome-wide computation, we used a super computer system at the Human Genome Center (HGC, http://hgc.jp), which consists of Intel Xeon E7 8837, Intel Xeon X5675, and AMD Opteron 6276 CPUs and has a total memory of 2 TB. Average elapsed times and required memory sizes on HGC super computer for human chromosomes are shown in Additional file 1: Figure S25.

## Ethics approval and consent to participate

In this paper, we only applied public genome sequences and transcript annotation of human and mouse.

## Consent for publication

Not applicable.

## Availability of data and material

In this paper, the dataset supporting the conclusions of this article are available in (1) PARS score (GSM1226157 and GSM1226158) [37], (2) CisReg dataset [29], (3) RefSeq database [53], and (4) FANTOM dataset (hg19.cage_peak_counts_ann.osc.txt) [56]. Although the part of raw data underlying the conclusions of this article such as *p*_stem_ and *Δp*_stem_ is not available in an open access repository, please contact the corresponding author (RK) if there is interest. Our novel software, ParasoR, is freely available at https://github.com/carushi/ParasoR under the GNU GPL. ParasoR is written in the C++11, which can run on multi-platform. We tested ParasoR running in OS X 10.9 with Apple LLVM 6.0, and Cygwin environment with GCC4.6.

## Additional file

Additional file 1 This supplement provides details of ParasoR algorithm, manipulation of dataset, benchmarking, statistical testing, and so on. It also contains Supplementary figures cited in the main text. (PDF 4640 kb)

## Abbreviations

AS: Alternative splicing
AUC: Area under receiver operating characteristic curve
CDS: Coding sequence
DPg; MFE: Minimum free energy
MCC: Matthews correlation coefficient
PARS: Parallel analysis of RNA structure
SS: Splice site
UTR: Untranslated region
