# Supplementary material for: Parallel computation of genome-scale RNA secondary structure to detect structural constraints on human genome
Source: BMC Bioinformatics. 2016 May 6;17:203. doi: 10.1186/s12859-016-1067-9 (PMC4858847; doi:10.1186/s12859-016-1067-9)
Supplement: Additional file 1 — This supplement provides details of ParasoR algorithm, manipulation of dataset, benchmarking, statistical testing, and so on. It also contains Supplementary figures cited in the main text. (PDF 4640 kb) [file 12859_2016_1067_MOESM1_ESM.pdf]

Parallel computation of genome-scale RNA secondary structure to detect structural constraints on human genome

Risa Kawaguchi and Hisanori Kiryu

April 13, 2016

Contents

1 ParasoR algorithm **4**
- 1.1 Rfold grammar . . . 4
- 1.2 ParasoR algorithm . . . 6
- 1.3 Divide procedure . . . 7
- 1.4 Connect procedure . . . 8
- 1.5 Probability calculation procedure . . . 9

2 Manipulation of datasets and database construction **12**
- 2.1 Dataset of transcript sequences . . . 12
- 2.2 Annotation rule . . . 12
- 2.3 Shuffling of sequence . . . 13
- 2.4 Sequence logo construction . . . 13

3 Validation of ParasoR for long RNA **14**
- 3.1 Property of energy model and stem probability . . . 14
- 3.2 Testing the accuracy of ParasoR by comparison with RNAplfold . . . 16
- 3.3 Influence of flanking region . . . 16
- 3.4 Varying the precisions of floating point numbers . . . 17

4 Consistency of thermodynamic prediction methods and experimental structure analyses **18**
- 4.1 PARS score manipulation . . . 18
- 4.2 Concordance between ParasoR predictions and PARS data . . . 19
- 4.3 Concordance between ParasoR predictions and Rfam structures . . . 21

5 Genome-wide simulation by ParasoR **22**
- 5.1 Linear regression of stem probability with sequence composition statistics 22
- 5.2 Calculation time and memory . . . 24
  - 5.2.1 Human chromosome . . . 24

5.2.2 Comparison of calculation time with other tools 24
5.2.3 Random sequences for the simulation of pre-mRNA and intron computation 25
5.3 Stem probability distribution for each genomic region 25
5.4 Genomic features in structured and accessible regions after normalization 26

6 Structural preferences in mRNA and pre-mRNA 28
6.1 Structural preferences of human and mouse transcripts 28
6.2 Comparison of structural preferences with antisense and shuffled sequences 29
6.3 Stem probability profile around start codons, termination codons, and splice sites 29

7 Conformational change after splicing event 31
7.1 General tendency of conformational changes between mRNA and pre-mRNA 31
7.2 Cause of drastic conformational changes 32
7.3 Gene set enrichment analyses 32
7.4 Difference of structure propensity between sense and antisense sequence . 34

8 Procedures to compute p-values for some nonparametric tests 39
8.1 Wilcoxon's rank sum test 39
8.2 Wilcoxon's signed rank test 40
8.3 Conover's squared ranks test for testing equal variances between samples 41
8.4 Computing p-values from Z-scores 42

CHAPTER 1

ParasoR algorithm

ParasoR is a novel software application intended for local RNA secondary structure prediction on genome-scale data. ParasoR is primarily based on the Rfold grammar [1], which is based on the conventional energy models subject to the constraint of maximal distance between base pairs. In this chapter, we describe the Rfold grammar and detail the ParasoR algorithm for constructing databases of local folding energy changes used for structure predictions.

1.1 Rfold grammar

In the Rfold model, the transition rules are expressed as follows.

$$\begin{aligned}
\text{Outer} &\longrightarrow \epsilon|\text{Outer}\cdot\text{a}|\text{Outer}\cdot\text{Stem}\\
\text{Stem} &\longrightarrow \text{b}<\cdot\text{Stem}\cdot\text{b}>|\text{b}<\cdot\text{StemEnd}\cdot\text{b}>\\
\text{StemEnd} &\longrightarrow \text{s}_\text{n}|\text{s}_\text{m}\cdot\text{Stem}\cdot\text{s}_\text{n}\ (\text{m}+\text{n}>0)|\text{Multi}\\
\text{Multi} &\longrightarrow \text{a}\cdot\text{Multi}|\text{MultiBif}\\
\text{MultiBif} &\longrightarrow \text{Multi1}\cdot\text{Multi2}\\
\text{Multi1} &\longrightarrow \text{MultiBif}|\text{Multi2}\\
\text{Multi2} &\longrightarrow \text{Multi2}\cdot\text{a}|\text{Stem}
\end{aligned}$$

In these rules, ϵ represents the null terminal symbol; a, an unpaired nucleotide; s_k, an unpaired subsequence whose length is k; and $b<, b>$, a base pair. Rfold enables an enumeration of possible structure patterns following these transition rules. In addition, Rfold sums up the Boltzmann factor of energies of the partial structure that belongs to each state based on the inside-outside algorithm so that Rfold calculates the expected value of certain partial structure or transition based on the Boltzmann ensemble.

Each inside and outside variable is sequentially computed using a dynamic programming technique as follows.

$$\alpha_{\text{Stem}}(i,j) = \sum \begin{cases} \alpha_{\text{Stem}}(i+1,j-1)\cdot t(\text{Stem}\rightarrow\text{Stem}) \\ \alpha_{\text{StemEnd}}(i+1,j-1)\cdot t(\text{Stem}\rightarrow\text{StemEnd}) \end{cases}$$

$$\alpha_{\text{MultiBif}}(i,j) = \sum \begin{cases} \alpha_{\text{Multi1}}(i,k)\cdot\alpha_{\text{Multi2}}(k,j)\cdot t(\text{MultiBif}\rightarrow\text{Multi1}\cdot\text{Multi2}) \\ \text{for } i<k<j \end{cases}$$

$$\alpha_{\text{Multi2}}(i,j) = \sum \begin{cases} \alpha_{\text{Stem}}(i,j)\cdot t(\text{Multi2}\rightarrow\text{Stem}) \\ \alpha_{\text{Multi2}}(i+1,j-1)\cdot t(\text{Multi2}\rightarrow\text{Multi2}) \end{cases}$$

$$\alpha_{\text{Multi1}}(i,j) = \sum \begin{cases} \alpha_{\text{Multi2}}(i+1,j-1)\cdot t(\text{Multi1}\rightarrow\text{Multi2}) \\ \alpha_{\text{MultiBif}}(i,j)\cdot t(\text{Multi}\rightarrow\text{MultiBif}) \end{cases}$$

$$\alpha_{\text{StemEnd}}(i,j) = \sum \begin{cases} t(\text{StemEnd}\rightarrow(\text{Hairpin})) \\ \alpha_{\text{Stem}}(i',j')\cdot t(\text{StemEnd}\rightarrow(\text{Interior})\rightarrow\text{Stem}) \\ \text{for } i\le i'<j'\le j,\ 0<(j-j')+(i'-i)\le C \\ \alpha_{\text{Multi}}(i,j)\cdot t(\text{StemEnd}\rightarrow\text{Multi}) \end{cases}$$

$$\alpha_{\text{Outer}}(j) = \sum \begin{cases} 1 \text{ if } j=0 \\ \alpha_{\text{Outer}}(j-1)\cdot t(\text{Outer}\rightarrow\text{Outer}) \\ \alpha_{\text{Outer}}(k)\cdot\alpha_{\text{Stem}}(k,j)\cdot t(\text{Outer}\rightarrow\text{Outer}\cdot\text{Stem}) \\ \text{for } (j-W-1)\le k<j \end{cases}$$

$$\beta_{\text{Outer}}(j) = \sum \begin{cases} 1 \text{ if } j=N+1 \\ \beta_{\text{Outer}}(j+1)\cdot t'(\text{Outer}\rightarrow\text{Outer}) \\ \alpha_{\text{Stem}}(j,k)\cdot\beta_{\text{Outer}}(k)\cdot t'(\text{Outer}\rightarrow\text{Outer}\cdot\text{Stem}) \\ \text{for } j<k\le(j+W+1) \end{cases}$$

$$\beta_{\text{StemEnd}}(i,j) = \quad \beta_{\text{Stem}}(i-1,j+1)\cdot t'(\text{Stem}\rightarrow\text{StemEnd})$$

$$\beta_{\text{Multi}}(i,j) = \sum \begin{cases} \beta_{\text{StemEnd}}(i,j)\cdot t'(\text{StemEnd}\rightarrow\text{Multi}) \\ \beta_{\text{Multi}}(i-1,j)\cdot t'(\text{Multi}\rightarrow\text{Multi}) \end{cases}$$

$$\beta_{\text{Multi1}}(i,j) = \sum \begin{cases} \beta_{\text{MultiBif}}(i,k)\cdot\alpha_{\text{Multi2}}(j,k)\cdot t'(\text{MultiBif}\rightarrow\text{Multi1}\cdot\text{Multi2}) \\ \text{for } j<k\le(i+W+1) \end{cases}$$

$$\beta_{\text{Multi2}}(i,j) = \sum \begin{cases} \beta_{\text{Multi2}}(i,j+1)\cdot t'(\text{Multi2}\rightarrow\text{Multi2}) \\ \beta_{\text{Multi1}}(i,j)\cdot t'(\text{Multi1}\rightarrow\text{Multi2}) \\ \beta_{\text{MultiBif}}(k,j)\cdot\alpha_{\text{Multi1}}(k,i)\cdot t'(\text{MultiBif}\rightarrow\text{Multi1}\cdot\text{Multi2}) \\ \text{for } (j-W-1\le k<i) \end{cases}$$

$$\beta_{\text{MultiBif}}(i,j) = \sum \begin{cases} \beta_{\text{Multi1}}(i,j)\cdot t'(\text{Multi1}\rightarrow\text{MultiBif}) \\ \beta_{\text{Multi}}(i,j)\cdot t'(\text{Multi}\rightarrow\text{MultiBif}) \end{cases}$$

$$\beta_{\text{Stem}}(i,j) = \sum \begin{cases} \alpha_{\text{Outer}}(i)\cdot\beta_{\text{Outer}}(j)\cdot t'(\text{Outer}\rightarrow\text{Outer}\cdot\text{Stem}) \\ \beta_{\text{StemEnd}}(i',j')\cdot t'(\text{StemEnd}\rightarrow(\text{Interior})\rightarrow\text{Stem}) \\ \text{for } i'\le i<j\le j', 0<(i-i')+(j'-j)\le C \\ \beta_{\text{Multi2}}(i,j)\cdot t'(\text{Multi2}\rightarrow\text{Stem}) \\ \beta_{\text{Stem}}(i-1,j+1)\cdot t'(\text{Stem}\rightarrow\text{Stem}) \end{cases}$$

The expected values in our study such as stem probability are estimated as the sum of the state transition probabilities $p(\sigma, k, l \to \sigma', k', l')$.

$$p(\sigma, k, l \to \sigma', k', l') = \sum_{\zeta \in \Omega(\sigma,k,l \to \sigma',k',l')} e^{dG(\zeta,x)/RT}/Z \tag{1.1}$$

$$= \beta_\sigma(k, l)t(\sigma, k, l \to \sigma', k', l')\alpha_{\sigma'}(k', l')/Z \tag{1.2}$$

$$Z = \sum_{\zeta \in \Omega_0} e^{dG(\zeta,x)/RT} = \alpha_{\text{Outer}}(N) = \beta_{\text{Outer}}(0) \tag{1.3}$$

where σ and σ' represent nonterminal symbols of the Rfold grammar; k, l, k', and l' represent sequence positions; ζ, a secondary structure; $\Omega(\sigma, k, l \to \sigma', k', l')$, the set of secondary structures containing the transition $(\sigma, k, l \to \sigma', k', l')$; $dG(\zeta, x)$, the free energy for sequence x with structure ζ; R, the gas constant; T, the absolute temperature; Z, the partition function; $\beta_\sigma(k, l)$, the outside variables; $\alpha_{\sigma'}(k', l')$, the inside variables; $t(\sigma, k, l \to \sigma', k', l')$, the Boltzmann factor for the transition; and Ω_0, the set of all the secondary structures of sequence x. Summation of the state transition probability following this equation permits the computation of several measures of structure such as base pairing probability, accessibility, and structural profiles [2, 3].

For long sequences such as mRNA and pre-mRNA, however, the increased number of possible structures makes it almost impossible to calculate Z and β directly even though the increase of α is restricted by the maximal span.

1.2 ParasoR algorithm

ParasoR is a parallel extension of the Rfold algorithm intended for the prediction of genome-scale sequences, and it is accomplished via three procedures: *Divide*, *Connect*, and *Probability calculation* procedure. One of the strongest features in ParasoR is its construction of databases of α_{Outer} and β_{Outer} in the form of their fold changes such as $d\alpha$, $d\beta$, $\Delta\alpha$, and $\Delta\beta$; each element is obtained by comparison with adjacent elements. The algorithm evaluates local increase rations of α_{Outer} and β_{Outer} in logarithmic scale, $\alpha(i) = \log(\alpha_{\text{Outer}}(i))$ and $\beta(i) = \log(\beta_{\text{Outer}}(i))$. Because a logarithmic transformation is applied to all internal variables of ParasoR in order to reduce numerical errors, we use $\alpha(i)$ and $\beta(i)$ hereafter to explain ParasoR algorithm.

When K computational nodes are available to calculate the secondary structure of a sequence of length N and maximal span W, ParasoR requires $\mathcal{O}(NW^2/K)$ computational time in the Divide procedure, $\mathcal{O}(NW)$ or $\mathcal{O}(KW^2 + NW^2/K)$ in the Connect procedure, and $\mathcal{O}(NW^2/K)$ in the Probability calculation procedure. In the Divide procedure, ParasoR calculates partial $d\alpha$ and $d\beta$ at each computational node independently. Then, in the Connect procedure, ParasoR establishes a database of $\Delta\alpha$ and $\Delta\beta$ from the stored $d\alpha$ and $d\beta$. $\Delta\alpha$ and $\Delta\beta$ are independent of how the input sequences is divided into subsequences. Finally, several expected values such as base pairing probability are computed for any queried region using the $\Delta\alpha$ and $\Delta\beta$ database.

In the following sections, we describe how to calculate $d\alpha$, $d\beta$, $\Delta\alpha$, and $\Delta\beta$. Then, we derive the formula of the "local partition function", which is the ratio of DP variables

and partition function, as a function of $\Delta\alpha$, $\Delta\beta$, and other variables whose magnitudes are bounded independently of N. This formula allows us to calculate the expectation values without the direct computation of Z and β whose magnitudes grow exponentially with N.

1.3 Divide procedure

To explain the methodology of the Divide procedure, we focus on the calculation of $d\alpha$ and $d\beta$ for the subsequence from the sth to eth positions after its assignment to a single node in the Divide procedure. First, we describe how to calculate $d\alpha$ using v, which is defined as follows to absorb all required energy during the transition between the Stem and Outer state.

$$v_{i,j,h} := \begin{cases} -\infty & \text{if } i < (s+1) \text{ and } i \neq (s-h) \\ \log(t(\text{Outer} \to \text{Outer})) & \text{else if } j = i+1 \\ \log\left(\alpha_{\text{Stem}}(i,j) \cdot t(\text{Outer} \to \text{Outer} \cdot \text{Stem})\right) & \text{otherwise} \end{cases} \tag{1.4}$$

Then, α is simply expressed using v.

$$\begin{aligned} \alpha(i) &= \log \alpha_{\text{Outer}}(i) & (1.5)\\ &= \log\left(\sum_{k=i-W}^{i} \exp(\alpha(k) + v_{k,i,i-k})\right) & (1.6)\\ &= \log\left(\sum_{h=0}^{W} \exp(\alpha(s-h) + \alpha_{i,h})\right) & (1.7) \end{aligned}$$

where $\alpha_{i,h}$ is a partial summation of α and recursively obtained as below.

$$\alpha_{i,h} := \begin{cases} 0 & \text{if } i < (s+1) \\ \log\left(\sum_{k=1}^{W} \exp(\alpha_{i-k,h} + v_{i-k,i,h})\right) & \text{else} \end{cases} \tag{1.8}$$

In Eq. 1.7, $\alpha_{i,h}$ is calculated from the data available in the same computational node while $\alpha(s-h)$ depends on results of the previous node. To cancel out the contribution of $\alpha(s-h)$, we consider $d\alpha_{i,h}$, or the local differences of α between two adjacent values.

$$\begin{aligned} d\alpha_{i,h} &:= \alpha_{i,h} - \alpha_{i-1,0} & (1.9)\\ &= \begin{cases} 0 & \text{if } i < (s+1) \\ \log \sum_{k=1}^{W} \exp\left(d\alpha_{i-k,h} + v_{i-k,i,h} - \sum_{j=i-k}^{i-1} d\alpha_{j,0}\right) & \text{if } i \geq (s+1) \end{cases} & (1.10) \end{aligned}$$

In this way, ParasoR computes $d\alpha_{k,h}$ and constructs a partial database of $d\alpha_{k,h}$ using a single node in $\mathcal{O}(NW^2/K)$ computational time.

In a similar way, $d\beta$ is also calculated by defining another v whose boundary condition is different from the previous v.

$$v_{i,j,h} := \begin{cases} -\infty & \text{if } e < j \text{ and } j \neq (e+h) \\ \log(t(\text{Outer} \to \text{Outer})) & \text{else if } j = i+1 \\ \log(\alpha_{\text{Stem}}(i,j) \ \cdot t(\text{Outer} \to \text{Outer} \cdot \text{Stem})) & \text{otherwise} \end{cases} \tag{1.11}$$

$$\beta(i) = \log \beta_{\text{Outer}}(i) \tag{1.12}$$

$$= \log \left(\sum_{k=i+W}^{i} \exp(\beta(k) + v_{i,k,i-k}) \right) \tag{1.13}$$

$$= \log \left(\sum_{h=0}^{W} \exp(\beta(e+h) + \beta_{i,h}) \right) \tag{1.14}$$

$$\beta_{i,h} := \begin{cases} 0 & \text{if } e < i \\ \log \left(\sum_{k=1}^{W} \exp(\beta_{i+k,h} + v_{i,i+h,h}) \right) & \text{else} \end{cases} \tag{1.15}$$

$$d\beta_{i,h} := \beta_{i,h} - \beta_{i+1,0} \tag{1.16}$$

$$= \begin{cases} 0 \\ \log \sum_{k=1}^{W} \exp \left(d\beta_{i+k,h} + v_{i,i+k,h} - \sum_{j=i+1}^{i+k} d\beta_{j,0} \right) \end{cases} \tag{1.17}$$

1.4 Connect procedure

The variables $d\alpha$ and $d\beta$ in the previous section contain the dependency on the fragmentation pattern. In the Connect procedure, ParasoR integrates these variables to calculate unique local fold changes $\Delta\alpha$ and $\Delta\beta$ for each position as follows.

$$\Delta\alpha_j := \alpha_j - \alpha_{j-1} \tag{1.18}$$

$$= \log \left(\frac{\sum_{h=0}^{W} \exp(d\alpha_{j,h} + d\alpha_{j-1,0} - \sum_{i=s-h+1}^{s} \Delta\alpha_i)}{\sum_{h=0}^{W} \exp(d\alpha_{j-1,h} - \sum_{i=s-h+1}^{s} \Delta\alpha_i)} \right) \tag{1.19}$$

$$\Delta\beta_j := \beta_j - \beta_{j+1} \tag{1.20}$$

$$= \log \left(\frac{\sum_{h=0}^{W} \exp(d\beta_{j,h} + d\beta_{j+1,0} - \sum_{i=e}^{e+h} \Delta\beta_i)}{\sum_{h=0}^{W} \exp(d\beta_{j+1,h} - \sum_{i=e}^{e+h} \Delta\beta_i)} \right) \tag{1.21}$$

where $\Delta\alpha$ and $\Delta\beta$ are computed by the sum of Boltzmann factors of the free energy of the secondary structures inferred within the subsequences of length $W\cdot$ Constant or less. At the same time, the calculation of these values indirectly includes the influences of long flanking regions (discussed in Chap. 3).

We implemented the Connect procedure in two different ways. In the first way, ParasoR stores the whole matrix of $d\alpha$ and $d\beta$ in the Divide procedure and sequentially

Algorithm 1 Memory-saving algorithm in the Divide and Connect procedures

1: Compute $d\alpha_k^h$ $(s \leq k \leq e)$ and $d\beta_k^h$ $(s \leq k \leq e)$ in multiple nodes
2: Store only $d\alpha_k^h$ $(e - W \leq k \leq e)$ and $d\beta_k^h$ $(s \leq k \leq s + W)$ in the disk
3: Compute and save $\Delta\alpha(k)$ $(e - W \leq k \leq e)$ and $\Delta\beta(k)$ $(s \leq k \leq s + W)$ following Eq.1.21 for all the divided segments using a single node
4: Compute $d\alpha_k^h$ $(s \leq k \leq e)$ and $d\beta_k^h$ $(s \leq k \leq e)$ in multiple nodes again
5: Compute and save $\Delta\alpha$ and $\Delta\beta$ for all sequence positions using Eq. 1.21 in multiple nodes using the partial $\Delta\alpha$ and $\Delta\beta$ saved previously

computes $\Delta\alpha$ and $\Delta\beta$ in a single node. This implementation enables the removal of redundant calculations at the expense of reducing the size of external files. This procedure requires $\mathcal{O}(NW)$ computational time to establish databases for $\Delta\alpha$ and $\Delta\beta$ of file size $\mathcal{O}(N)$ using $d\alpha$ and $d\beta$ which need a temporal storage file of $\mathcal{O}(NW^2)$. This temporal storage becomes, however, very large, up to around 10 TB for the human genome sequence in `double` (8-byte) precision. Accordingly, ParasoR also uses another implementation that constructs a part of $d\alpha$ and $d\beta$ to compute a vector of $\Delta\alpha$ and $\Delta\beta$ of length only $(W + 1)$ for the ends of the assigned sequence from the saved $d\alpha$ and $d\beta$. Then, each node recalculates $d\alpha$ and $d\beta$ and completes the databases of $\Delta\alpha$ and $\Delta\beta$ for the assigned region using the partial vector of $\Delta\alpha$ and $\Delta\beta$. A pseudo code for this algorithm is given (Algorithm 1). This procedure only requires the temporary storage for $d\alpha_k^h$ $(e - W \leq k \leq e)$ and $d\beta_k^h$ $(s \leq k \leq s + W)$ of size $\mathcal{O}(KW^2)$. The time complexity of the first and fourth steps is $\mathcal{O}(NW^2/K)$ for each node. The time complexity of the third step is $\mathcal{O}(KW^2)$ for a single node. The time complexity of the last step is $\mathcal{O}(NW/K)$ for each node.

1.5 Probability calculation procedure

For whole genome sequences, a partition function Z becomes so large that we cannot compute Z directly. However, Z is required in the form of β/Z in the probability formula, and the increases of β and Z are considered to balance each other because a large number of possible structures would increase both of them. In this section, we show that $r = Z/\beta$ is evaluated only by local variables such as $\Delta\alpha$ and $\Delta\beta$, which are restricted to increase only up to the constant value depending on the maximal span rather than the whole sequence length.

We characterize the set of the "local" structures by locally maximal, non-overlapping substructures enclosed by a base pair. The pair of bases that encloses each local structure of subsequence $x_{i,j}$ between i and j is referred to as the outermost pair. For the explanation of ParasoR algorithm, we define the set of outermost pairs as $P = \{(i, j) : (x_{i+1}, x_j)$ is one of the canonical base pairs and $5 \leq j - i \leq W\}$ and the set $S(i')$ as $\{(i, j) \in P \mid i \leq i' < j\} \bigcup \{(i', i' + 1)\}$ for any position i'. The sequence length of the local structures ($|j - i|$) must not exceed W in ParasoR algorithm because base pairs between more distant bases are not allowed by the constraint of maximal

span. Thus, all possible structures should consist of non-overlapping local structures and fragments of exterior loops between them. In Equation 1.3, the magnitudes of α and t do not change with N since they are computed from the subsequence $x_{k,l}$ whose length does not exceed W. Z and β are, however, the sums of Boltzmann factors for the subsequences of length $\mathcal{O}(N)$ and grow exponentially with N. On the other hand, the probability $p(\sigma, k, l \to \sigma', k', l')$ should be between 0.0 and 1.0, and so a large cancellation between $\beta_\sigma(k, l)$ and Z must occur, which reduces the numerical precision. The cancellation is assured because the contributions from the structures far outside of (k, l) are almost the same. This can be seen from the following decompositions.

$$\beta_\sigma(k,l) = \sum_{(i,j)\in P,\ i\le k<l\le j} \alpha_{\text{Outer}}(i)\beta_\sigma(k,l;i,j)\beta_{\text{Outer}}(j) \tag{1.22}$$

$$Z = \alpha_{\text{Outer}}(i')\beta_{\text{Outer}}(i'+1)t(\text{Outer} \to \text{Outer}) + \sum_{(p,q)\in P,\ p\le i'<q} \alpha_{\text{Outer}}(p)t(\text{Outer}, k', l' \to \text{Outer}\cdot\text{Stem}, p, q)\alpha_{\text{Stem}}(p,q)\beta_{\text{Outer}}(q) \tag{1.23}$$

$$= \sum_{(p,q)\in S(i')} \alpha_{\text{Outer}}(p)u(p,q)\beta_{\text{Outer}}(q) \tag{1.24}$$

$$u(p,q) = \begin{cases} t(\text{Outer} \to \text{Outer} & \text{if } p+1=q \\ t(\text{Outer}, p, q \to \text{Outer}\cdot\text{Stem}, p, q)\alpha_{\text{Stem}}(p,q) & \text{otherwise} \end{cases}$$

Here, i' can be set to any position and $\beta_\sigma(k, l; i, j)$ are the outside variables for the subsequence between the outermost pair (i, j) satisfying the initial condition $\beta_{\text{Stem}}(i, j; i, j) = t(\text{Outer}, i, j \to \text{Outer}\cdot\text{Stem}, i, j)$. Equation 1.22 follows because the outside variables are the sum of the contributions of all possible patterns of outermost pairs. Equation 1.23 also follows because a base at any position i' ($x_{i'+1}$) is either within the outermost pair (p, q) or is an exterior base.

Then we define a local partition function $r(i, j)$ for the computation of expected values of local structures on subsequence $x_{i+1,j}$ as below.

$$r(i,j) := \log\left(\frac{Z}{\alpha_{\text{Outer}}(i)\beta_{\text{Outer}}(j)}\right) \tag{1.25}$$

$$= \log\left(\frac{\sum_{p,q\in S(i)} \alpha_{\text{Outer}}(p)u(p,q)\beta_{\text{Outer}}(q)}{\alpha_{\text{Outer}}(i)\beta_{\text{Outer}}(j)}\right)$$

$$= \log\left(\sum_{p,q}\left\{\frac{\alpha_{\text{Outer}}(p)}{\alpha_{\text{Outer}}(i)}\ u(p,q)\ \frac{\beta_{\text{Outer}}(q)/\beta_{\text{Outer}}(i)}{\beta_{\text{Outer}}(j)/\beta_{\text{Outer}}(i)}\right\}\right) \tag{1.26}$$

$$= \log\left(\sum_{p,q}\left\{\prod_{h=p}^{i-1}\exp(\Delta\alpha_h)\right\}\ u(p,q)\ \left\{\prod_{h=i}^{q-1}\exp(\Delta\beta_h)\right\} / \left\{\prod_{h=i}^{j-1}\Delta\exp(\beta_h)\right\}\right) \tag{1.27}$$

In this chapter, we have already shown the DP algorithm that directly computes $\Delta\alpha$ and $\Delta\beta$ without recourse to α_{Outer} and β_{Outer}. The inner variables $u(p,q)$ can be computed without numerical difficulties by using the ordinary inside algorithm.

In this manner, we can avoid the computation of variables that exponentially increase with N for $r(i,j)$. Using $r(i,j)$, a fold change $\beta_\sigma(k,l)/Z$ is replaced by the outside variable for local structures and local partition function as below.

$$\beta_\sigma(k,l)/Z = \sum_{(i,j)\in P,\ i\le k<l\le j} \beta_\sigma(k,l;i,j)/\exp(r(i,j))$$

In the ParasoR software, it computes $r(x,x)$ for the start position x first, then uses it to calculate r for the outermost pair (i,j) shown as below.

$$r(x,x) := \log\left(\frac{Z}{\alpha_{\text{Outer}}(x)\beta_{\text{Outer}}(x)}\right) \tag{1.28}$$

$$= \log\left(\sum_{(i,j)\in S(x)} \exp\left(-\sum_{k=i+1}^{x}\Delta\alpha_k + \log(u(i,j)) - \sum_{k=x}^{j-1}\Delta\beta_k\right)\right) \tag{1.29}$$

$$r(i,j) = \log\left(\frac{Z}{\alpha_{\text{Outer}}(i)\beta_{\text{Outer}}(j)}\right) \tag{1.30}$$

$$= r(x,x) - \sum_{k=i+1}^{x}\Delta\alpha_k - \sum_{k=x}^{j-1}\Delta\beta_k \tag{1.31}$$

We should consider at most W^2 patterns of i and j for r computation and less than $W+1$ additions of energies that depend on subsequences shorter than $2W$. Also, an addition of $\Delta\alpha$ and $\Delta\beta$ should be executed fewer than W times. Therefore, in this way, we can compute an expected value only by variables whose absolute value has an upper bound depending on W.

For the next position $x+1$, the calculation of $r(x+1,x+1)$ from $r(x,x)$ takes $\mathcal{O}(1)$ time because the following equation holds.

$$r(x+1,x+1) = r(x,x) - \Delta\alpha_{x+1} + \Delta\beta_x \tag{1.32}$$

Because both a number of these summations and the length of required subsequences are not directly dependent on length N, ParasoR is able to compute the expected values using only "local" variables whose magnitudes are independent of N. We also discuss the numerical precision of computed variables such as $\Delta\alpha$, $\Delta\beta$, and expected variables for actual data in Chapter 3.

CHAPTER 2

Manipulation of datasets and database construction

2.1 Dataset of transcript sequences

RefSeq annotation data mapped to the hg19 (human) and GRCm38 (mouse) genomes were used in the transcript database construction as a source for mRNA and pre-mRNA sequences. As a result, we obtained 45,377 pre-matured RNAs which include non-coding RNAs for human and 33,988 for mouse. They mapped to the same region on the genome sequence as many as 19 times. Prior to structure prediction, we concatenated each sequence and inserted W ambiguous characters "N" between each transcript to avoid interactions between neighboring RNAs.

2.2 Annotation rule

To examine disparities in estimates of structure preference among types of genomic regions, we annotated genome sequences according to several functional annotation categories, which were prioritized according to the strength of their sequence constraint. First, we tagged gene regions on chromosomes according to RefSeq data for both coding RNA (as CDS, 5′-UTR, 3′-UTR, and introns, in descending order of importance) and non-coding RNA (as exons and introns) (Figure S1). These annotated groups contain repetitive elements in different ratios (Figure S2) and these proportions of repeat sequence probably have a close relationship with the function of each category. Our regression, however, specifically cannot normalize repeat regions enough as a total effect of each k-mer can be expressed by a simple summation. As such, we excluded repetitive regions from other annotation groups. We describe the important detail about the property of repetitive elements in Chapter 5. In addition, the antisense strand of the transcribed regions was removed from the intergenic regions and classified as "Antisense" group because of the possible strict constraints derived from the coding genes in

the sense strand. Although the antisense sequence of a transcribed gene may be possibly mapped to another transcribed gene, the majority of such regions were classified into the antisense group (Figure S3). The analysis of average stem probabilities for each 32-mer may have suffered from the influence of multiple annotations around the boundary regions. To remove such ambiguous effects, fragments that overlap with multiple annotations were classified into "Multiple" annotation group.

2.3 Shuffling of sequence

To investigate the structural preferences of genome sequences, randomly shuffled sequences were used for comparison. The codon composition ratio is a conserved factor among transcripts. Thus, 3-mer shuffling using the ushuffle module (http://digital.cs.usu.edu/~mjiang/ushuffle/) was applied to produce random transcripts for each concatenated mRNA and pre-mRNA sequence. At the same time, we divided whole sequences into each block with ambiguous characters inserted as a boundary between each transcript or found in pre-mRNA as an ambiguous region. For genome analyses, chromosome sequences are too long for the memory usage requirements of ushuffle. Accordingly, we randomly shuffled chromosome 1 for each individual sequence block in the resolution of single nucleotide, again divided by strings of the ambiguous character "N".

2.4 Sequence logo construction

To construct sequence logos in Figures S35, the RWebLogo package (http://cran.r-project.org/web/packages/RWebLogo/index.html) was applied to partial sequences around motif sites. For sequences around splice sites, however, the original sequence set was too large to apply RWebLogo. Hence, we implemented seqLoGo (https://github.com/carushi/seqLoGo) in Go language, which allows compressing all sequences to a small number of sequences while still expressing the same information with an accuracy rate that depends on the number of sequences retained after compression.

CHAPTER 3

Validation of ParasoR for long RNA

While a method of predicting RNA secondary structure has already been widely developed, to the best of our knowledge, there has not yet been a study that has examined structural properties of genome-scale data with various parameters. In this chapter, we examined the congruence of our results under a variety of conditions. Moreover, we analyzed properties of the energy model under the maximal span constraint.

3.1 Property of energy model and stem probability

The possible patterns of RNA secondary structure generally multiply in accordance with the sequence length N. A minimum free energy (MFE), which is one of the measures for evaluating the structure stability of RNA, also decreases with N. Although MFE can be normalized by dividing it by N to compare MFE between RNA sequences of different lengths, a previous investigation has indicated the risk of over normalization by division by N [4]. Because there is insufficient investigation of energy increase such as $\Delta\alpha$, we computed the distributions of $\Delta\alpha$ and $\Delta\beta$ using concatenated transcript sequences with the maximal span $W = 200, 400, and 1, 000$. We randomly sampled one $\Delta\alpha$ per 1,000 nt and Figure S4 shows that most of the $\Delta\alpha$ distribution was within a range from 0 to 1.6 even though the variance estimates increase with W. Although the length of transcript sequences without ambiguous bases is short, the result of human chromosome 1 sequence was also consistent with that of transcripts sequences.

In addition, because each local structure contributes to the increase of $\Delta\alpha$ and $\Delta\beta$ equally, the increase of $\Delta\beta$ is intrinsically comparable to $\Delta\alpha$ based on existing energy models. In this way we showed that $\Delta\alpha$ and $\Delta\beta$ calculated in ParasoR are small enough to calculate accurate stem probabilities while avoiding numerical errors even for genome sequences. On the other hand, because a partition function Z is calculated by summation of $\Delta\alpha$, Z is expected to be too large value for large N since it can be estimated by

$\exp(\overline{\Delta\alpha} \times N)$, where $\overline{\Delta\alpha}$ represents an expected value of $\Delta\alpha$.

The MFE is widely used to evaluate structure stability and extract structural preference of the sequence. However, this measure reflects only the best stabilized structure while ignoring numerous other structures [7]. Since the main purpose of ParasoR is the analysis of genome-scale RNA sequences, which decreases the quality of MFE estimates owing to an excessive number of possible structures. As such, we used the stem probability $p_{\text{stem}}(i)$ as a measure to evaluate local structural preferences. The stem probability is another typical index that expresses the tendency of structure preferences at a single-nucleotide resolution, and it has the advantage of being able to consider the stability of base pairing among an enormous variety of structures. To determine the general tendency of stem probability $p_{\text{stem}}(i)$, we generated 1,000 random sequences of 1,000 nt in length with a 50% GC content and averaged $p_{\text{stem}}(i)$ for each sequence without limiting the maximal span, in which $p_{\text{stem}}(i)$ is consistent with that of RNAfold (data not shown). In this analysis, $p_{stem}(i)$ was practically distributed around 0.6 with a notable fall at both ends of sequences, indicating that artificial sequence boundaries potentially cause such bias (Figure S5).

We also calculated a conditional base pairing probability p_{Cbpp} for each base pair to show that such a tendency is produced by the specificity of pairing with other sequence tips. Here, p_{Cbpp} of the jth position among base pairs with the ith base is defined as follows.

$$p^{i}_{\text{Cbpp}}(j|i) := p_{\text{Bpp}}(i,j)/p_{\text{Bpp}}(i) \qquad (3.1)$$

where $p_{\text{Bpp}}(i,j)$ is the pairing probability between the ith and jth bases, and $p_{\text{Bpp}}(i)$ is a marginalized base pairing probability for the ith base , which is equal to $p_{stem}(i)$. Hence, p_{Cbpp} is comparable to a likelihood of being partner with ith base. Figure S6 shows an example of p_{Cbpp} profiles for the 461th and 11th bases. While a profile of p_{Cbpp} for a nonterminal base indicates structural preferences for pairing within neighborhoods, such a profile for ends clearly shows that they are likely to form base pairs with their opposite ends.

This result raises an issue about sliding-window methods because it can reduce the calculation time but generate many biases at the ends of each window although the global folding algorithm such as ParasoR may cause them only at the ends of sequences.

Next, we simulated a distribution of stem probabilities p_{stem} and average stem probability $\bar{p}_{\text{stem}}$ for genome sequence data. As shown in Figure S7(L), the distribution of p_{stem} is bimodal at 0 and 1 and becomes nearly unimodal by window averaging after the structure prediction. In particular, the distribution of $\bar{p}_{\text{stem}}$ for each 32-mer or longer unit is almost unimodal. We also investigated the influence of different energy parameters on the distribution of p_{stem}. In Figure S7(C), although both of models show a bimodal distribution with the peak around 0 and 1, the strength of bimodality is different for each energy model. In particular, the Turner (2004) model is shown to have produced more strongly bimodal results than did the Andronescu (2007) model. This is likely because their energy parameters were estimated from different knowledge of secondary structures and thus reflects properties of the datasets. The influence of different maximal span sizes was also tested for various window sizes using structure-prediction data

from human chromosome 1. To evaluate an agreement between different conditions, a correlation coefficient was computed for several window sizes ranging from 10 to 400 nt. A comparison between $\bar{p}_{\text{stem}}$ from Turner (2004) and Andronescu (2007) was higher than 0.8, at least for any selected window size (Figure S8). Even accessibility, another typical index for structure evaluation, showed a high correlation with $\bar{p}_{\text{stem}}$. We also compared $\bar{p}_{\text{stem}}$ with $W = 200$ and that of three other maximal spans: 100, 150, and 400. All of them show the correlation coefficient higher than 0.8 for the window size larger than 32-mer (Figure S8).

For subsequent comparisons among different regions, a proper normalization according to sequence compositions is required. As such, we applied a linear regression method in which a unimodal distribution of $\bar{p}_{\text{stem}}$ is suitable. Although a larger window size produces stronger unimodality, structural preferences specific to each region were also averaged to the mean value. Based on these results, we selected the 32-mer window size in order to produce a concordant unimodal stem probability distribution without excess standardization.

3.2 Testing the accuracy of ParasoR by comparison with RNAplfold

To evaluate the robustness of ParasoR against overflow problems, we compared stem probabilities calculated with ParasoR and RNAplfold, which calculates the same values theoretically when the maximum window size and maximal span of base pairing are set to W. For comparison, we generated random sequences of 1,000, 3,000, and 5,000 nt in length, each with a 50% GC content. Figure S9 shows boxplots of stem probability differences between ParasoR and RNAplfold (ViennaRNA package v2.0.7) for each sequence with varying the maximal span W. There is little difference between these methods among 1,000 nt sequences. However, critical differences appeared for sequences with lengths of 3,000 and 5,000 nt. This discrepancy is due to an overflow of RNAplfold that results in a computed probability exceeding 1, while ParasoR can still produce an appropriate distribution in the range from 0 to 1 for much longer sequences such as genome sequences (Figure S10).

3.3 Influence of flanking region

We examined a way to take account of the structural influence of flanking sequences around target sites. To visualize the remodeling of the secondary structure by flanking sequences, the *PTGFR* gene (NM_001039585) was chosen as a subject sequence because it is sufficiently long ($> 49,000$ nt) and located at chr1:78,956,727-79,006,386, which is not the edge of a sequence block. Using RNAplfold, $p_{\text{stem}}(i)$ was calculated for a 200 nt center region of subsequences that contains a center region and varied lengths of flanking regions at both sides within the range at which no severe overflow problems occur in Sec. 3.2. As shown in Figure S11, differences of two stem probabilities converge to 0

as longer flanking regions are added to the RNAplfold computation. This demonstrates that the computed stem probabilities converge when flanking sequences at both ends exceed $2W$ bases, which is consistent with previous findings in Ref. [1, 3]. Thus, the stem probabilities computed for the entire chromosome roughly correspond to those for sliding windows with large ($\sim 4W$) margins. ParasoR simulated RNA secondary structure for the whole chromosome sequence while RNAplfold computed stem probability with a part of flanking sequence. The x-axis represents the length of flanking sequences around the region applied for the computation of RNAplfold.

We also calculated the influence of flanking region from an opposite viewpoint by appending additional sequence in order to examine the change in stem probability. For comparison, $p_{\text{stem}}(i)$ was calculated for a sequence with $N = 1,000$ by RNAplfold. Comparing this stem probability to that calculated by ParasoR for the same sequence revealed that they have little difference from each other (Figure S12). Then, we appended random flanking regions of N =100, 200, 1,000, 5,000, and 10,000 nt to both sides of the sequence, resulting in an increased disparity that eventually converged to a fixed difference of stem probability. This also suggests that flanking regions have enough influence to change structural preferences only around the sites in the range of ~ 1000 bases.

3.4 Varying the precisions of floating point numbers

We investigated how the numerical precision of floating point variables affects the ParasoR results. Figure S13 shows the differences in $p_{\text{stem}}(i)$ computed for 1,000-nt random sequences with increasing the length of flanking sequences using different floating point variables. While the differences in $p_{\text{stem}}(i)$ between 32-bit (`float`) and 128-bit (`long double`) variables increase rapidly, those between 64-bit (`double`) and 128-bit variables remain small, indicating using `double` or `long double` enables to safely analyze long RNAs without degradation of numerical precision.

CHAPTER 4

Consistency of thermodynamic prediction methods and experimental structure analyses

Recently, high-throughput structure analyses such as PARS have gained attention because of their wide application range and high productivity. However, there has been few comprehensive comparison between computational predictions and such analyses because of an inability to produce comparable amount of results via computational methods. In this section, we present a comparison of our prediction method and sliding-window methods with PARS data for human transcripts. Moreover, we performed a validation of ParasoR prediction for cis-regulatory elements structures listed in Rfam database.

4.1 PARS score manipulation

We used normalized PARS score datasets GSM1226157 and GSM1226158 obtained from renatured samples of GM128678 [6]. PARS scores were calculated for 74,211 genes from v_i and s_i data, which indicate read coverage normalized by sequence depth of samples after RNase V1 and S1 degradations, respectively; the maximum scores of v_i and s_i are $216,048$ and $252,848$ respectively, and the average scores are 1.21 for v_i and 1.20 for s_i. To obtain sequence information of transcripts for ParasoR prediction, we extracted 33,603 genes that are included in PARS dataset and mappable to our RefSeq gene database. Among them, we excluded genes for which there were no nucleotides with s_i or v_i higher than 0. Then, we calculated the PARS score for each position following the equation $\text{PARS} = \log_2(v_i + 5) - \log_2(s_i + 5)$, which is defined in [6]. Moreover, we used raw v_i and s_i scores for further filtering of obscure information. In particular, if a threshold is x, every position with $v_i + s_i$ less than x is excluded from the comparison.

4.2 Concordance between ParasoR predictions and PARS data

Next, we examined the concordance between PARS scores and two computational prediction measures; *stem probability* p_{stem} of each single nucleotide and *accessibility* of each 10-base unit. Both of these measures exhibited a significant correlation with the PARS score and in particular the stem probability exhibited a higher correlation coefficient than did accessibility. The Pearson's product-moment correlation coefficients were 0.212 ($p < 2.2e-16$) for stem probability and 0.00238 ($p < 0.019$) for accessibility. This seems to be because accessibility is a measure of being accessible for a series of nucleotides at a certain time, despite positional independency of stem probabilities and PARS scores. We also investigated the correlation of the average stem probability for each 32-mer $\bar{p}_{\text{stem}}$ with PARS score, and their correlation coefficient is 0.208 and still high ($p < 2.2e-16$). Accordingly, we used p_{stem} and $\bar{p}_{\text{stem}}$ as measures in the subsequent analyses.

We also tested a prediction regarding long maximal spans to determine the influence of a fixed W. Figure S14 shows the distribution of correlation coefficients calculated between PARS scores and $\bar{p}_{\text{stem}}$ for each 20-mer, which is a measure of consistency used in [6]. A distribution with $W = 200$ apparently contains much more regions of positive correlation between the PARS scores and $\bar{p}_{\text{stem}}$. When we increased W to $1,000$, there was little change to the distribution, though it also introduced a positive bias. According to this, differences between ParasoR prediction and PARS scores are expected to not be caused by ignoring distant base pairs but other factors instead, such as complicated secondary structures, higher dimensional structures, and experimental biases.

As previously described, the distribution of PARS scores is highly concentrated around 0 and has a long tail at both sides. Because sequence reads are likely to be assigned sparsely, almost uncovered and totally obscure positions might contributed to structural tendencies. Accordingly, we examined the influence of such obscure regions by filtering the sequence data with a variable threshold t for the minimum read coverage. Figure S15 shows scatter plots of PARS scores and p_{stem} when $t = 0$ and $t = 40$. Apparently, application of a strict t threshold removed data with PARS scores around 0 and elucidated a concentration of samples with positive PARS scores and ~ 0 p_{stem} or negative PARS scores and ~ 1 p_{stem}. Actually, the correlation coefficient between p_{stem} and PARS score at each position increases with threshold even though the two variables differ in sample size (Figure S16). Therefore, it is suggested that positions with rich information of sequence reads is likely to be consistent with ParasoR prediction.

Next, we drew receiver operating characteristic (ROC) curves of ParasoR, and two stochastic sliding-window methods, LocalFold and RNAplfold with PARS scores while varying thresholds to evaluate the accuracy of ParasoR in classifying regions as structured or accessible. To calculate p_{stem}, three energy models were applied to this analysis, Turner (2004), Andronescu (2007), and Andronescu (2010) energy model. Among them, Andronescu (2010) model was applied to ParasoR only because RNAplfold, and eventually LocalFold cannot read a parameter file of Andronescu (2010) model.

Firstly, we obtained thresholds of evenly spaced PARS scores between the maximum and minimum values to define true structured and accessible regions. Then for p_{stem}, we set thresholds from 0.0 to 1.0 to categorize each nucleotide as structured or accessible. The accuracy of the predictions was evaluated by setting true structured and accessible regions according to the PARS threshold with prediction of being structured or accessible according to the threshold of p_{stem}.

Firstly, we fixed a threshold for PARS score to a middle value (-0.39). Table 4.1 shows an area under the ROC curve (AUC) which was obtained by varying a threshold for classification of stem probabilities. AUC values of ParasoR are slightly less than those of LocalFold and RNAplfold and the result is consistent for three energy models. Since setting a maximal span W to 1,000 decreases AUC compared to AUC with $W = 200$, 200 is considered to be a proper maximal span for structure prediction of transcripts. For stem probability computation, Andronescu (2010) model, which was optimized using the Boltzmann likelihood algorithm, has a highest accuracy for prediction, followed by Andoronescu (2007), and Turner (2004) model.

Comparing an impact of energy model and software, selecting a different energy model produced a larger change of AUC than selecting a different method.

Next, we confirmed an accuracy of each tool with different conditions such as thresholds for PARS score and filtering by read depth. Figure S17 shows AUC values about classification of three tools for whole or filtered dataset, and for a different threshold for PARS score. As a result, both filtering of low-read depth region with a liberal threshold for PARS score and no filtering with strict thresholds increased AUC for prediction. Strict thresholds for PARS scores increased AUC of each tool, indicating high accuracy. Although a liberal threshold decreases the AUCs, filtering out rarely mapped regions increases the AUC as well as the accuracy of strict thresholds. Such tendency was common for Turner (2004) and Andronescu (2007) model.

We also made similar comparisons among 5′-UTR, CDS, and 3′-UTR analyses. Figure S18 shows the distribution of PARS scores for each annotation class. In both of distributions, with or without filtering, 5′-UTR is found to have more structured (> 0) and less accessible (< 0) regions compared to other groups. In [6], however, 5′-UTR was estimated to be more accessible using an average of PARS scores even though this measure is considered strongly influenced by large absolute values. In contrast, our prediction method evaluates each position equally within a range from 0 to 1. As an absolute read coverage at each position is also determined by the number of mapped reads,

Table 4.1: AUC values between PARS data and prediction tools for various conditions

Energy model	maximal span	ParasoR	LocalFold	RNAplfold
Turner (2004)	200	0.5977	0.6059	0.6067
Turner (2004)	1000	0.5969		
Andro (2007)	200	0.6101	0.6178	0.6185
Andro (2010)	200	0.6117		

it is supposedly an inappropriate measure to compare with p_{stem}. Accordingly, we calculated a median PARS score of each annotation class for comparison. In Figure 3(**C**)) in the main text, the median PARS scores for CDS, 5′-UTR, and 3′-UTR regions were 0.6041, 0.6280, and 0.5083, respectively. These scores agree with the order of average p_{stem} determined by ParasoR, which are 0.5956, 0.6248, and 0.58978, respectively, and furthermore, this order is robust to different filtering thresholds.

These analyses indicate that computational predictions and PARS analyses are highly congruent with each other in their ability to classify sequences as accessible or structured and in their measurement of regions based on functional annotation.

4.3 Concordance between ParasoR predictions and Rfam structures

To evaluate ParasoR prediction, we used CisReg dataset [17], which is compiled from the Rfam database and contains high-quality structures in the context of long sequences (detailed in Methods and Result section in the main text). For this dataset, Turner (2004) and Andronescu (2007) model were applied to ParasoR for computation of stem probability. In addition, we prepared dataset of genome and mRNA sequences whose length are longer than 3,000 nt (represented as genome_long and mRNA_long). Then, AUC was computed for the prediction of each tool, dataset, and energy model condition. As a result, ParasoR with Turner (2004) model showed the highest accuracy compared to other tools or ParasoR with Andronescu (2010) model regardless of the minimum length of sequences in dataset. It is an opposite result to that of PARS data.

We inferred it is due to the difference of stem probability distributions among different tools. According to the distribution of stem probability (Figure S20), ParasoR tends to predict a stem probability around 0 or 1 while LocalFold and RNAplfold produce gently sloping distributions. A bimodal distribution of ParasoR is similar to that of RNAfold (Figure S7(Center)) and appropriate to predict rigid structures such as those in the CisReg dataset. On the other hand, the distributions of LocalFold and RNAplfold are supposedly more suitable for PARS data, which has a bell-shaped smooth distribution. In fact, we can increase concordance with the PARS data by using the Andronescu model and averaging stem probabilities to reduce the bimodality of the distribution (see the right bar graph of Figure 3(A) in the main text). Figure S7(Center) shows that Andronescu (2010) model instead of Turner (2004) model decreases the bimodality of stem probability distribution. Figure S7(Left) shows that averaging of stem probability also makes a distribution close to unimodal. These indicate the lower accuracy of ParasoR for PARS data as compared to RNAplfold and LocalFold mainly results from the difference of their distributions.

CHAPTER 5

Genome-wide simulation by ParasoR

5.1 Linear regression of stem probability with sequence composition statistics

The stability of an RNA secondary structure is considerably affected by sequence composition because the secondary structure is comprised of base pairs that differ in strength depending on base type. However, sequence compositions are constrained by multiple biological functions such as coding for proteins, regulating gene expression, and so on. The determination of structural preferences requires a normalization of the influence of sequence composition. GC content is a feature that is commonly used for proper structure evaluation. Moreover, previous computational and experimental analyses have shown an apparent correlation between GC content and evaluation index [4]. Additionally, genome sequences are known to have even more complicated sequence biases, for example, AT/GC asymmetry or codon bias. As such, we designed a normalization method for genome-wide comparisons of stem probability using GC content and other, more complex features.

Using python 2.7 and the NumPy library, we implemented a linear regression using the average stem probability $\bar{p}_{stem}(i)$ with a ridge penalty. A simple implementation is available at our website (https://sites.google.com/site/cawatchm/). We examined the linear regression of stem probabilities with GC content as well as k-mer composition frequencies with $k = 3$ and 4. We used a least squares method for estimation of a parameter vector w and a regularization term λ, and its error function is formulated as below.

$$\frac{1}{2}\sum_{n=1}^{N}(y_n - w^T x_n)^2 + \frac{\lambda}{2}w^T w \tag{5.1}$$

In this formula, λ is a constant and w is a parameter vector in the same dimension as x. This equation is differentiable at w, and we obtain w to minimize this error function.

$$w = \left(\sum_n x_n^T x_n + \lambda I \right)^{-1} \left(\sum_n x_n y_n \right) \tag{5.2}$$

For example, when we selected a 32-mer window size and GC content as a regression feature, we calculate the GC content and average stem probability for each 32-mer fragment. Then, we set x_n and y_n as follows.

$$x_n = \left(\frac{1}{32}, \frac{\text{GC content}}{32} \right) \tag{5.3}$$

$$y_n = \text{average stem probability} \tag{5.4}$$

The first element of x_n shifts the mean stem probability to around 0.6. Similarly, x_n for the 3-mer composition regression is formulated as

$$x_n = \left(\frac{1}{32}, \frac{\#\text{AAA}}{32}, \frac{\#\text{AAC}}{32}, \cdots \frac{\#\text{UUU}}{32} \right) \tag{5.5}$$

Through this normalization process, 32-mer fragments with more than window size/4 ambiguous characters "N" were excluded.

For parameter optimization, we used stem probability data from both strands of human chromosomes and calculated the correlation coefficient between y_n and $w^T x_n$. In this analysis, 4-mer composition exhibited a higher correlation coefficient with stem probabilities than did GC content (Figure S21). Although other window sizes were tested for summarizing features, feature window sizes that were the same as those of average stem probabilities achieved the highest correlation coefficients. Based on this result, we used a 32-mer window size for feature calculation in the subsequent regressions of average stem probability $\bar{p}_{stem}(i)$. Figure S22(L) shows the result of λ estimation with GC content and 4-mer regression for human chromosome 1. An alteration of λ is observed to have almost no influence on the correlation between y_n and $w^T x_n$, likely because there is sufficient data to avoid overfitting. Hence, we set λ to 0 in subsequent analyses. Figure S22(R) shows the highest correlation coefficients of stem probability with GC content, 3-mer composition, and 4-mer composition. Figure S23 shows scatter plots of these GC content and 4-mer composition regressions with the actual stem probability for each 32-mer fragment $\bar{q}_{\text{stem}}$. Owing to its fewer parameters, GC content was outperformed by 4-mer composition in data normalization. Accordingly, we mainly discuss the result of stem probabilities normalized by 4-mer composition $\Delta\bar{p}_{\text{stem}}$ (computed by ($\bar{p}_{\text{stem}}$ − regression of $\bar{p}_{\text{stem}}$)). However, structure tendency determined by our analyses generally does not change between the GC and 4-mer normalization analyses. This normalization additionally reduces autocorrelation (Figure S24). According to this analysis, normalized stem probability indicates structure tendency within a narrow range compared to raw stem probability, which is highly influenced by regional heterogeneity in sequence composition.

5.2 Calculation time and memory

We applied ParasoR to human chromosomes and surveyed its resource and time efficiency by measuring the computational time and memory used by the HGC super computer.

5.2.1 Human chromosome

Three hundred computational nodes were used in parallel to analyze the human chromosomes. Figure S25 shows the time and memory usage averaged for the plus and minus strand of each chromosome. For this genome-wide calculation, ParasoR used 0.92 days at most for each chromosome, and the computational time is consistent with its linear dependency on sequence length N. Maximum memory usage was approximately 4 GB, which is typically available in personal computers.

We measured the computational time and memory usage for analyzing a concatenated human genome sequence ($\sim$ 3.1G) with an HGC super computer.

Table 5.1 shows the maximum computational time of each process in memory-saving mode for the human genome sequence. Using 3000 nodes, ParasoR requires at most 3.3 GB of memory for these processes. If we ideally run all processes in parallel, ParasoR can complete its probability calculation of the entire human genome in 18.2 hours.

5.2.2 Comparison of calculation time with other tools

For comprehensive mRNA analyses, we measured a running time of two of sliding-window methods, LocalFold and RNAplfold, for the calculation of stem probability or accessibility. Our dataset contains totally 140,031,550 nt of mRNA sequences and we distributed mRNA sequences into 300 multiple FASTA files to contain the same number of bases as much as possible. We computed elapsed times of RNAplfold and LocalFold for these 300 files and obtained an average calculation time (Table 5.2). For the sequence of 140M nt, A calculation time of ParasoR is estimated for Divide, Connect, and Probability calculation procedure by the slope and intercept value obtained from the running time of human chromosomes (Figure S25). This suggested that a running time of ParasoR is comparable to that of LocalFold. In addition, it is also possible to accelerate the structure calculation even more when we have already constructed database because we can skip the step of Divide and Connect procedure.

Table 5.1: Computational time for human genome sequence analysis

Procedure	Computational time (sec.)
1st Divide	22,084
1st Connect	403
2nd Divide	20,608
2nd Connect	5,932
Probability Calculation	16,508

5.2.3 Random sequences for the simulation of pre-mRNA and intron computation

While ParasoR is the only application which is available for human chromosomes and genome sequence, it is just a simulation and such long RNA is never transcribed in real. To clarify the utility of ParasoR parallelization for real transcripts, we applied ParasoR algorithm of single core and multiple core mode to the random sequences whose length is in the range of human pre-mRNA (from 1000 to 1,000,000 nt) and which have 50% GC content . Figure S26 shows an average elapsed time of ParasoR in the single core or multiple nodes of HGC super computer for 100 sequences. In ParasoR, the number of computer nodes is automatically reduced to an appropriate size depending on the balance of sequence length and maximal span. Thus, we showed the maximum number of available computing nodes in Figure S26. In addition, only a single sequence was applied to measure the running time of 100,000-nt and 1,000,000-nt sequence dataset due to the problem of long calculation time. We plotted a hundredfold running time for these conditions.

As a result, the running time proportionally increased according to the sequence length N. The result of ParasoR running time is less than tenth of that of Rfold. Although ParasoR with multiple nodes possesses an overhead cost for distribution and requires an additional process such as database construction, this parallelization achieved a substantial acceleration of the structure prediction for longer sequences as the number of computer nodes increases. In our human pre-mRNA dataset, the longest intron exceeds 1,000,000 (1,055,451) and ParasoR also showed its efficiency for the sequences of such length. Therefore, we concluded that ParasoR algorithm is still effective for pre-mRNA and full-length intron sequences.

5.3 Stem probability distribution for each genomic region

In order to understand the structural features of genome-sequences themselves, we simulated RNA secondary structures of chromosomes using ParasoR. Following the annotation rules outlined in Chapter 2, a distribution of both raw and normalized stem probabilities was generated for sequences across 10 types of annotation. In Figure S27(L), non-coding regions are observed to be more similar to introns than to CDS

Table 5.2: Computational time for human transcripts

Software	Computational time (sec.)
ParasoR	78839 (42906+22874+12958)
LocalFold	87547
RNAplfold	926

In parentheses, each time corresponds to Divide, Connect, and Probability calculation procedure.

regions. This comparison was repeated for log ratios of densities $\log(f_t(x)/f_{\mathrm{Intergenic}}(x))$ (t = genomic region); x normalized average stem probability $\Delta\bar{p}_{\mathrm{stem}}(i)$). Figure S27(L) shows that most of annotation categories in transcribed regions contain more structured fragments than do intergenic regions. Transcribed regions are thus more likely to be structured than are intergenic and repetitive regions. In addition, the distribution of these log ratios for CDS and repeat regions are notably different from those of the other annotation categories. In particular, repeat regions were unlikely to be sufficiently normalized. Thus, repetitive regions can be incorrectly estimated to be less structured. Figure S27(R) shows a similar result for the transcribed regions, though it includes repetitive fragments. Although the distribution of raw stem probabilities is less influenced by this inclusion, negative normalized stem probabilities increased the log ratio of each annotation category. To determine the structural tendency of such regions, it would be necessary to make comparisons among regions with the same repeat sequences in different positions or annotation categories. We also normalized stem probability by using GC content (Figure S28(L)), and the same tendency was obtained, though GC content did not normalize the unimodal distribution as well as 4-mer composition did.

When using stem probabilities of 1-mer-shuffled human chromosome 1 (detailed in Chapter 2), the random sequence was highly concentrated at 0.6 compared to intergenic regions (data not shown); on the other hand, a normalized stem probability distribution of shuffled sequence contains more structured regions (Figure S28(R)). This is likely because repetitive, GC-rich regions contribute to increases of structure across regions. This suggests that each genomic region is less structured than completely randomized sequence because of various biological constraints.

We confirmed these genome-wide analyses of structure preference using the mouse genome. The raw and normalized stem probabilities agree with those observed across the human genome (data not shown). As such, structural features may be shared between even distantly related species.

5.4 Genomic features in structured and accessible regions after normalization

In previous analyses, structural preferences were detected for each annotation category and compared with intergenic regions. Then, we investigated the correlation between genomic sequence features and structural preferences regardless of genomic annotation.

To determine the cause of these structure tendencies, accessible and structured regions were defined as having normalized stem probabilities of more than 0.3 and less than -0.3, respectively. Then, we computed the entropy of 2-mer and 3-mer frequencies in accessible and structured 32-mer regions as follows.

$$p(s) := \frac{\text{frequency of some k-mer } s}{\sum_{s'} \text{frequency of some k-mer } s'} \tag{5.6}$$

$$E_{\text{k-mer}} = \sum_{s} -p(s)\log_2 p(s) \tag{5.7}$$

For comparison, the entropy of chromosome 1 was also calculated. Figure S29 shows an example entropy distribution computed for 2-mer and 3-mer fragments on human chromosome 1. As expected, accessible and structured regions are biased toward a low entropy in comparison with all of chromosome 1. In particular, accessible regions are likely to have lower entropy than structured regions are.

The relationship between structure tendency and conservation was examined using PhastCons scores [5]. Figure S30 shows boxplots of normalized stem probability grouped by PhastCons score. Although there is a fluctuation of the median, no correlation was observed between conservation and structure tendency. In addition, the Pearson product-moment correlation coefficient was also not significant ($p > 0.05$).

CHAPTER 6

Structural preferences in mRNA and pre-mRNA

In the previous chapter, specific structural tendency was observed in transcribed regions of genome sequences. In this section, structural features of transcribed region were surveyed in the forms of mRNA and pre-mRNA.

6.1 Structural preferences of human and mouse transcripts

First, a distribution of average stem probabilities $\bar{p}_{\text{stem}}$ was calculated for mRNA and pre-mRNA in human and mouse transcriptomes (Figure S31). Analysis of these datasets yields similar distributions as those of full genome sequences, but repeat regions in the mouse genome produce a small peak among extremely unstructured regions, and this may be produced by distinctive repetitive elements in the mouse genome such as SINE B1 and B2 elements. We also calculated log ratios of densities $\log(f_t(x)/f_{\text{Intergenic}}(x))$ where $f_t(x)$ is the probability density of $\Delta\bar{p}_{\text{stem}}(i)$ at x for each annotation group t on mRNA and pre-mRNA sequences in the same way as in the genome analyses. Figure S31 shows that all groups, except for CDS, 5′-UTR, and repeat regions, are likely to be more structured than intergenic regions. While CDS and repeat regions are similar to those of entire genome sequences, 5′-UTRs are more accessible than their expected distribution based on sequence composition. This is likely explained by boundary effects such as the insertion of ambiguous characters or the phenomena in which the tips of sequences tend to be accessible (described in Chapter 3). This result is consistent across human and mouse transcriptomes.

6.2 Comparison of structural preferences with antisense and shuffled sequences

To evaluate structure tendency in transcribed regions, both antisense sequences and shuffled 3-mer sequences were used as a reference for comparison because they have the same GC content and sequence lengths. In the log ratio against intergenic regions, only antisense introns exhibit an opposite tendency compared with their sense strand counterparts (Figure S31(C)). This is likely because intronic sequences have an AT/CG asymmetry, which forces antisense regions to have a tendency against structure.

Next, we compared the distribution of sense sequences and these backgrounds using D statistics. To compare structural similarity, D statistics from the Kolmogorov-Smirnov test between sense strand and background distributions were evaluated using the ks2samp function in the SciPy library. Figure S32 shows D statistics of mRNA and pre-mRNA compared with the normalized stem probability distribution of antisense and shuffled 3-mer sequences. We used all of regions of shuffled sequences for comparison although repetitive regions and multi-annotated windows were excluded from sense and antisense sequences. Both intronic and exonic regions of transcripts significantly differed from their antisense (p-values: exon mRNA, 9.66e-20; exon, pre-mRNA $\sim$ 0.0; intron $\sim$ 0.0) and shuffled 3-mer sequences (p-value: mRNA, $\sim$ 0.0; pre-mRNA $\sim$ 0.0). All of D statistics are positive and this means that exonic region is unlikely to be structured compared to antisense and shuffled 3-mer sequences.

To determine the trend in the structural tendency of transcribed regions, we utilized signed Z-scores in a Wilcoxon's rank sum test using the distribution derived from the intergenic regions. Positive Z-score statistics indicate an inclination toward structured or less accessible bases compared to intergenic regions, while negative Z-score statistics indicate the opposite. Sense introns were likely to form structures while antisense introns exhibited an oppositely signed Z-score and thus tendency (Figures 6, S33). After splicing, sense strands of CDS regions exhibited a decrease of structure tendency after splicing, presumably owing to conformational alteration. Both 5′-UTRs and 3′-UTRs of the sense strand had a structural preference that was discordant with the antisense strand even though the distribution of 5′-UTR stem probabilities was uniformly affected by a boundary effect, as mentioned before.

6.3 Stem probability profile around start codons, termination codons, and splice sites

Around splice sites (SSs), specific secondary structures have been reported to have an influence on splicing efficiency. Hence, we examined structural tendencies with respect to location specificity including around the starts and ends of translation and SSs.

Around start and stop codons, our inferred profiles agree with experimental analyses and are highly influenced by GC content (Figures 4 and S35(L)). Our results also included 3-mer periodicity of structure and GC content, which produces the structural

periodicity [8].

We also investigated profiles around SSs using pre-mRNAs with at least three SSs. Figure S34 shows the average stem probability for each position within 40 nt around SSs grouped by the first, second, and third SSs from the front and back (where duplication was allowed). The first SSs clearly tend to be structured, while weaker disparities in stem probabilities are observed around the last SSs. This preference corresponds to high GC contents associated with CpG islands in or around promoter regions (Figure S35(C)). In the profile of average stem probability differences, however, there is little disparity among SSs (Figure S36).

Then, we computed the normalized average stem probability of 32-mer fragments $\Delta\bar{p}_{\mathrm{stem}}$ along the sense and antisense strands. After this, we computed Z-scores for the Wilcoxon's rank sum test of the distributions of each position surrounding these SSs with the intergenic distribution of such data. To calculate $\Delta\bar{p}_{\mathrm{stem}}$, each pre-mRNA sequence was fragmented into 32-mers from the start or end of each SS. Then, $\Delta\bar{p}_{\mathrm{stem}}$ was determined according to its distance from the nearest SS. Figure 7 in the main text shows that intronic regions in the sense strand tends to have base pairs. On the other hand, similar antisense strand regions have the opposite tendency (although this tendency is not statistically significant for every position after Bonferroni correction). Accordingly, introns have a tendency toward being structured even though it is not clear whether this preference comes from selective pressure on secondary structure or not. A peak positive Z-score is found in the polypyrimidine tract region of the sense strand, where RNA has been suggested to be single stranded to specifically prevent binding of U2AF65 [9]. Therefore, the polypyrymidine tract domains are suggested to be surrounded by the regions where stem structures are preferred for their sequence compositions.

CHAPTER 7

Conformational change after splicing event

In this chapter, we directly compare stem probabilities before and after splicing to ensure that normalization accurately accounts for the nature of various sequence biases. To directly compare mRNA and pre-mRNA, the difference in average stem probability for 32-mers was computed for the same exonic fragments of mRNA and pre-mRNA, which have the same base composition and thus are normalized via the k-mer regression in the same way

7.1 General tendency of conformational changes between mRNA and pre-mRNA

To understand the general tendency of alterations to the secondary structures, we computed $\Delta q_{\mathrm{stem}}(i) = \bar{p}_{\mathrm{stem,\ mRNA}}(i) - \bar{p}_{\mathrm{stem,\ pre\text{-}mRNA}}(i)$ for each 32-mer of the mRNA sequences. The distribution of $\Delta q_{\mathrm{stem}}(i)$ that is negatively biased, suggesting that secondary structures are more accessible in mRNA strands than pre-mRNA strands. In addition, $\Delta q_{\mathrm{stem}}(i)$ tends to be larger around SSs than those of distant positions because of the disappearance of splicing influences on secondary structures (Figure S37). By calculating a probability of crossing base pair around SSs, we showed that pre-mRNA tends to form base pairs crossing over both of SSs compared to mRNA (Figure S38). Thus, it is considered that intronic regions around SSs are likely to form base pairing with exonic regions against the exonic regions which are concatenated after splicing.

When we determined $\Delta q_{\mathrm{stem}}(i)$ according to annotation group, $\Delta q_{\mathrm{stem}}(i)$ was significantly more accessible for all annotation groups except 3′-UTRs. However, because the average distance from SSs differs for each annotation type, we filtered out almost unchanged samples with lower differences than a given threshold so that we could test structure differences only for substantially influenced regions. For each threshold, a number of post-accessible fragments exceeded the post-structural fragments in general

(Figure S39(L)). Wilcoxon's signed rank test indicated that CDS and non-coding exon were significantly accessible at every threshold (Table 7.2, 7.3, Figure S39(R)). In contrast, significant influences were not detected in other groups at other thresholds. Therefore, although other annotation groups have a tendency to become more accessible after splicing, their changes are supposed to be not significantly drastic.

7.2 Cause of drastic conformational changes

We investigated the factors that exacerbate structural changes by focusing on each SS. To discover the causes of drastic conformational changes during splicing, we tested the correlation between structure arrangement and four quantitative features: (1) gene expression, (2) GC content around mRNA SSs, (3) GC content around pre-mRNA SSs, and (4) intron length. The gene expression data consisted of CAGE data generated by FANTOM5 [10], and it was used to investigate the correlation of gene expression with structural influences of splicing. Each transcription start site expression count was averaged among different samples and removed as a tissue-specific gene if $\log_{10}(\text{medianexpression}) \leq 0.5$. Regarding other features, GC content around SSs was calculated for 200 nt windows at exon junctions as well as at 5′- and 3′- SSs.

As measures of conformational change, a median, maximum value, minimum value, and median of the absolute deviation were calculated for the distribution of the disparity in stem probability between mRNA and pre-mRNA $\Delta q_{\text{stem}}(i) = p_{\text{stem, mRNA}}(i) - p_{\text{stem, pre-mRNA}}(i)$ for 200 nt around SSs. To remove the duplicate influence of nearby SSs, each position was exclusively assigned to the nearest SS. SSs with fewer than 100 assigned bases were removed from subsequent analyses.

Median Δq_{stem} values were significantly correlated with both gene expression data and GC content of mRNA and pre-mRNA (Table 1 in the main text). A negative correlation between gene expression and median Δq_{stem} can be interpreted as a small number of regions becoming structured in highly expressed mRNAs. A median absolute deviation (MAD) of Δq_{stem} was obtained by $\text{median}(|\Delta q_{\text{stem}} - \text{median}(\Delta q_{\text{stem}})|)$ for each SS and used to indicate the magnitude of fluctuation. According to our analyses, the MAD of Δq_{stem} only significantly correlates with mRNA GC content (Table 1 in the main text). This suggests that the secondary structures of GC-rich mRNAs are likely to be less altered by the splicing process. In addition, the maximum and minimum Δq_{stem} values are shown in 7.1. The maximum Δq_{stem} is significantly correlated with all three of these features although the minimum Δq_{stem} is significantly correlated with mRNA GC content only. These results suggest that splicing specifically regulates structured RNA through its effects on translational efficiency.

7.3 Gene set enrichment analyses

To characterize the relationship between conformational arrangement and biological functions, gene set enrichment analyses were performed for genes that are conforma-

tionally changed between mRNA and pre-mRNA. We computed a median $\Delta q_{\text{stem}}(i)$ for each SS and defined a $\Delta \bar{q}_{\text{stem}}(u)$ for a given SS u. We produced subsets of post-accessible and post-structural regions by selecting the top 1% and 10% of SSs according to $\Delta \bar{q}_{\text{stem}}(u)$. The top 1% of post-accessible and post-structural SSs were mapped to 230 and 233 RefSeq genes, respectively, and those of top 10% were mapped to 2,087 and 2,195 RefSeq genes, respectively. Then we checked the distributions of the maximum intron length, average intron length, and number of introns for all and selected genes, respectively. The distributions of the top 1% and 10% of genes were significantly different from that of all the genes (Table 7.4), but the distribution of the top 1% of genes may have differed as a result of the small sample size.

Then, these genes were remapped to UniProt IDs (208 (post-accessible) and 204 (post-structural) IDs for top 1% genes, and 1,868 (post-accessible) and 2,000 (post-structural) IDs for the top 10%) for Gene Ontology (GO) enrichment analyses using Ontologizer [11]. Table 7.5 shows the GO terms that were detected as significantly enriched among influenced genes. There were no enriched GO terms among the top 1% of genes. However, 23 GO terms were enriched among the top 10% of post-accessible genes, though no GO terms were enriched among post-structural genes. For post-structural and post-accessible gene sets in the mouse transcriptome, the top 1% of post-accessible and post-structural genes exhibited 41 and 48 enriched GO terms, respectively. Among the top 10% of mouse genes, 6 and 10 GO terms were significantly enriched among the post-accessible and post-structural genes, respectively (Table 7.6). These GO terms overlapped with those identified in the human analyses-for example, organelle, catalytic activity, and phosphorus metabolic process. Among the top 1% of genes, however, there was no consensus between human and mouse genes in terms of GO term enrichment, and the distribution of intron features in human and mouse differed among all genes, as mentioned before. Hence, we applied a threshold of 10% for selecting post-accessible and post-structural genes.

Moreover, functional clustering was performed on the post-accessible gene set with DAVID [12], which eventually established 338 clusters based on this 10% threshold. The keywords of the clusters with the highest expression analysis systematic explorer (EASE) score were as follows: cytoskeleton (16.19), kinase (11.90), centrosome (9.18), actin-binding (7.12), and ubiquitin conjugate (6.42). Since the ubiquitin conjugate cluster

Table 7.1: Correlations between conformational changes and gene features

CC (p-value)	Maximum	Minimum
Expression	-0.018**	0.008
mRNA GC%	0.021**	-0.012**
Intron length	-0.006*	0.004

We tested statistical significance by using Pearson's correlation test. (*: $p < 0.05$, **: $p < 1e-3$ after Bonferroni multiple correction for 6 samples.)

includes genes related to muscle function such as Titin and to synaptic function such as Synaptoagmin-associated genes, this group seems to be involved in the construction and differentiation of muscle or metabolic pathways. We also applied DAVID to post-accessible and post-structural mouse genes. For post-accessible genes, the extracted functional clusters with the highest EASE score were as follows: cytoskeleton (14.60), ATP-binding (13.11), centrosome (7.33), cell division (5.72), and actin-binding (5.42). For post-structural genes, the extracted functional clusters with the highest EASE scores were as follows: ATP-binding (16.57), kinase (6.19), C2 domain (4.23), and cytoskeleton (4.17).

7.4 Difference of structure propensity between sense and antisense sequence

We suggested the structure propensity of intronic regions differ from each other in sense and antisense strand in the main text. That tendency was shown only after the normalization by the k-mer composition. In this section, we thus examined the difference of structure propensity between sense and antisense sequence about intact stem probability, partition function, and γ-centroid structure computed by energy models.

To extract the structure propensity bias of the energy model, we produced random sequences of 200-nt long with the specific GC or GU content, and then computed the partition function of them and their complementary sequences. As a result, the partition function of random sequences showed few deviation between sense and its antisense sequence, and the relationships of the partition function with varying GC content is almost linear (Figure S40(L)). On the other hand, the biased GU content produced the non-linear relationships between the partition function of sense and antisense sequences (Figure S40(C)). This is supposed to be mainly caused by the disappearance of G-U base pairs in the complementary sequence. In addition, random sequences having the same GU content still showed the deviation of the partition function, indicating the existence of more complex biases in both of Turner and Andronescu energy model.

Next, the comparison of stem probability was applied to *NEXN* gene (NM_144573) (Figure 9 in the main text). To examine the relationship of stem probability between sense and antisense strand, we calculated the difference of stem probability (mRNA p_{stem} - pre-mRNA p_{stem}) for each position of sense and reversed antisense sequences. Figure S41 shows the average difference of stem probability and GU content for each 32-nt window on the mRNA and pre-mRNA sequence. The average difference of stem probability is clearly proportional to the GU content of the window in the sense strand. Also, pre-mRNA is longer than mRNA so that it contains more biased positions in terms of the difference of stem probability as well as GU content.

We also used *NEXN* gene to investigate the difference of γ-centroid structures in sense and antisense strand. Splicing out of the 3rd intron sequence was observed to produce the largest difference of stem probabilities on the peripheral exonic region among all of 12 introns in *NEXN* gene (Figure S9). In Figure S42, the partial γ-centroid

structures were extracted around the 3rd intron region of *NEXN* gene in sense and antisense strand (visualized using http://biojs.io/d/drawrnajs). γ-centroid structures were originally predicted for the whole sequences, but only the partial structures around the 3rd intron were extracted for visualization not to have any base pair which has a probability higher than 0.5 with the exterior sequences. Although the longest stem loop is roughly conserved, 2 of 3 stem loops in the right side of the sense structure disappeared from the multi-loop in the antisense structure. The length of stem loop in the left side also became shorter in the antisense structure, resulting the long multi-loop was newly formed in it.

Furthermore, the consensus sequence of human Alu repeat, shown as below, was extracted from RepBase [13] for γ-centroid structure prediction.

- Alu-Jo subfamily
  GGCCGGGCGCGGUGGCUCACGCCUGUAAUCCCAGCACUUU
  GGGAGGCCGAGGCGGGAGGAUUGCUUGAGCCCAGGAGUUC
  GAGACCAGCCUGGGCAACAUAGCGAGACCCCGUCUCUACA
  AAAAAUACAAAAAUUAGCCGGGCGUGGUGGCGCGCGCCUG
  UAGUCCCAGCUACUCGGGAGGCUGAGGCAGGAGGAUCGCU
  UGAGCCCAGGAGUUCGAGGCUGCAGUGAGCUAUGAUCGCG
  CCACUGCACUCCAGCCUGGGCGACAGAGCGAGACCCUGUC
  UCAAAAAAAAAAAAAAAAAAAAAAAAAAAAAA

Figure S43 shows the γ-centroid structure ($\gamma = 1$) of the Alu-Jo subfamily sequence in sense and antisense strand. Although the structure of the sense sequence shows two arms of stem loop, antisense one possesses long multi loop as well.

Therefore, it is concluded that the predicted structure and structure propensity of sense and antisense sequences possibly show the critical difference depending on their sequence composition.

Table 7.2: Statistical test of substantial conformational changes for each annotation category in the human genome

threshold	p-value of CDS	non-coding exon	5′-UTR	3′-UTR
0.00	0.0	2.53e-244	3.53e-147	0.42
0.05	0.0	0.0	3.87e-209	0.00032
0.10	0.0	0.0	2.98e-189	1.37e-07
0.15	0.0	2.80e-259	1.50e-130	1.68e-08
0.20	0.0	7.45e-173	1.48e-79	0.0017
0.25	0.0	3.55e-105	1.22e-20	0.019
0.30	0.0	1.16e-61	8.50e-10	0.054
0.35	0.0	3.67e-26	0.0061	0.093
0.40	4.46e-279	4.00e-15	0.51	040
0.45	7.26e-164	4.08e-07	0.69	0.35
0.50	2.60e-87	7.29e-05	0.27	0.11

Table 7.3: Statistical test of substantial conformational changes for each annotation category in the mouse genome

threshold	p-value of CDS	non-coding exon	5′-UTR	3′-UTR
0.00	0.0	2.37e-104	1.66e-108	1.08e-08
0.05	0.0	3.92e-55	3.84e-140	7.60e-07
0.10	0.0	1.96e-62	1.01e-114	0.00121
0.15	0.0	1.71e-55	4.61e-84	0.00876
0.20	0.0	8.60e-42	1.66e-50	0.0149
0.25	0.0	1.26e-26	1.05e-27	0.000460
0.30	0.0	2.14e-13	3.30e-07	0.000117
0.35	1.90e-279	1.78e-07	4.59e-05	0.00642
0.40	5.09e-155	6.16e-05	0.00873	0.123
0.45	7.03e-90	0.0107	0.0918	0.109
0.50	1.25e-54	0.228	0.217	1.0

Table 7.4: Statistical tests on the distribution of intron features of the top 1% and 10% of genes

feature	1% post-acc	1% post-str	10% post-acc	10% post-str
maximum intron length	6.26e-15	2.46e-08	2.2-e16	2.2e-16
average intron length	5.80e-05	0.042	2.2e-16	0.0026
# of intron	2.2e-16	2.2e-16	2.2e-16	2.2e-16

We computed Bonferroni-corrected p-values by using Wilcoxon's rank sum test.

Table 7.5: Enriched GO terms among post-accessible genes in human

GO	term	p-value
GO:0044422 (C)	organelle part	0.0011
GO:0044428 (C)	nuclear part	0.0025
GO:0006996 (P)	organelle organization	0.0104
GO:0007059 (P)	chromosome segregation	0.0104
GO:0007049 (P)	cell cycle	0.0104
GO:0003723 (F)	RNA binding	0.0104
GO:0071840 (P)	cellular component organization or biogenesis	0.0104
GO:0032991 (C)	macromolecular complex	0.0104
GO:0051246 (P)	regulation of protein metabolic process	0.0104
GO:0000723 (P)	telomere maintenance	0.0104
GO:1902589 (P)	single-organism organelle organization	0.0113
GO:0031974 (C)	membrane-enclosed lumen	0.0120
GO:0051301 (P)	cell division	0.0160
GO:0036094 (F)	small molecule binding	0.0175
GO:0032268 (P)	regulation of cellular protein metabolic process	0.0216
GO:0033044 (P)	regulation of chromosome organization	0.0216
GO:0005515 (F)	protein binding	0.0301
GO:0032878 (P)	regulation of establishment or maintenance of cell polarity	0.0318
GO:0045595 (P)	regulation of cell differentiation	0.0382
GO:0033043 (P)	regulation of organelle organization	0.0403
GO:0043226 (C)	organelle	0.0403
GO:0043228 (C)	non-membrane-bounded organelle	0.0404
GO:0044427 (C)	chromosomal part	0.0439

Table 7.6: Enriched GO terms among post-accessible and post-structural genes in mouse

GO	term	p-value
GO:0005488 (F)	binding	2.14e-5
GO:0006996 (P)	organelle organization	0.024
GO:0044422 (C)	organelle part	0.024
GO:0071840 (P)	cellular component organization or biogenesis	0.034
GO:0043226 (C)	organelle	0.034
GO:0016817 (F)	hydrolase activity, acting on acid anhydrides	0.034
GO	term	p-value
GO:0003824 (F)	catalytic activity	0.016
GO:0005524 (F)	ATP binding	0.016
GO:0006793 (P)	phosphorus metabolic process	0.016
GO:0043412 (P)	macromolecule modification	0.016
GO:0032559 (F)	adenyl ribonucleotide binding	0.016
GO:0036094 (F)	small molecule binding	0.016
GO:0030554 (F)	adenyl nucleotide binding	0.016
GO:0019992 (F)	diacylglycerol binding	0.020
GO:0097367 (F)	carbohydrate derivative binding	0.027
GO:0034660 (P)	ncRNA metabolic process	0.028

CHAPTER 8

Procedures to compute p-values for some nonparametric tests

Our experiments to detect structural preferences require computing p-values for very large datasets that cannot be handled by popular statistical analysis tools such as the "R" programming environment [14]. Here, we describe the procedures to compute p-values for the analyses in which R could not be used.

8.1 Wilcoxon's rank sum test

We used Wilcoxon's rank sum test to estimate the structural preferences of each transcribed region relative to intergenic regions (Figures 6 and 7 in the main text). Since the datasets were very large ($\sim 10^8$), we kept only the histograms of stem probabilities, which necessitates the handling of tied values. Since it is impossible to compute the exact null distribution for large data sets with ties, we used the normal approximation, in which p-values are computed from the asymptotic normal distribution for a Z-score statistic of the test. Let $X = \{X_i | i = 1, \ldots, n_X\}$ and $Y = \{Y_j | j = 1, \ldots, n_Y\}$ be the two datasets to be compared, then the Z-score of the Wilcoxon's rank sum test is given

by

$$
\begin{aligned}
Z &= \frac{W - \mu - c(W - \mu)}{\sigma} \\
W &= \sum_{i=1}^{n_X} \mathcal{R}_n(X_i) \\
\mu &= \frac{n_X(n+1)}{2} \\
\sigma^2 &= \frac{n_X n_Y}{12} \left\{ (n+1) - \frac{1}{n(n-1)} \sum_{g=1}^{G} \left(m_g^3 - m_g \right) \right\} \\
c(u) &= 0.5 \ \mathrm{sign}(u),
\end{aligned}
$$

where $n = n_X + n_Y$ and $\mathcal{R}_n$ is the rank function applied to the merged dataset $X \cup Y$, which returns a rank $\in [1, n]$ for each value based on the increasing order of data values. If there are tied values, their average rank is assigned to every element within the tie group. G represents the number of tie groups and m_g represents the number of elements in tie group g.

In the above formula, $\mathrm{sign}(u)$ represents the sign function, which is defined by

$$
\mathrm{sign}(u) = \begin{cases} 1 & \text{if } u > 0 \\ 0 & \text{if } u = 0 \\ -1 & \text{if } u < 0 \end{cases}
$$

and $c(u)$ is the continuity correction term. Under the null hypothesis, the Z-score asymptotically follows the standard normal distribution.

8.2 Wilcoxon's signed rank test

Wilcoxon's signed rank test compares two datasets X and Y of paired observations $\{(X_i, Y_i) | i = 1, \ldots, n\}$. It was used to detect systematic changes of structural strengths due to splicing (Figure 8 in the main text). There are some ambiguities for the definition of the Z-score in the cases in which there are observations (X_i, Y_i) such that $X_i = Y_i$. In the wilcox.test() function of R, such observations are simply ignored, and the Z-score is

defined by

$$
\begin{aligned}
Z &= \frac{S - \mu - c(S - \mu)}{\sigma} \\
S &= \sum_{i=1}^{n'} \mathrm{sign}(X'_i - Y'_i)\mathcal{R}_{n'}(|X'_i - Y'_i|) \\
\mu &= \frac{n'(n'+1)}{4} \\
\sigma^2 &= \frac{n'(n'+1)(2n'+1)}{24} - \frac{1}{48}\sum_{g=1}^{G}\left(m_g^3 - m_g\right)
\end{aligned}
$$

where dataset $\{(X'_i, Y'_i)|i = 1, \dots, n'\}$ includes only elements with $X'_i \neq Y'_i$. $\mathcal{R}_{n'}(|X'_i - Y'_i|)$ returns a rank $\in [1, n]$ for each i based on the numerical order of $|X'_i - Y'_i|$. If there are tied values for $|X'_i - Y'_i|$, then the average rank is assigned as in Wilcoxon's rank sum test. The size m_g of tie group g is defined for the data $\{|X'_i - Y'_i||i = 1, \dots, n'\}$. Under the null hypothesis, this Z-score approximately follows the standard normal distribution.

8.3 Conover's squared ranks test for testing equal variances between samples

Conover's squared ranks test is a nonparametric test for detecting differences of variance among multiple samples [15]. We used the test to show that the distribution of normalized average stem probabilities $\Delta\bar{p}_{\mathrm{stem}}$ in the coding regions has significantly smaller variance than that of intergenic regions (Figure 5 in the main text). Let $X = \{X_i|i = 1, \dots, n_X\}$ and $Y = \{Y_j|j = 1, \dots, n_Y\}$ be the two datasets compared and $D_{Xi} = |X_i - \bar{X}|$ and $D_{Yj} = |Y_j - \bar{Y}|$ be the absolute deviations from their sample means. Then, in the normal approximation, the following Z-score approximately follows the normal distribution ([15]):

$$
\begin{aligned}
Z &= \frac{T - \mu}{\sigma}, \\
T &= \sum_{i=1}^{n_X} \left[\mathcal{R}_n(D_{Xi})\right]^2 \\
\mu &= n_X \bar{\mathcal{R}_n^2} \\
\sigma^2 &= \frac{n_X n_Y}{n-1}\left\{\bar{\mathcal{R}_n^4} - \left(\bar{\mathcal{R}_n^2}\right)^2\right\} \\
\bar{\mathcal{R}_n^2} &= \frac{1}{n}\left\{\sum_{i=1}^{n_X}\left[\mathcal{R}_n(D_{Xi})\right]^2 + \sum_{j=1}^{n_Y}\left[\mathcal{R}_n(D_{Yj})\right]^2\right\} \\
\bar{\mathcal{R}_n^4} &= \frac{1}{n}\left\{\sum_{i=1}^{n_X}\left[\mathcal{R}_n(D_{Xi})\right]^4 + \sum_{j=1}^{n_Y}\left[\mathcal{R}_n(D_{Yj})\right]^4\right\}
\end{aligned}
$$

where $n = n_X + n_Y$ and $\mathcal{R}_n$ is the rank function applied to the merged dataset $\{D_{Xi}\} \cup \{D_{Yj}\}$. As in the previously described procedure, the average rank is assigned for tied values.

8.4 Computing p-values from Z-scores

For a given Z-score z, the p-value $p(z)$ for the hypothesis test on the *more significant* side is computed by:

$$p(z) = \int_{-\infty}^{-|z|} \frac{1}{\sqrt{2\pi}} \exp\left(-\frac{x^2}{2}\right) dx = \frac{1}{2}\text{erfc}\left(\frac{|z|}{\sqrt{2}}\right)$$

where $\text{erfc}(x)$ is the complementary error function. When the computation of $p(z)$ based on this formula returned zero for large $|z|$, we used the exact upper bound of $\log_{10}(p(z))$ [16]

$$\log_{10}(p(z)) < -\left(\frac{z^2 + \ln(2\pi z^2)}{2\ln(10)}\right)$$

which can be derived from the continued fraction representation of $\text{erfc}(x)$

$$\text{erfc}(x) = \frac{e^{-x^2}}{\sqrt{\pi}}\left(\frac{1}{x+}\frac{1/2}{x+}\frac{1}{x+}\frac{3/2}{x+}\frac{2}{x+}\cdots\right) < \frac{e^{-x^2}}{x\sqrt{\pi}}$$

where only the first term in the continued fraction is taken into account on the right hand side.

Bibliography

[1] Kiryu H. *et al.* (2008) Rfold: an exact algorithm for computing local base pairing probabilities. *Bioinformatics.*, **24** (3), 367–373.

[2] Kiryu H. *et al.* (2011) A detailed investigation of accessibilities around target sites of siRNAs and miRNAs. *Bioinformatics.*, **27** (13), 1788–1797.

[3] Fukunaga T. *et al.* (2014) CapR: revealing structural specificities of RNA-binding protein target recognition using CLIP-seq data. *Genome Biol.*, **15** (1), R16.

[4] Trotta E. (2014) On the normalization of the minimum free energy of RNAs by sequence length. *PLoS One.*, **9**(11), e113380.

[5] Siepel, Adam, and David Haussler. (2005) Phylogenetic hidden Markov models. *Statistical methods in molecular evolution*, Springer New York.

[6] Wan Y. *et al.* (2014) Landscape and variation of RNA secondary structure across the human transcriptome. *Nature.*,

[7] Mori R. *et al.* (2014) Efficient calculation of exact probability distributions of integer features on RNA secondary structures. *BMC Genomics.*, **15 Suppl 10**, S6.

[8] Shabalina SA. *et al.* (2006) A periodic pattern of mRNA secondary structure created by the genetic code. *Nucleic Acids Res.*, **34**(Suppl. 8), 2428–2437.

[9] Warf MB. *et al.* (2010) Role of RNA structure in regulating pre-mRNA splicing. *Trends Biochem Sci.*, **35**(3), 169–178.

[10] FANTOM Consortium and the RIKEN PMI and CLST (DGT). *et al.* (2014) A promoter-level mammalian expression atlas. *Nature.*, **507**(7493), 462–470.

[11] Bauer S. *et al.* (2008) Ontologizer 2.0–a multifunctional tool for GO term enrichment analysis and data exploration. *Bioinformatics.*, **24**(14), 1650–1651.

[12] Huang da W. *et al.* (2009) Bioinformatics enrichment tools: paths toward the comprehensive functional analysis of large gene lists. *Nucleic Acids Res.*, **37**(1), 1–13.

[13] Jurka, J., Kapitonov, V.V., Pavlicek, A., Klonowski, P., Kohany, O., Walichiewicz, J. (2005) Repbase Update, a database of eukaryotic repetitive elements. *Cytogentic and Genome Research*, **110**, 462–467.

[14] R Core Team. (2013) R: A Language and Environment for Statistical Computing. *http://www.R-project.org/*

[15] Conover,W.J. (1999) *Practical Nonparametric Statistics, 3rd ed.*. John Wiley & Sons, New York, USA.

[16] Karagiannidis G.K. *et al.* (2007) An Improved Approximation for the Gaussian Q-Function. *IEEE Communication Letters*, **11** (8), 644–646.

[17] Lange SJ., Maticzka D., Möhl M., Gagnon JN., Brown CM., and Backofen R. (2012) Global or local? Predicting secondary structure and accessibility in mRNAs. *Nucleic Acids Res.*, **40**(12), 5215-5226.

Supplementary Figures

Parallel computation of genome-scale RNA secondary structure to detect structural constraints on human genome

Risa Kawaguchi and Hisanori Kiryu April 25, 2016

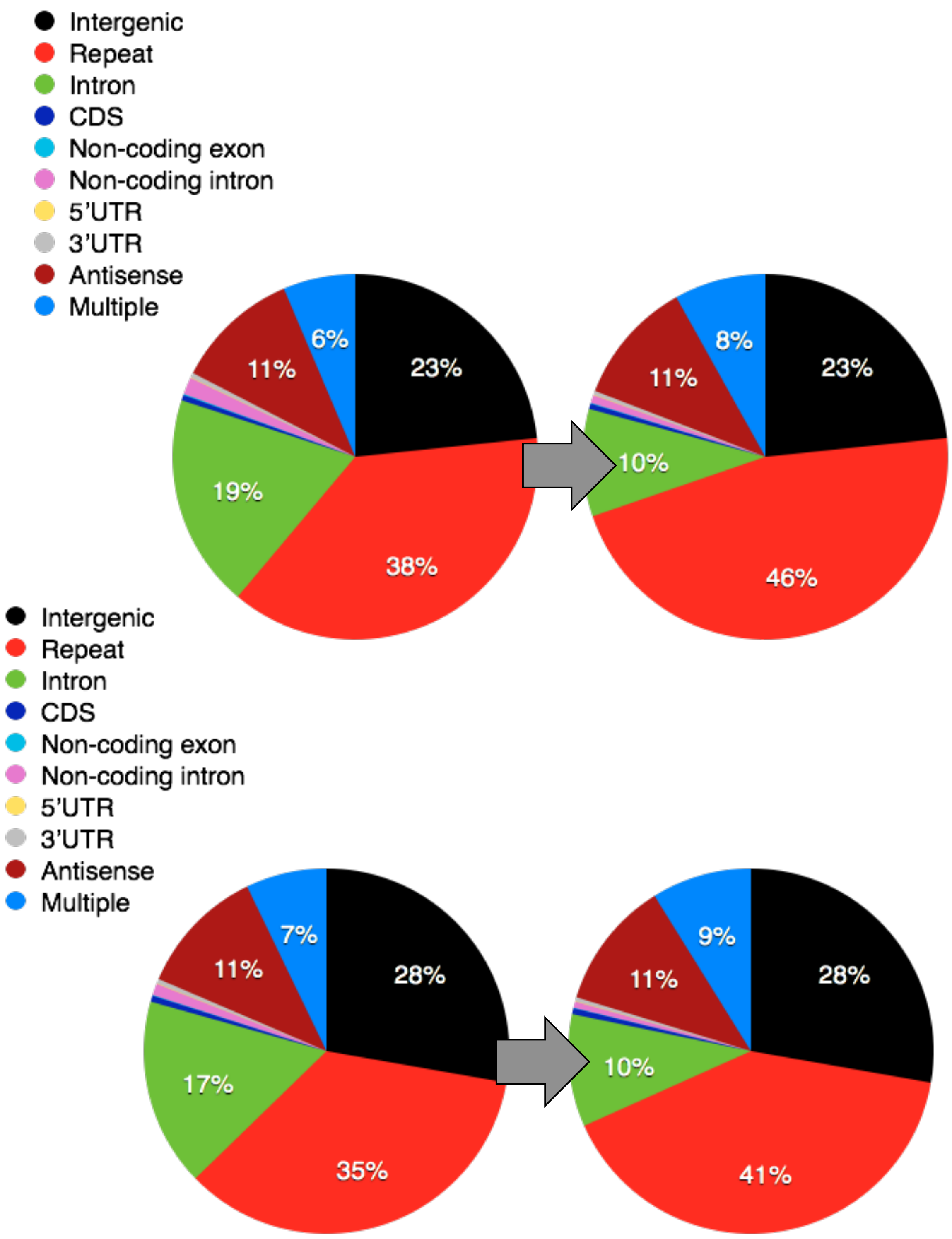

Figure S1: Ratio of annotation groups on genome. Percentage of 10 annotation groups on human (Top) and mouse (Bottom) genome based on 32-mer fragmentations **before** (Left) and **after removing repetitive elements** (Right) from the groups of transcribed regions and Antisense.

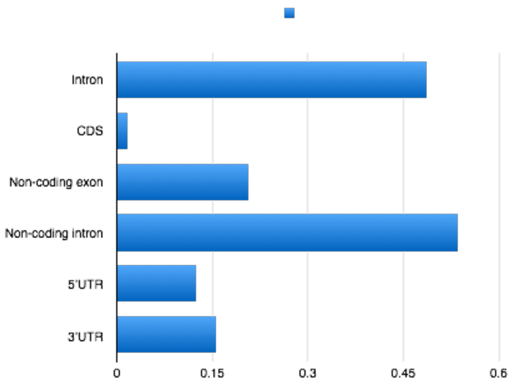

Figure S2: Ratio of repeat regions included in each annotation group of transcribed regions. Almost half the number of Intronic regions of coding and non-coding RNA is classified into the repetitive elements.

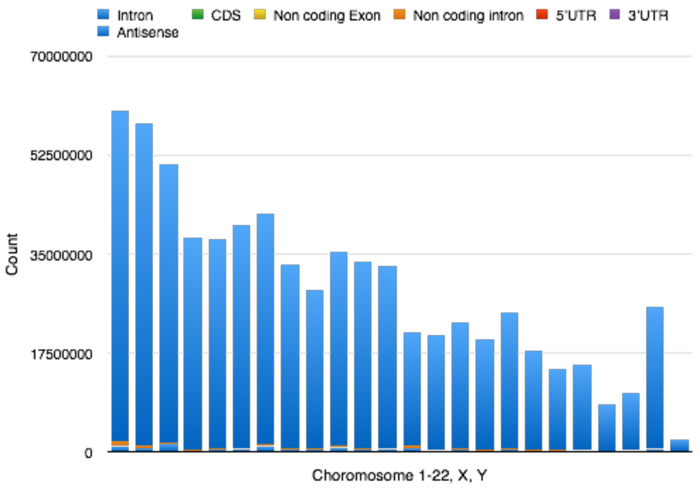

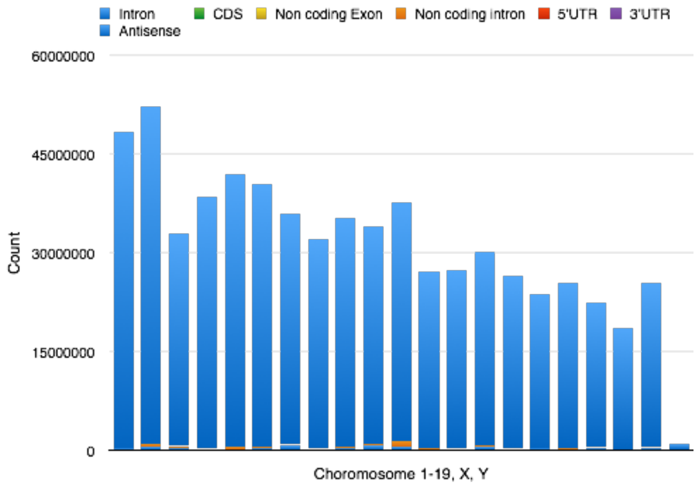

Figure S3: A property of regions classified into Antisense regions. Bar graphs show nucleotide counts located in antisense strand of the transcribed region for each chromosome of **human** (Left) and **mouse** (Right). Most of antisense regions belong to Antisense group while a small number of nucleotides are annotated as the other annotation groups.

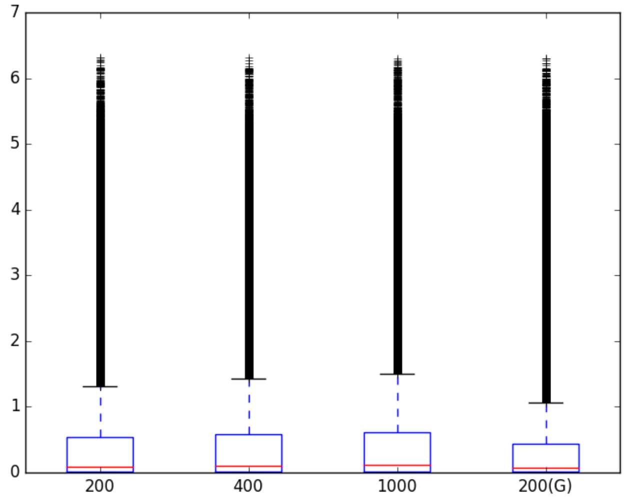

Figure S4: Distribution of the ratio of DP variables. Boxplots show $\Delta\alpha$ calculated for human mRNA sequences with different maximal spans (200, 400, and 1000 as shown in X-axis) and human chromosome 1 with W=200 (the rightmost boxplot). $\Delta\alpha$ was randomly sampled once per 1,000 nt.

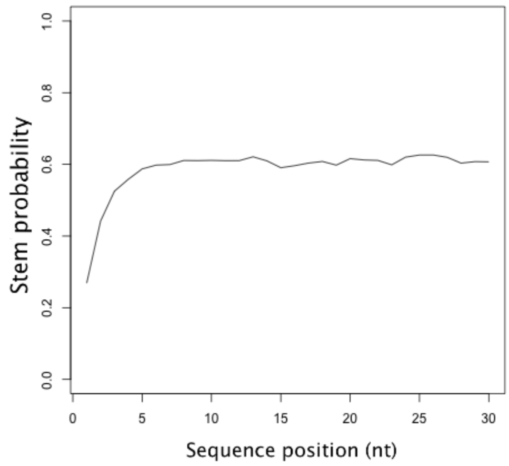

Figure S5: Regional property of average stem probability. Averaged p_{stem} was computed based on Turner energy model (1999) with W=1000 (which is equal to the sequence length). Several bases at the end of sequence show substantial declines of p_{stem}.

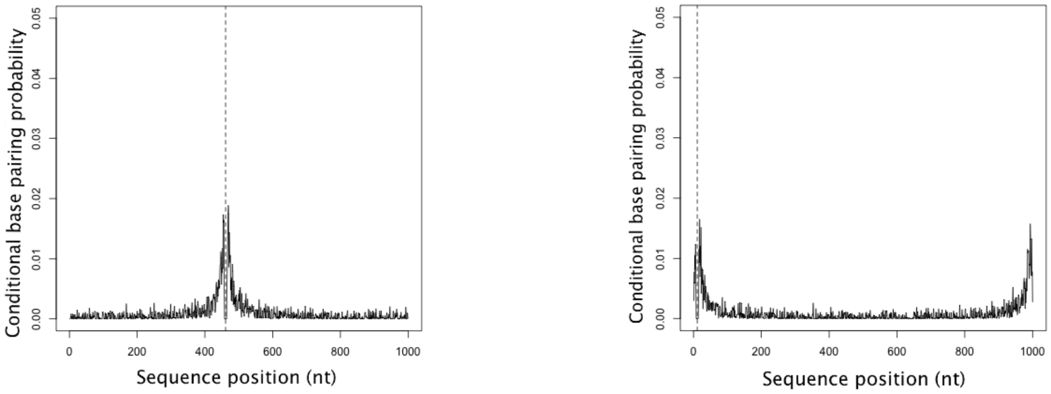

Figure S6: Regional property of Conditional base pairing probability (Cbpp). Cbpp was calculated by ParasoR using Turner energy model (1999) with W=1000 (which is equal to the sequence length). Y-axes show Cbpp between two positions; one is indicated by a dotted line, and another is shown in each x-coordinate.

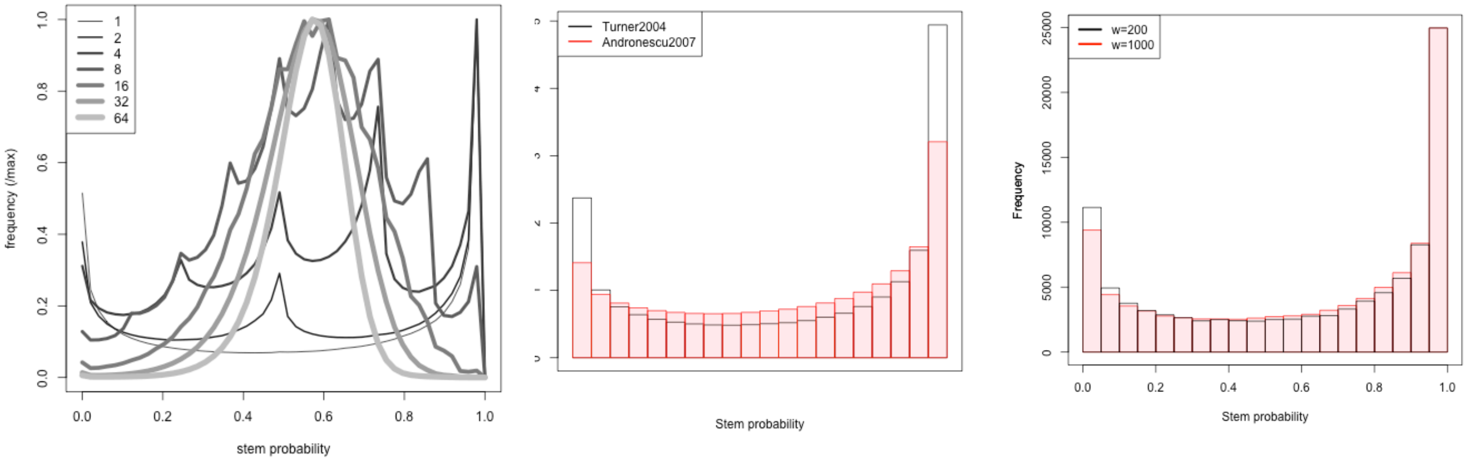

Figure S7: Property of stem probability. (Left) Average stem probability of each window size (1-64 nt) for human genome data. The distribution of average stem probabilities is bimodal for small window sizes, and becomes close to unimodal as the window size increases. (Center) Distribution of stem probability computed by Turner (2004) and Andronescu (2007) energy model using our transcript data. (Right) Distribution of stem probability for a random sequence of 100,000-nt length which has 50% GC content, with the condition of W=200 and 1000.

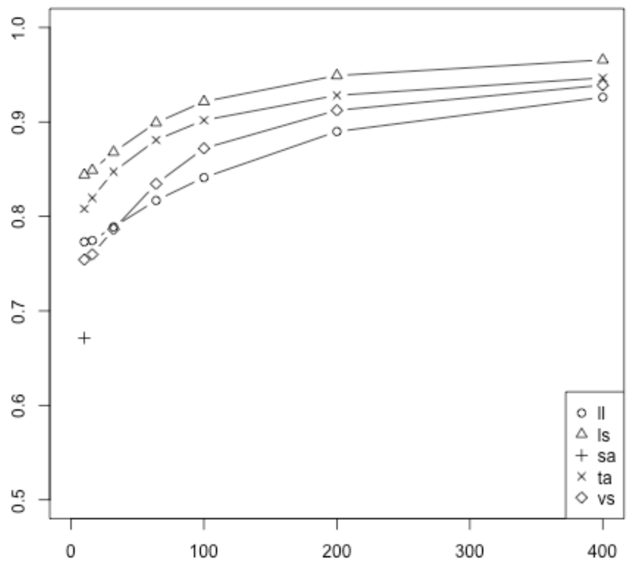

Figure S8: Correlation with sliding window with varying the parameter for structure prediction. Correlation coefficient of averaged p_{stem} (W=200) was computed with accessibility (sa+), averaged p_{stem} by the different energy model (ta×: Turner (2004) versus Andronescu (2007) energy model), or averaged p_{stem} of the different length of maximal span (vs◇: W=100, ls△: W=150, and ll○: W=400) computed by Turner energy model (2004). X-axes correspond to the window size for averaging of p_{stem}. Although a longer window size increases a value of correlation coefficient, it may reduce a characteristic for each region.

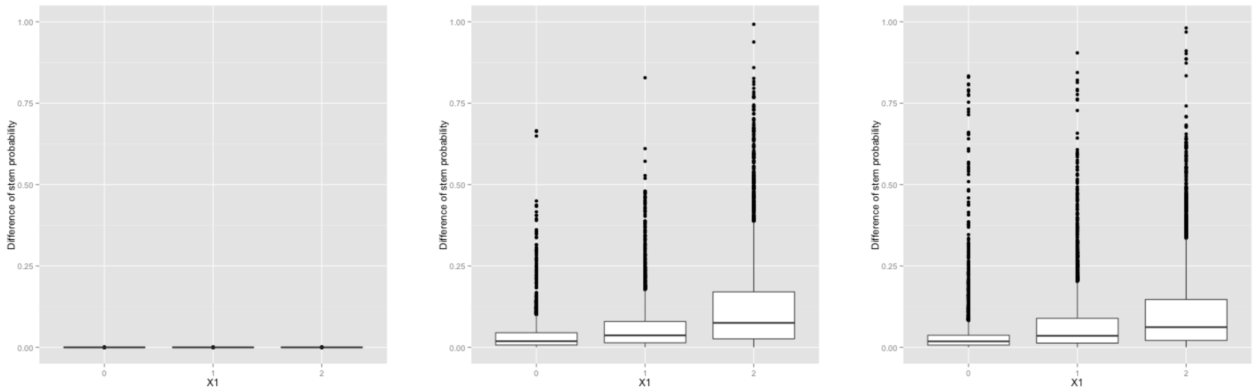

Figure S9: Boxplots for the differences of stem probability between RNAplfold and ParasoR. We used the random sequence of different length ((Left) N=1,000, (Center) 3,000, and (Right) 5,000). Each boxplot corresponds to the result from the different maximal span of base pairings (W=100, 200, and 400) in ParasoR and RNAplfold (the window size is set to the sequence length).

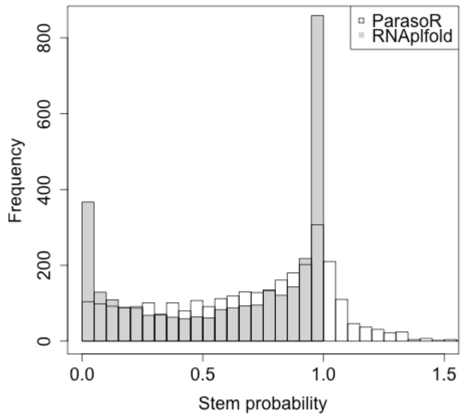

Figure S10: Overflow of stem probability. Histogram of stem probabilities of ParasoR and RNAplfold for a sequence of 3,000-nt length with W=200 (and L=3,000).

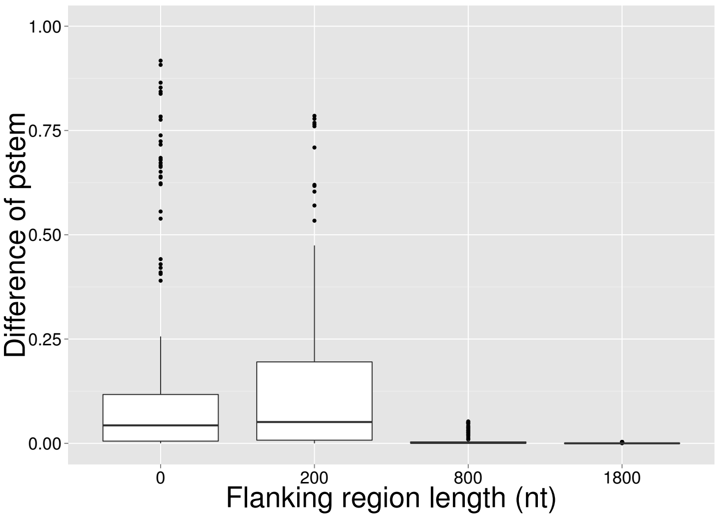

Figure S11: Difference of stem probability between ParasoR and RNAplfold. Boxplots of the differences of p_{stem} between ParasoR and RNAplfold for the specific region of 200-nt length on human chromosome 1 with a different length of flanking region.

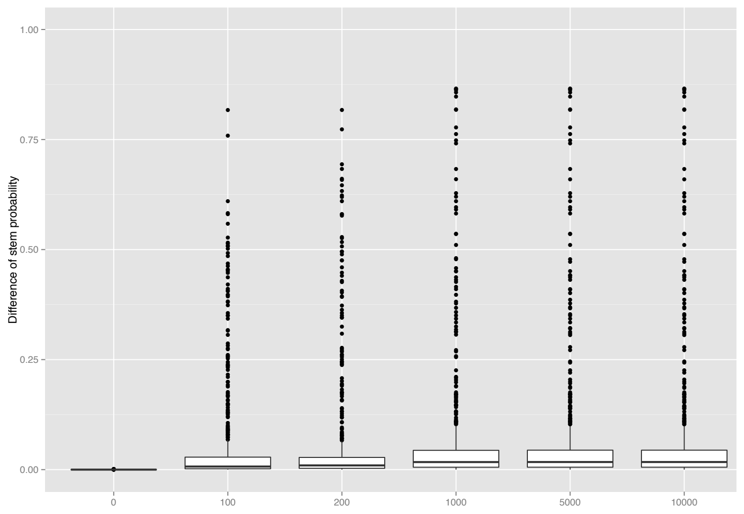

Figure S12: Difference of stem probability with flanking regions. Boxplots of the differences of p_{stem} between RNAplfold and ParasoR with the flanking region of variable length at both sides. P_{stem} was computed for the sequence (N=1000) by RNAplfold with L=200 and W=1000. We then computed stem probability of the same sequence by ParasoR with the flanking region of 0, 100, 200, 1,000, 5,000, and 10,000-nt length (represented in x-axis).

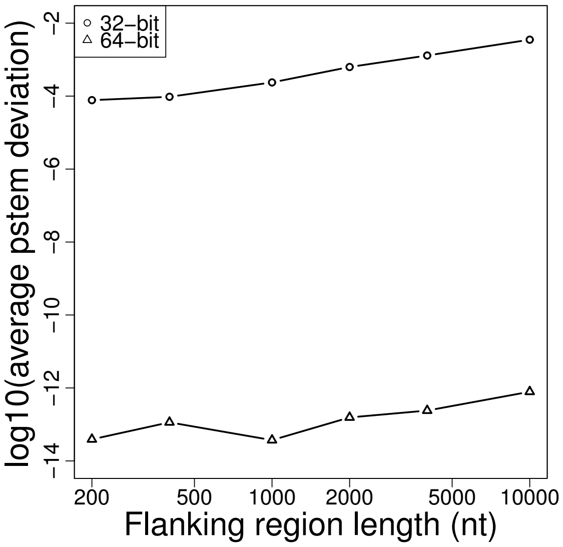

Figure S13: Influence of the different numerical precision. Average absolute deviation of p_{stem} on the **different conditions of the numerical precision of floating point variables**. p_{stem} was computed for the 1,000 bases located at the center of random sequences with the flanking region of the dfifferent length (shown in x-axis) using 32-, 64-, and 128-bit floating variables. The y-axis represents the deviation of stem probabilities that were computed using 32-bit or 64-bit floating point variables from those with 128-bit variables.

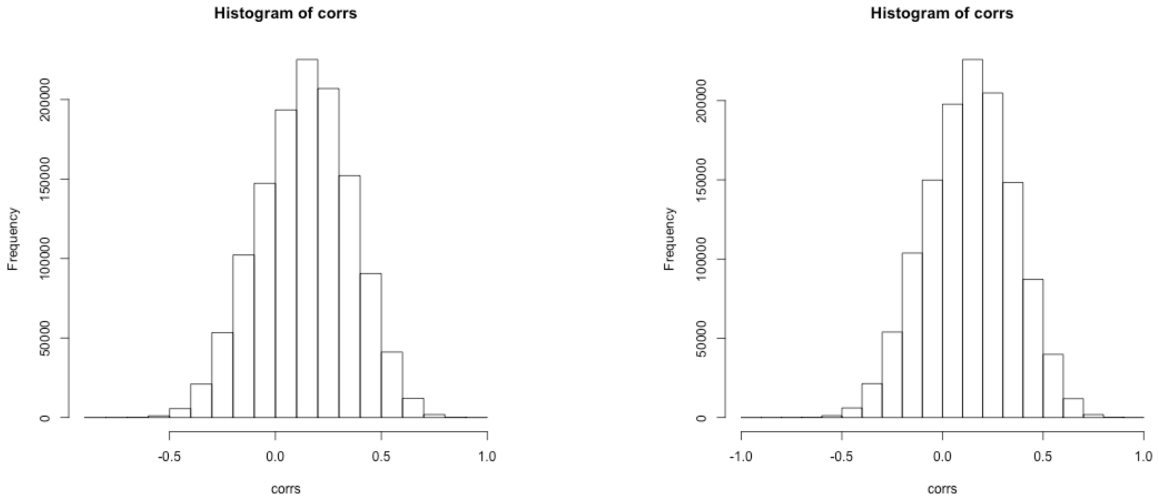

Figure S14: Correlation coefficient between ParasoR and PARS scores. Histograms of correlation coefficient between p_{stem} (**W=200** (Left) and **1,000** (Right)) and PARS score within the non-overlapping window of 20-nt length.

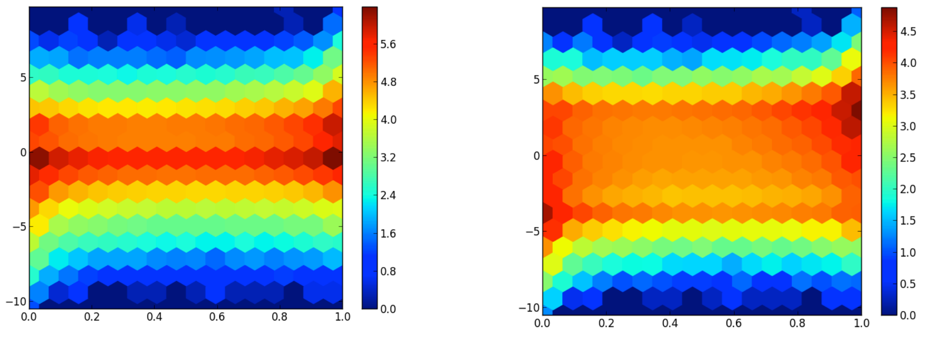

Figure S15: Relationship between ParasoR and PARS scores with or without filtering. Hexagonal binning plots for PARS scores (shown in y-axis) and p_{stem} (shown in x-axis) of **all positions** (Left) and **filtered positions** (Right) with the threshold 40 for the minimum read coverage of PARS score.

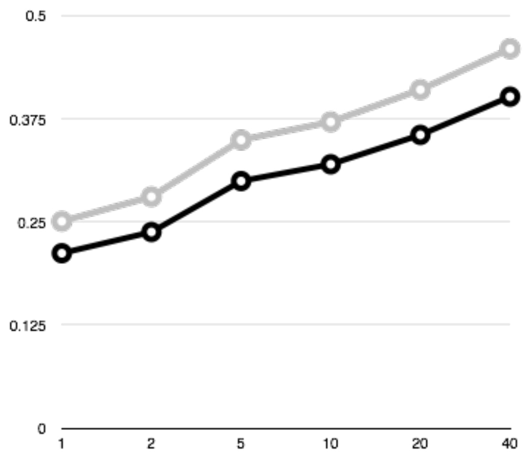

Figure S16: Correlation between ParasoR and PARS scores with filtering. Correlation coefficients between p_{stem} and PARS score filtered by setting the minimum read coverage shown in x-axis. The black and grey line represent correlation coefficients computed for stem probabilities based on Turner (2004) and Andronescu (2007), respectively.

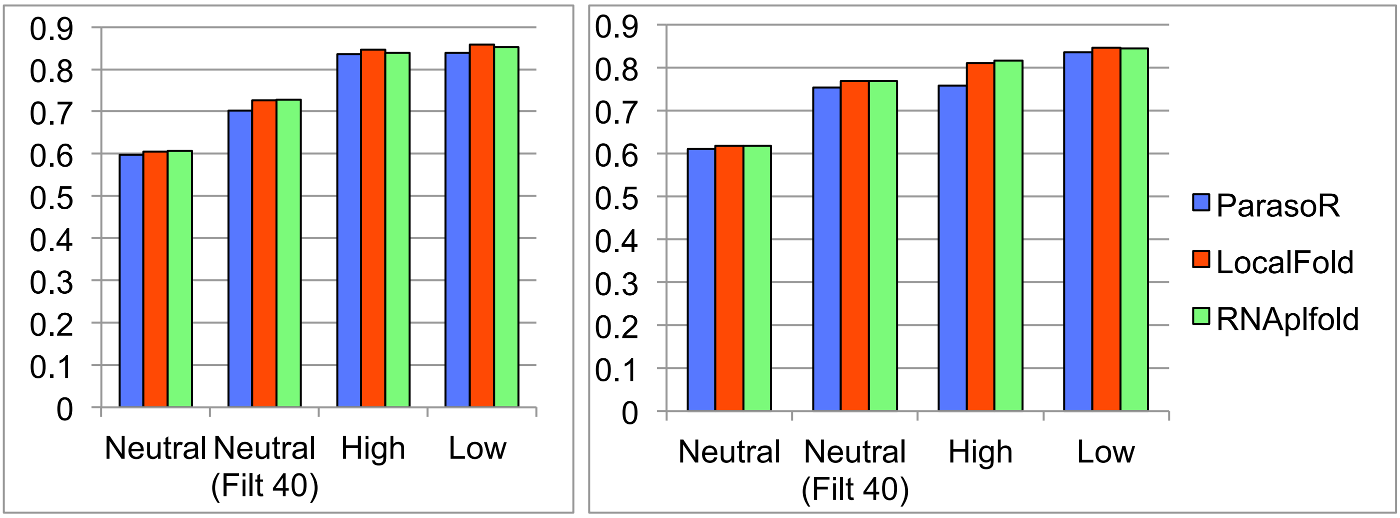

Figure S17: AUC scores for ROC curves between PARS and three tools. Each AUC was calculated for “true“ PARS score and “prediction” of three tools (p_{stem}) using (Left) **Turner (2004)** and (Right) **Andronescu (2007) energy model** for all positions, or limited positions where the read coverage of PARS is no less than 40 (used in Filt40). Neutral (-0.39), High (8.28), and Low (-9.06) corresponds to the threshold for PARS score used for classification of “accessible” or “structured” groups with progressively changing a threshold for the p_{stem}-based classification to calculate each AUC value.

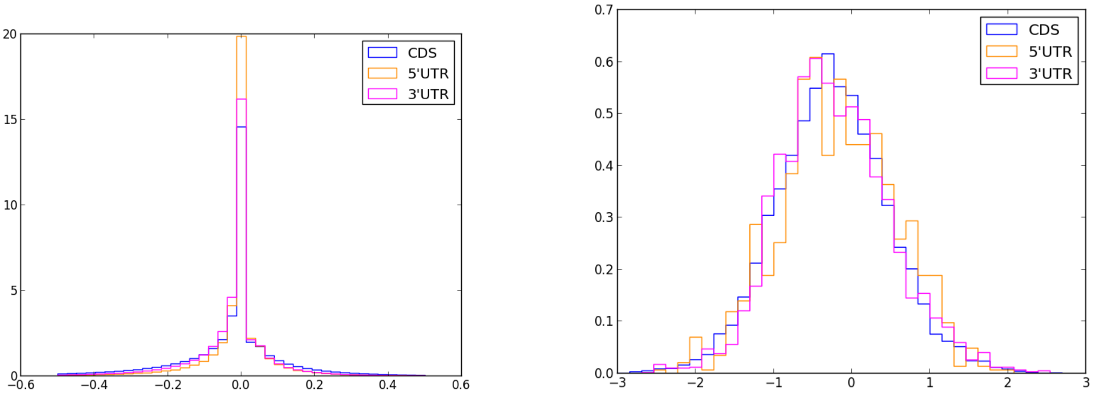

Figure S18: Histograms of PARS scores for each annotation (CDS, 5’UTR, and 3’UTR). Each figure shows the histograms **before** (Left) and **after filtering** (Right) with the threshold 1 for the minimum read coverage.

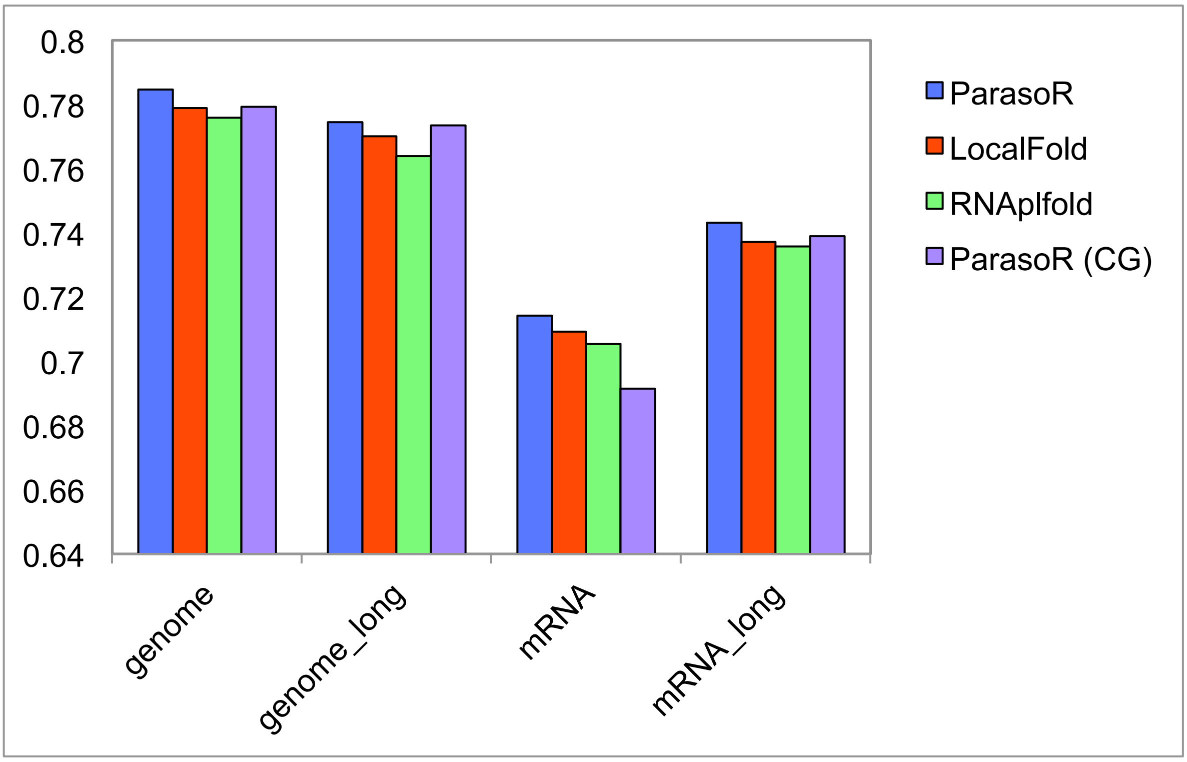

Figure S19: AUC scores of ROC curves between CisReg data and p_{stem}-based classification. Each position was classified into “accessible” or “structured” groups using three tools with Turner (2004) energy model, and ParasoR with Andronescu (2007) (CG) energy model. mRNA dataset is the set of cis-regulatory elements found on the known mRNA sequences in CisReg data, and genome dataset contains the others. The suffix “long” means that the sequences whose total length is less than 3000 nt are removed from the dataset.

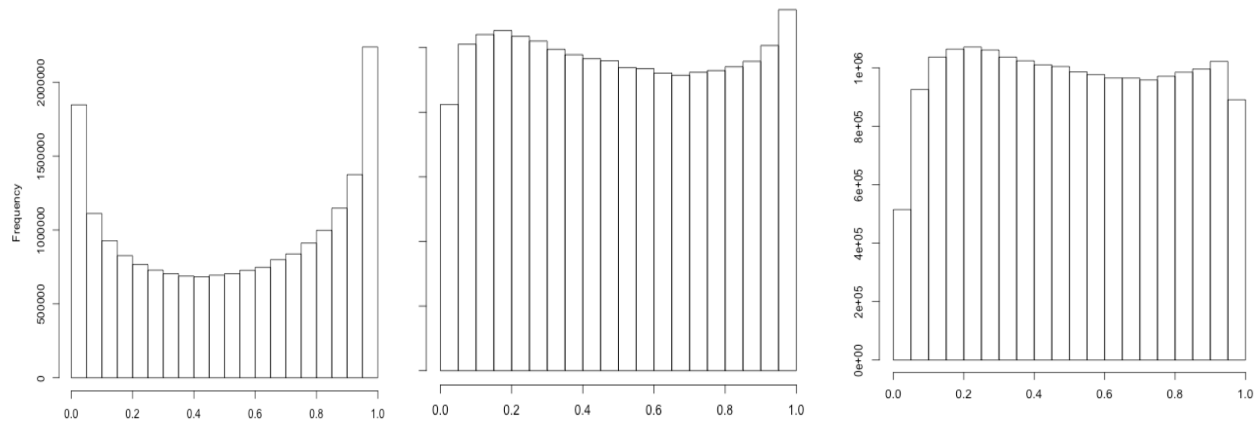

Figure S20: Histograms of p_{stem}. Each figure shows the histogram of p_{stem} calculated by ParasoR (Left), LocalFold (Center), and RNAplfold (Right) for human matured transcripts.

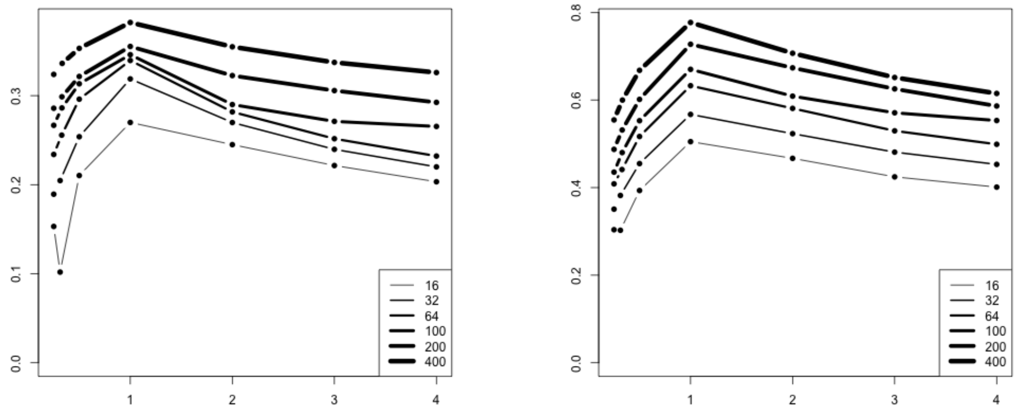

Figure S21: Influence of the different feature window size on linear regression. Correlation coefficients of averaged p_{stem} and the regressed value by L2 regression were computed using the feature of (Left) **GC contents** and (Right) **4-mer compositions** with different window sizes for feature calculation (shown in the legend). X-axes represent the relative window size for computing averaged p_{stem} and the regressed value to the window size for the feature selection.

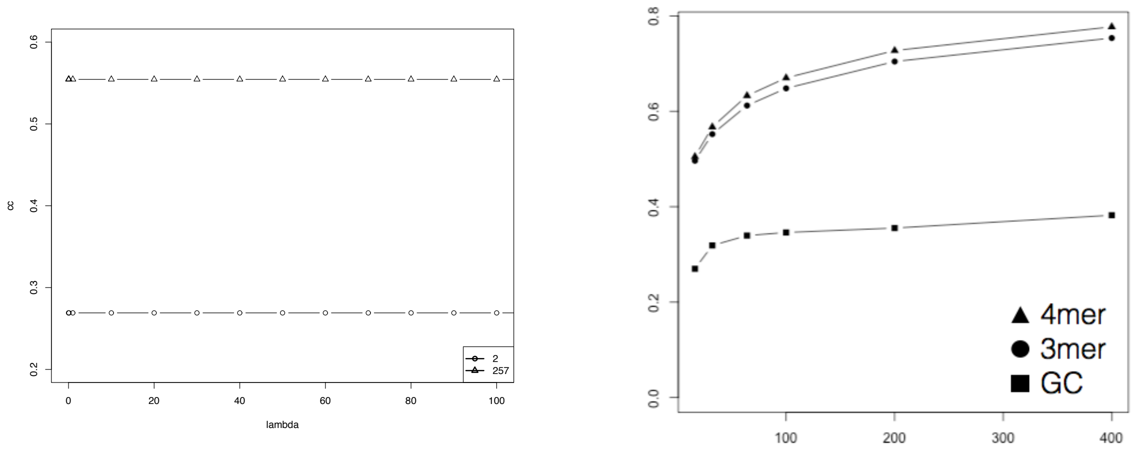

Figure S22: Influence of the regularization term and feature selection on linear regression. (Left) Correlation coefficients of averaged p_{stem} and the regressed value by L2 regression with **varying the regularization term** λ . For GC content (2) and 4-mer composition (257), λ has little influence on normalization efficiency. (Right) Correlation coefficients of averaged p_{stem} and the regressed value **with the different window size** for both of averaging and feature calculation (shown in x-axis).

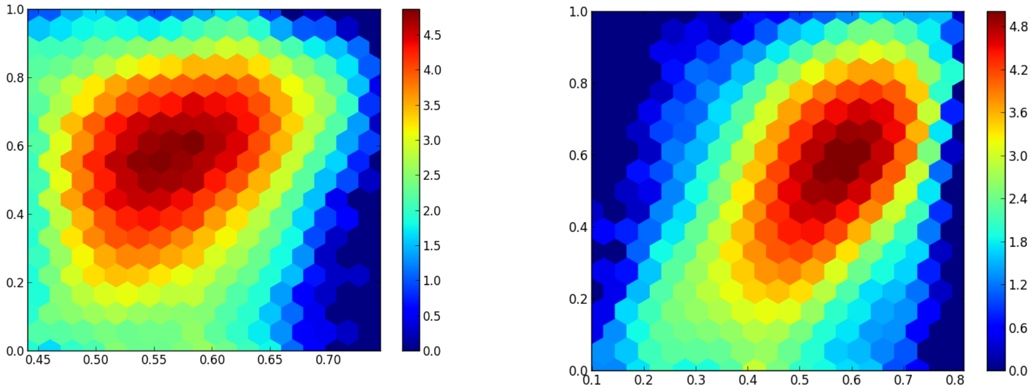

Figure S23: Efficiency of linear regression for stem probability. Hexagonal binning plots for **averaged p_{stem}** (y-axis) and **"predicted" stem probability of regression** (x-axis) with features of **GC content** (Left) and **4-mer** (Right) compositions.

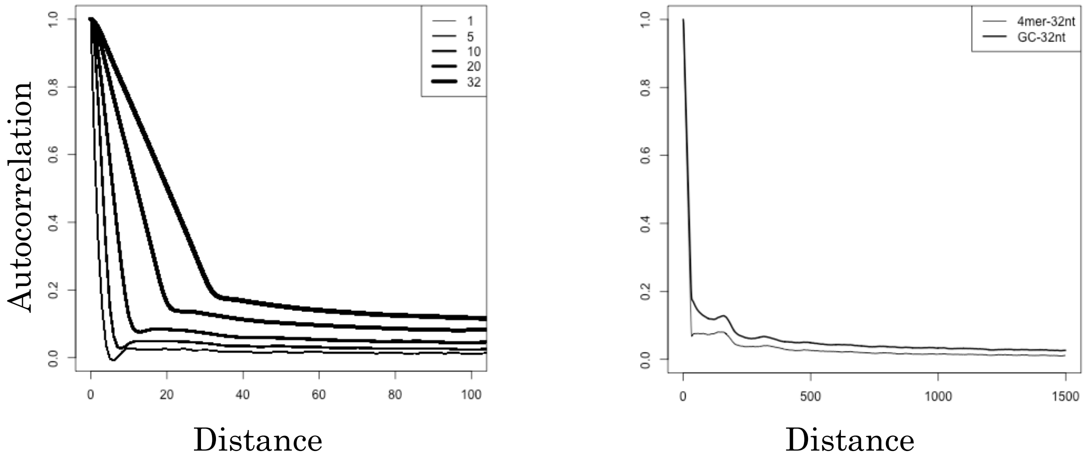

Figure S24: **Autocorrelations** of stem probability. Autocorrelations of (Left) **p_{stem}** and (Right) $\Delta\bar{\boldsymbol{p}}_{\boldsymbol{stem}}$ was computed using GC and 4-mer compositions as the feature of regression. The distance between two samples applied for the computation of autocorrelation is shown in x-axis.

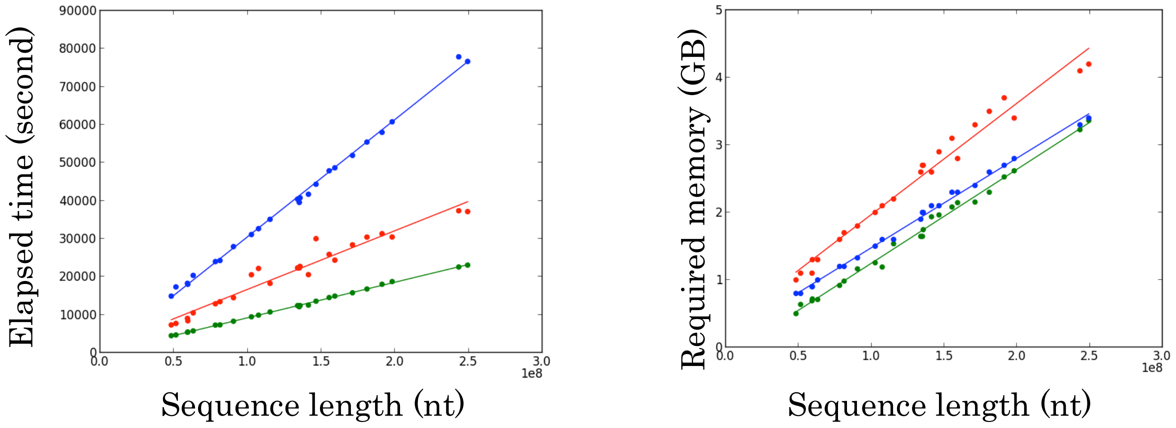

Figure S25: ParasoR performance. **Average elapsed times in seconds** (Left) and **required memory sizes in GB** (Right) for the simulation of each chromosome. P_{stem} was computed for the continuous chromosomes using at most 300 nodes in hgc super computer. X-axis represents the chromosome length (nt). The computation time and required memory was averaged for both strands as well as all of nodes during Divide (red), Connect (blue), and Probability calculation procedure (green).

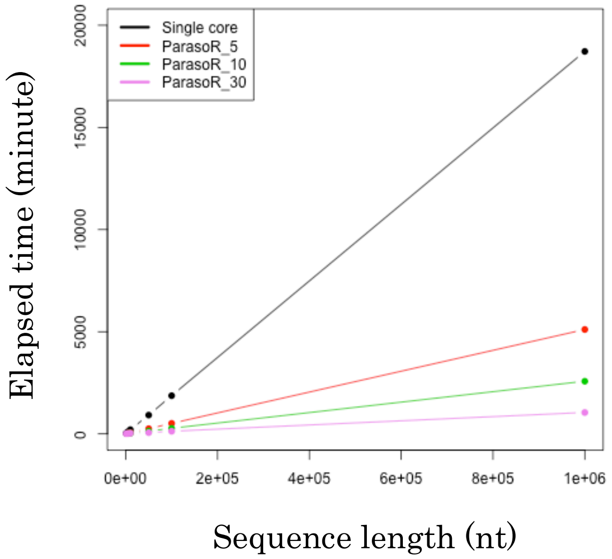

Figure S26: Relationship between **Elapsed time** (minutes) and the number of nodes. Elapsed time of ParasoR was measured with single core or multiple nodes (setting the maximal number of computing nodes to 5, 10, and 30) for 100 sequences. X-axis indicates the sequence length of the random sequences.

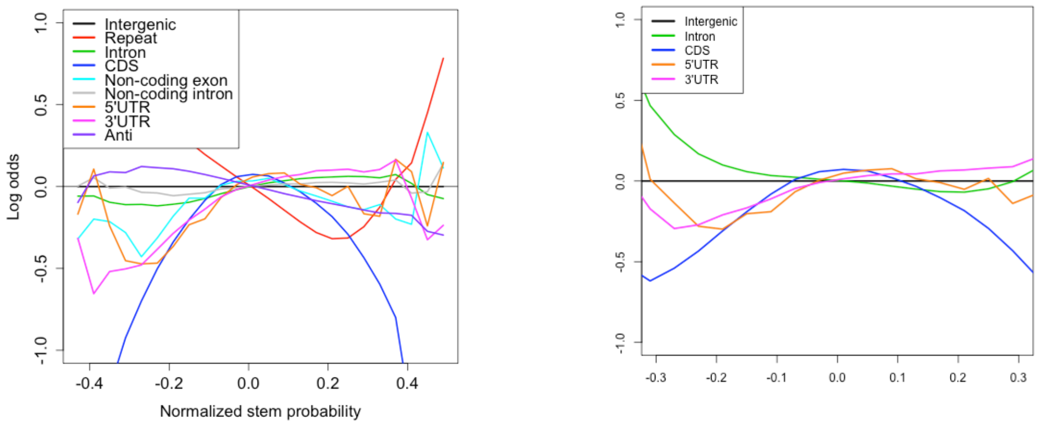

Figure S27: Structural propensity of each annotation group. (Left) Log odds ratios log(f(x)/f_{Intergenic}(x)) for $\Delta\bar{p}_{stem}$ distributions of **all of annotation groups** on human genome. Repeat region shows the specific tendency to have a peak far from 0. (Right) Log odds ratios for $\Delta\bar{p}_{stem}$ distributions of each annotation **without the removal of repetitive regions**.

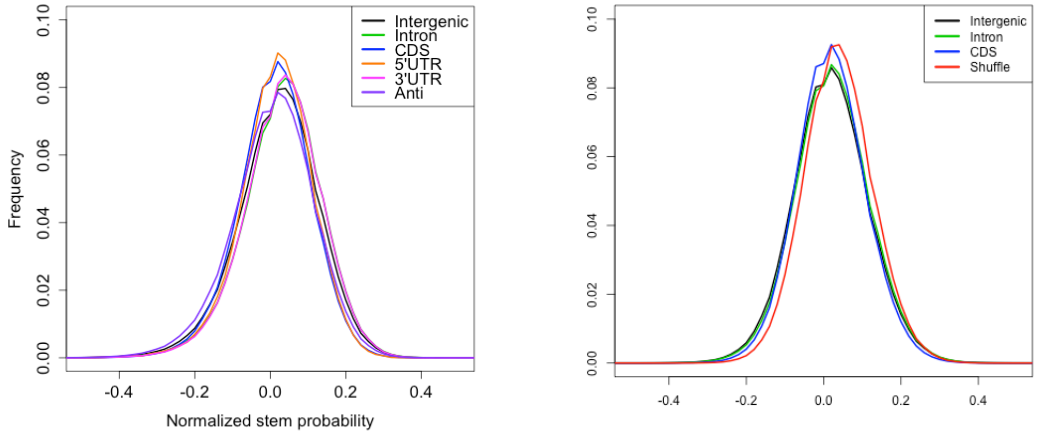

Figure S28: Structural propensity with other controls. (Left) Histogram of $\Delta\bar{p}_{stem}$ computed by **GC regression** for human genome. (Right) Histogram of $\Delta\bar{p}_{stem}$ using the **L2 regression with 4-mer compositions**. $\Delta\bar{p}_{stem}$ of **Shuffle region** was computed for the sequence of 1-mer shuffled chromosome 1 while those of the others were computed for original genome-sequence.

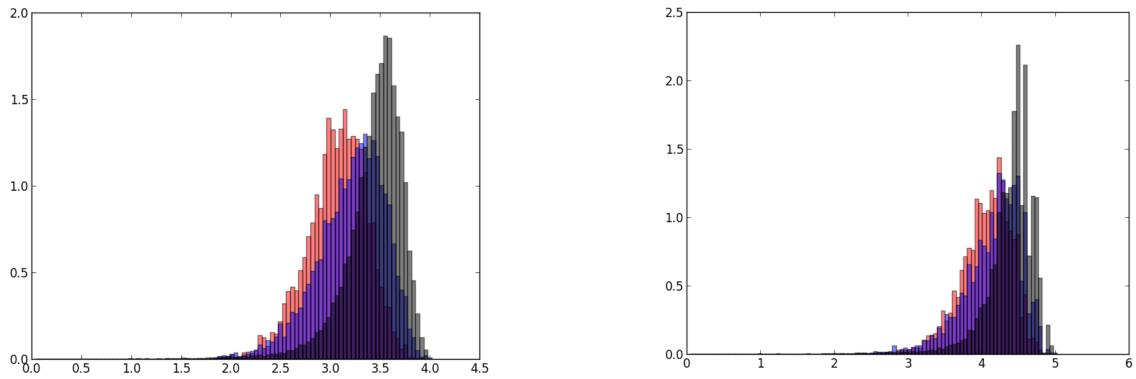

Figure S29: Entropy distribution. Histograms of the entropy of **2-mer** (Left) and **3-mer** (Right) compositions for each 32-mer fragment on human chromosome 1. Both of images exhibit the entropy distribution for whole fragments (black), structured fragments where $\Delta\bar{p}_{stem}$ > 0.3 (blue), and accessible fragments where $\Delta\bar{p}_{stem}$< -0.3.

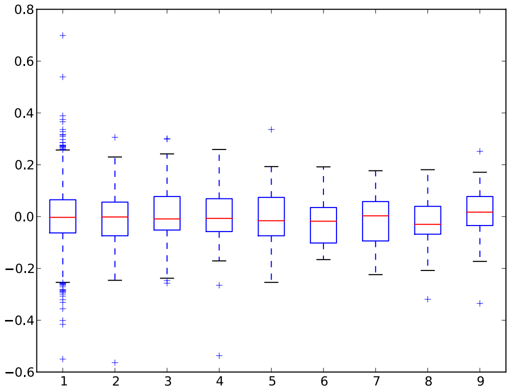

Figure S30: Relationship between structure propensity and conservation score. Boxplots of $\Delta\bar{p}_{stem}$ binned by the conservation score of each fragment. X-axis shows 10 groups classified by PhastCons score in steps of 0.1 from 0 to 1. Between PhastCons score and $\Delta\bar{p}_{stem}$, we detected no significant correlation ($p > 0.05$).

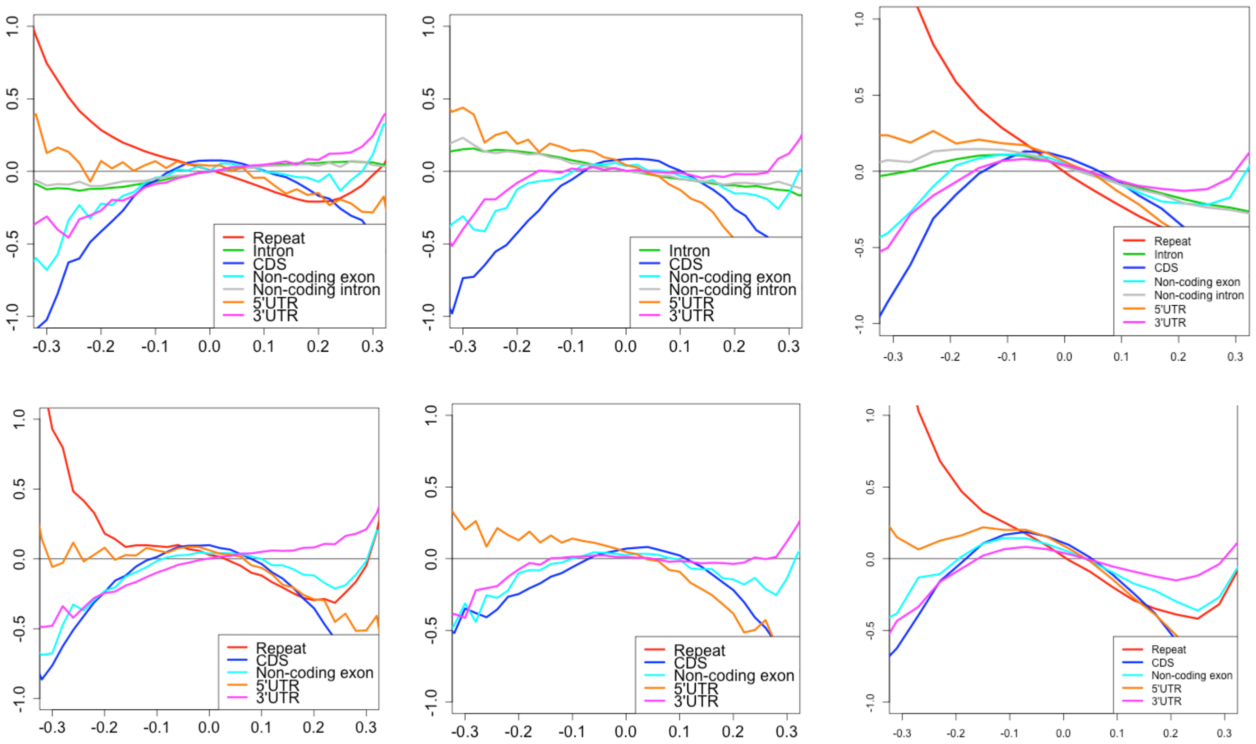

Figure S31: Structure propensity of each annotation group with different conditions. Log odds ratios log(f(x)/f_{Sense Intergenic}(x)) for $\Delta\bar{p}_{stem}$ distributions of each annotation on **pre-mRNA** (Upper) and **mRNA** (Lower) of **sense sequence in human** genome (Left), **antisense sequence in human** genome (Center), and **sense sequence in mouse** genome (Right). 5'UTR shows an elevation of log odds ratios at the region of positive x, indicating that the group contains the larger ratio of accessible fragments compared to that of the Intergenic group.

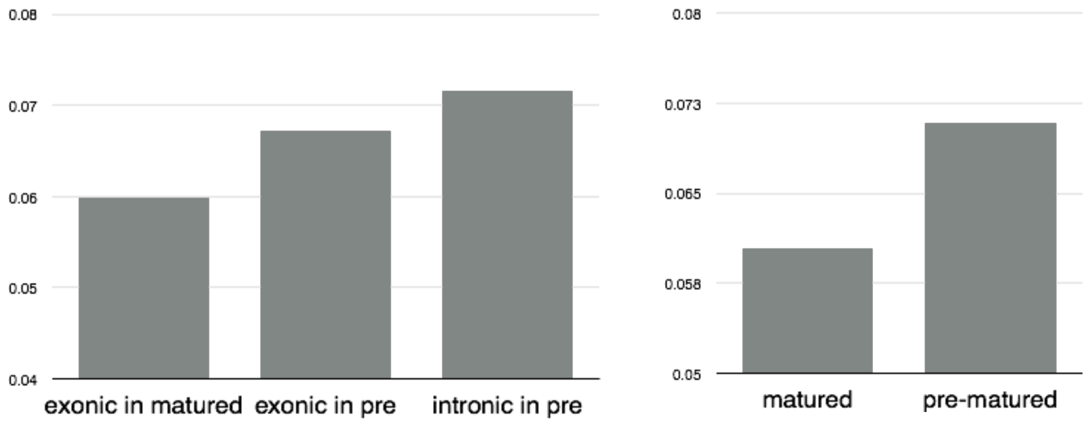

Figure S32: Structure propensity of transcripts with other controls. Kolmogorov-Smirnov's D statistics for $\Delta\bar{p}_{stem}$ distributions of exonic and intronic regions **sense and anti-sense transcripts** (Left) and $\Delta\bar{p}_{stem}$ distributions of **sense and 3-mer shuffled sense transcripts** with (pre-matured) or without (matured) introns (Right).

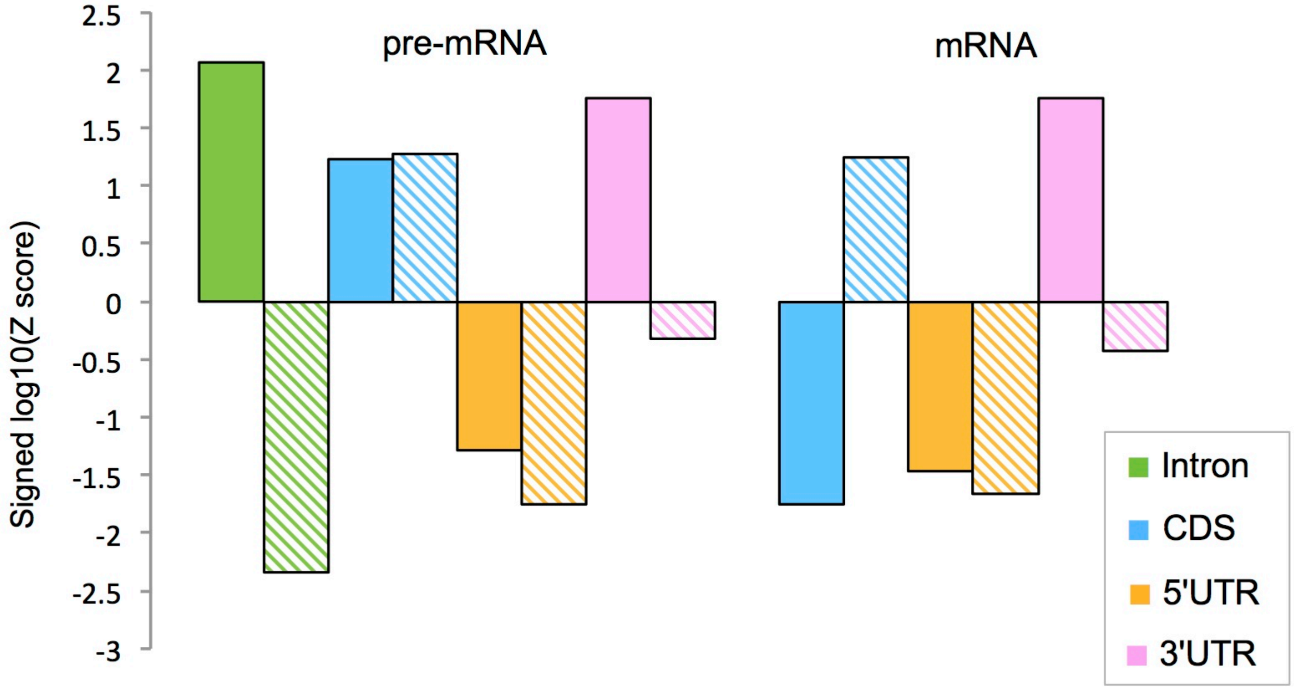

Figure S33: Structure propensity of transcripts in mouse genome. Z-scores of Wilcoxon rank sum test for $\Delta\bar{p}_{stem}$ distribution between Intergenic and each annotation on pre-mRNA and mRNA in **mouse genome**.

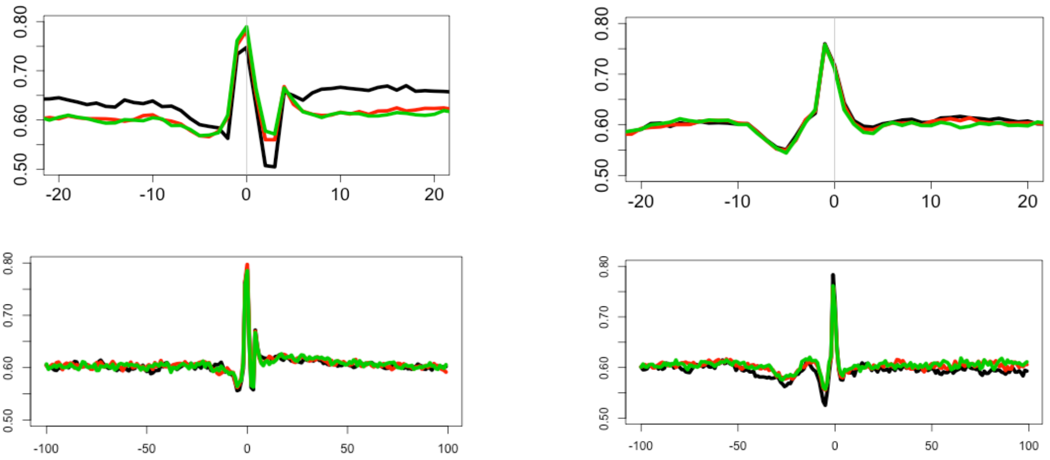

Figure S34: Regional profiles of average stem probability. Stem probability was averaged for each position around **donor site** (Left) and **acceptor site** (Right) in two different scales for the last 3 SSs on pre-mRNA (1st, 2nd, and 3rd splice site is indicated by the black, red, and green line).

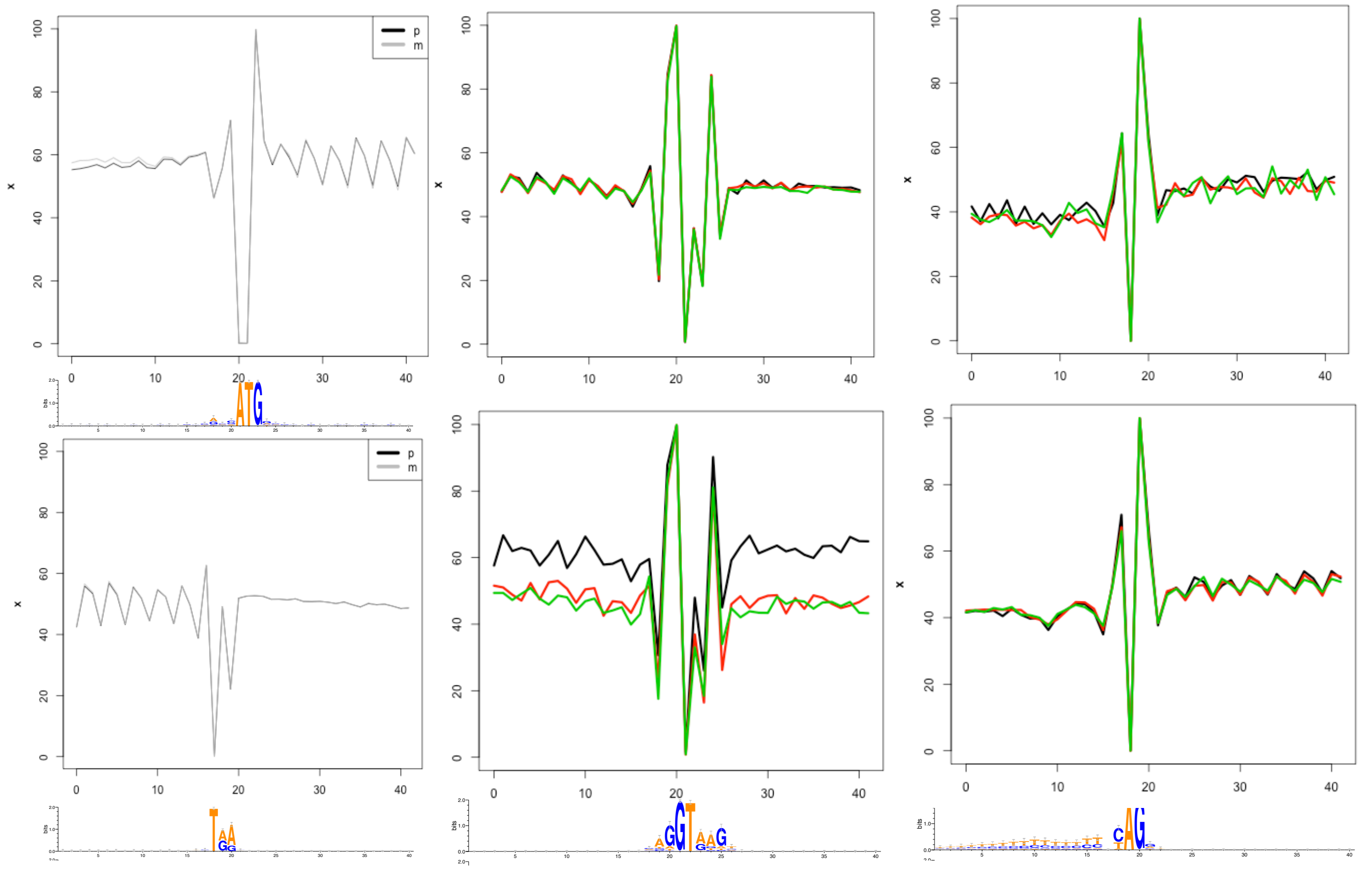

Figure S35: Profiles of GC content. (Left) Profiles of GC contents around **start codon** (Upper) and **stop codon** (Lower). (Center and Right) Profiles of GC contents around **donor site** (Center) and **acceptor site** (Right) of pre-mRNA averaged for **the 1st, 2nd, and 3rd splice site** (black, red, and green) **from the front** (Upper) and **from the back** (Lower). Sequence logos were constructed for all of pre-mRNA sequences.

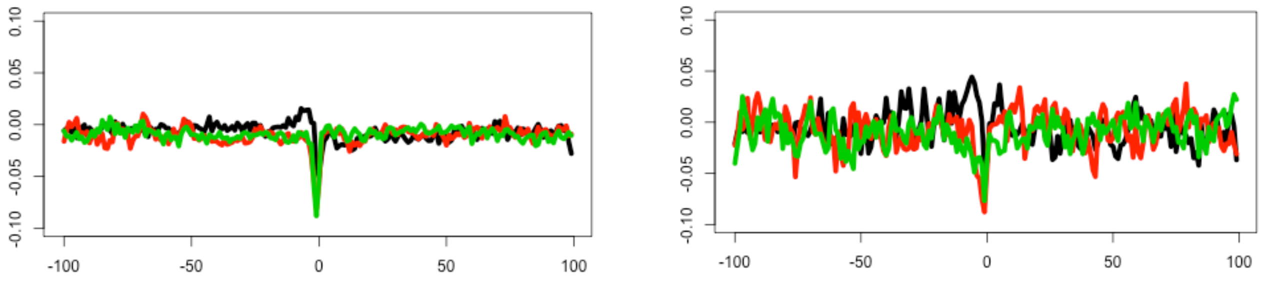

Figure S36: Regional profiles of difference of stem probability. Mean differences of stem probability were computed around the exon junction for **mRNA** (Left) and **non-coding RNA** (Right) **between pre-mRNA and mRNA for the 1st, 2nd, and 3rd splice site** (represented by a black, red, and green line, respectively).

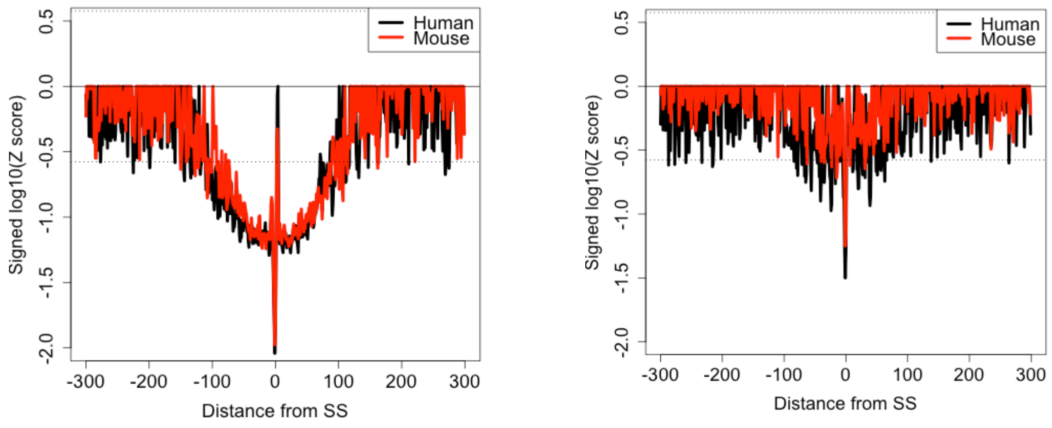

Figure S37: Structure disruption caused by splicing. Z-score profiles by Wilcoxon's rank sum test for difference of stem probability around splice site of **mRNA** (Left) and **non-coding RNA** (Right).

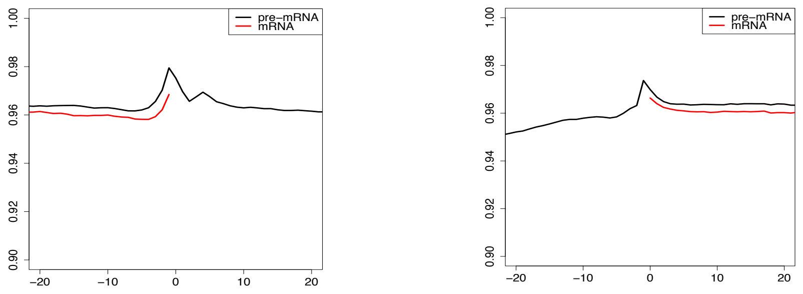

Figure S38: Propensity of structural interaction. Profiles of the probability of crossing base pairs at each position around donor (Left) and acceptor site (Right). That probability corresponds to (1-probability that neighbor two bases belong to an exterior loop or end of the outermost base pair).

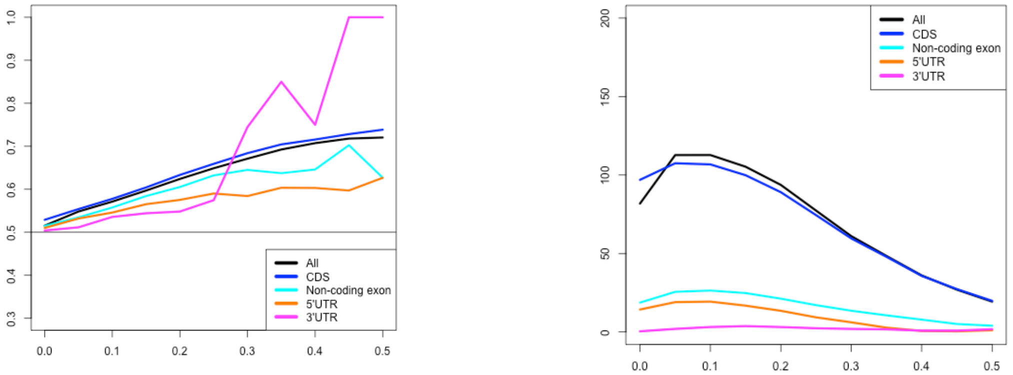

Figure S39: Structural difference caused by splicing. (Left) **Ratios of post-accessible fragments** and (Right) **–log10(Z-scores)** computed by Wilcoxon's signed rank test for the difference of p_{stem} on mRNAs or matured non-coding RNAs in 5 annotation groups in human. X-axis represents the threshold for filtering samples where the absolute difference of p_{stem} is lower than the threshold. This filtering process enables to only retain substantially different samples in terms of p_{stem}.

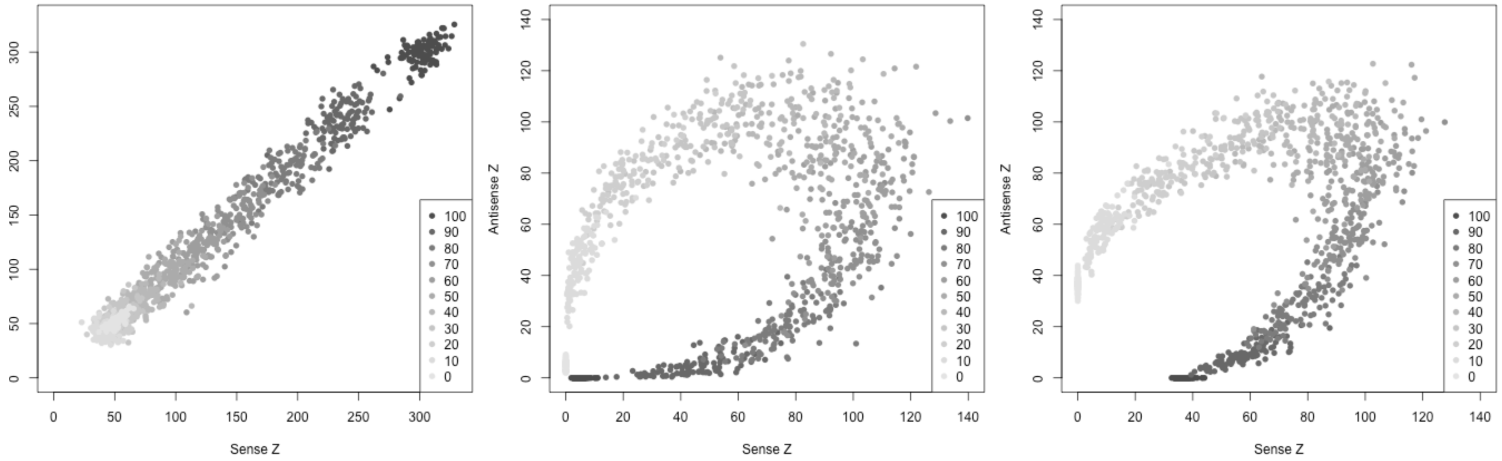

Turner (2004)

Andronescu (2007)

Figure S40: Structural bias between sense and antisense strand. Partition function of sense and antisense random sequences of 200-nt long using different energy models. To produce random sequences, **GC content** (Left) and **GU content** (Center and Right) were altered from 0 to 100% (shown in legend).

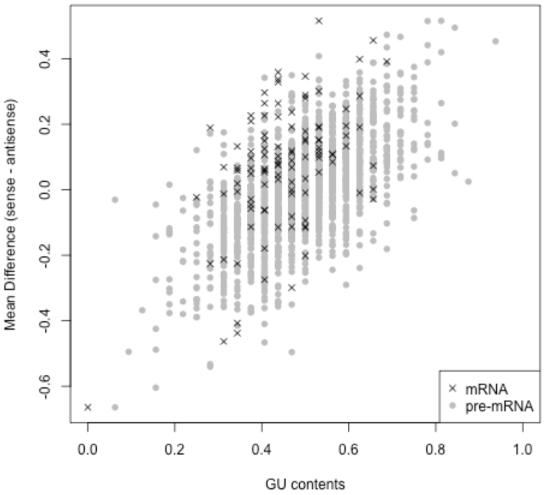

Figure S41: Structural bias of mRNA and pre-mRNA between sense and antisense strand. Mean difference of p_{stem} between sense and antisense structure of *NEXN* gene (NM_144573) for each window. X-axis represents GU content of each 32-nt window. Y-axis shows the mean difference of stem probability (p_{stem,sense} – p_{stem,antisense}) of each single base within the window.

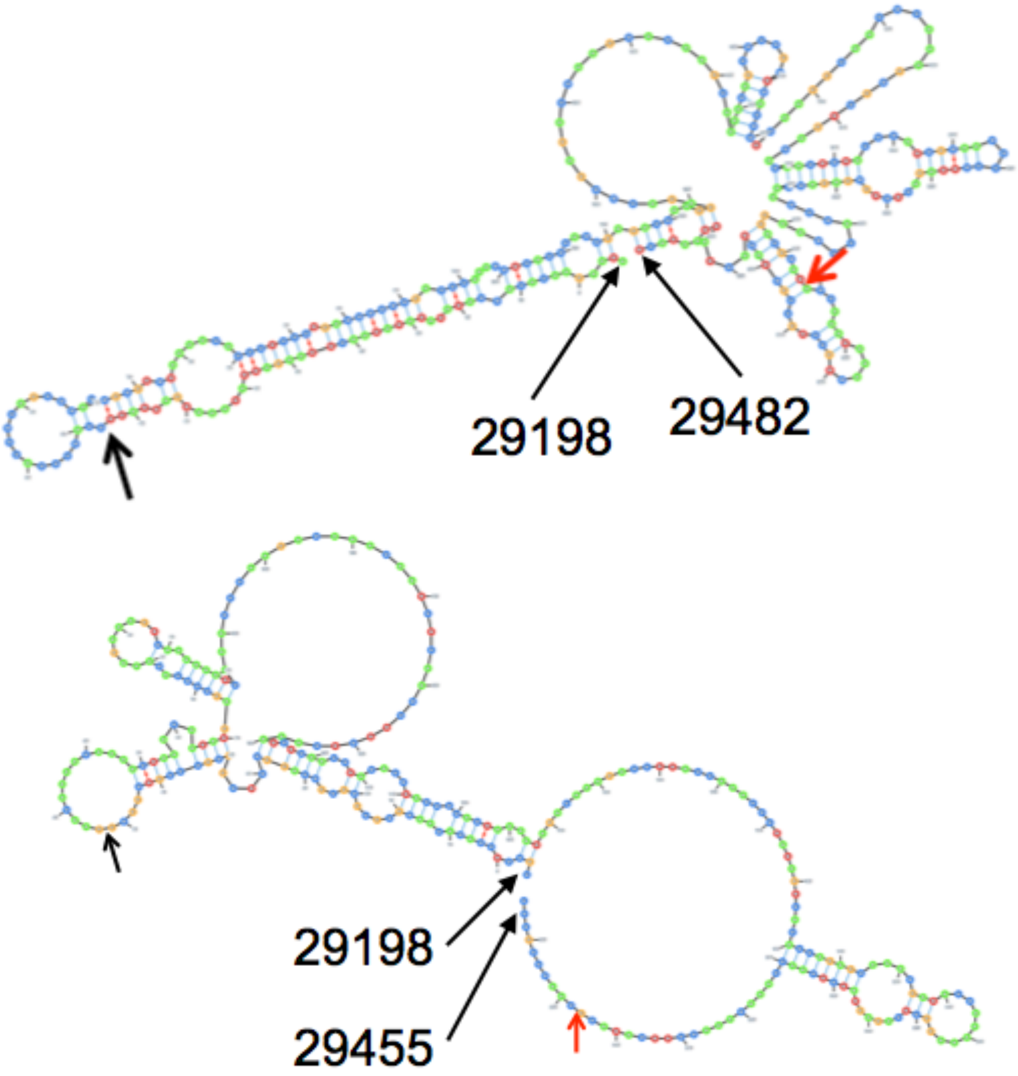

Figure S42: Predicted structure of NEXN in sense and antisense strand. Partial γ-centroid structure of sense (Upper) and antisense (Lower) pre-mRNA of *NEXN* gene (NM_144573) around 3rd intron (29244-29445). Black and red arrows correspond to the donor and acceptor site in sense strand, respectively. For comparison, we reversed the antisense sequence and structure. Hence, each position in antisense strand indicates the position of the complementary base to that of sense strand.

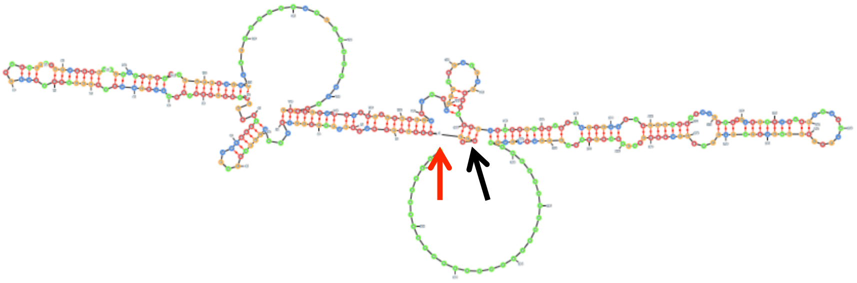

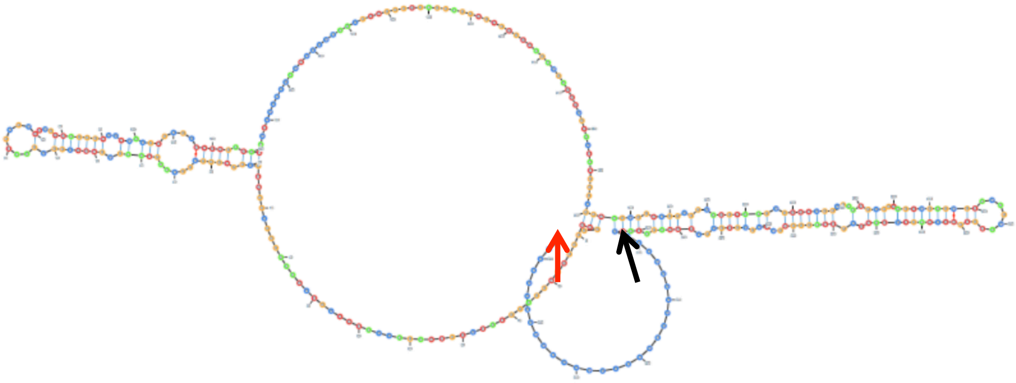

Figure S43: Predicted structure of an Alu sequence in sense and antisense strand. γ -centroid structure of sense (Upper) and antisense (Lower) consensus sequence of the Alu-Jo subfamily. Black and red arrows correspond to the start and end point in sense strand, respectively. As in Figure S42, we reversed the antisense sequence and structure.
